# Effects of Different SNP Calling and Sequence Mapping Choices on the Inference of Genetic Architecture Underlying Migration Tendency

**DOI:** 10.1002/ece3.73743

**Published:** 2026-05-27

**Authors:** Giovanna Mottola, Frank Panitz, Tuomas Leinonen, Alexandre Lemopoulos, Anssi Vainikka

**Affiliations:** ^1^ Department of Environmental and Biological Sciences University of Eastern Finland Joensuu Finland; ^2^ Genomics and Breeding Unit (GEJA) Natural Resources Institute Finland (LUKE) Turku Finland; ^3^ Natural Resources Unit (LUVA) Natural Resources Institute Finland (LUKE) Turku Finland; ^4^ Genomics and Breeding Unit (GEJA) Natural Resources Institute Finland (LUKE) Helsinki Finland

**Keywords:** brown trout, dDocent, life‐history, outlier, reference genome, *Salmo trutta*, stacks

## Abstract

Genome‐wide association studies with identification of biologically relevant genes rely on correct mapping of sequence variation. Here, we re‐analysed RADseq data from two migration types of brown trout ( *Salmo trutta* ) from Koutajoki and Oulujoki watersheds by replacing the originally applied Atlantic salmon ( *Salmo salar* ) reference genome with the later published brown trout reference genome and by testing three alternative bioinformatic pipelines for identifying single nucleotide polymorphisms (SNPs). As expected, the results from population genomics and outlier analyses largely confirmed the original patterns of population structure and divergence, although the number of called SNPs varied between the used bioinformatic pipelines and reference genomes and was surprisingly lower with the conspecific reference genome. While only two SNP outliers were found by all the alternative methods, several other outlier SNPs related to migration differences among the populations were identified. These findings confirm that the choice of the reference genome is not critical for the inference of population structures but can improve the reliability of candidate gene identification in brown trout.

## Graphical abstract

We re‐analysed RAD‐Seq data from brown trout using a conspecific reference genome and alternative SNP pipelines. Population structure was robust across methods, but using the brown trout reference improved detection of biologically relevant outlier SNPs.

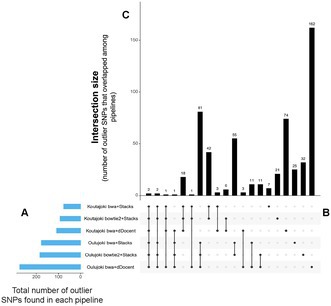

## 1 Introduction

Conservation of genetic diversity as the ultimate level of biodiversity has become increasingly important in the recent decades as human‐driven habitat fragmentation, environmental degradation and overharvesting have led to significant population declines of many iconic species globally (Fischer and Lindenmayer 2007; Frankham 2010; Heinrichs et al. 2016; Hohenlohe et al. 2021; IPBES 2019). Genetic diversity is hierarchically distributed between individuals, subpopulations, metapopulations and species, with important structuring effects from key life‐history differences such as those relating to migratory behaviour in brown trout ( *Salmo trutta* ) (Lemopoulos, Uusi‐Heikkilä, Huusko, et al. 2018). While understanding the patterns of genetic variation in the wild is essential for species conservation, it is likewise important to understand the genetic architecture of ecologically significant life‐history variation for the conservation of ecosystem functionality. Salmonids, like *Salmo* spp., *Oncorhynchus* spp. and *Salvelinus* spp., are economically and culturally significant fishes that often form distinctively resident or migratory populations with significant associated differences in several other life‐history traits (Vera et al. 2023). Major efforts have been put into the assembly of their reference genomes and identification of genetic regions that could facilitate genomic selection, for example for growth and flesh quality in salmon aquaculture (Houston and Macqueen 2019). Among the first assembled salmonid reference genomes was that of the Atlantic salmon ( *Salmo salar* ) (Davidson et al. 2010; Lien et al. 2016), which has until recently been used as a reference for the rest of Salmonidae (Davidson 2013; Elias et al. 2018; Lemopoulos, Uusi‐Heikkilä, Huusko, et al. 2018; Lemopoulos, Uusi‐Heikkilä, Vasemägi, et al. 2018; Lemopoulos, Uusi‐Heikkilä, et al. 2019; Lemopoulos, Prokkola, et al. 2019; Robinson et al. 2017). Since then, reference genomes have been published for brown trout ( *Salmo trutta* ) (Hansen et al. 2021), rainbow trout ( *Oncorhynchus mykiss* ) (Gao et al. 2021), Coho salmon ( *Oncorhynchus kisutch* ) (https://www.ncbi.nlm.nih.gov/assembly/GCF_002021735.2/) and European whitefish ( *Coregonus lavaretus* ) (Gao et al. 2021; Pokharel et al. 2025) with rapid developments in understanding the genetic architecture of multiple traits (Clare et al. 2023; Gallardo‐Hidalgo et al. 2021).

Migration tendency, as a trait separating resident populations from migratory (anadromous or potamodromous) populations, is partly driven by inherited variation (Ferguson et al. 2019). Multiple studies on brown trout have shown that migratory tendency is a threshold trait that is proximately affected also by a number of environmental factors, with significant genotype‐by‐environment (G × E) interactions (Olsson et al. 2006; Ferguson et al. 2016, 2019; Nevoux et al. 2019). Before the publication of the brown trout reference genome (Hansen et al. 2021), genomic studies of brown trout relied on *de novo* assembly or on heterospecific alignment to the *S. salar* reference genome (Leitwein et al. 2016; Lemopoulos, Uusi‐Heikkilä, Huusko, et al. 2018; Lemopoulos, Uusi‐Heikkilä, Vasemägi, et al. 2018). The average nucleotide divergence observed between *S. salar* and *S. trutta* is 5.94% for mitochondrial DNA and the estimated average net nucleotide divergence (d_*a*_) is 0.0187 for genomic DNA (Bernatchez et al. 1992; Leitwein et al. 2016, 2017). The brown trout studies using *S. salar* reference assembly have identified variants that differentiate hatchery fish from wild ones (Leitwein et al. 2016), as well as genomic regions related to variation in smoltification and osmoregulation functions (Lemopoulos, Uusi‐Heikkilä, Huusko, et al. 2018). However, no individual genes with large effects on migratory behaviour have been identified to date leaving it a question whether the trait itself is polygenic or whether some regulatory master genes with large effects have not yet been found (Lemopoulos, Uusi‐Heikkilä, et al. 2019).

Reference genomes from phylogenetically close species (i.e., heterospecific mapping) have been valuable in early research of species‐specific functional genomics, but conspecific mapping offers higher accuracy and reliability (Shafer et al. 2017) and limits the occurrence of false positives (Manel et al. 2016). The increased quality of the modern genome assemblies obtained through long‐read sequencing will also increase the possibilities for genome‐wide association studies in new species (Bringloe and Parent 2023). Lemopoulos, Uusi‐Heikkilä, Huusko, et al. (2018), used the Atlantic salmon ( *S. salar* ) reference genome to study genomic associations of migratory behaviour in brown trout. While this study found some candidate SNPs with relevant functions in osmoregulation and immune defence, it left a technical need to re‐examine the obtained sequence data with the now available brown trout reference genome (Hansen et al. 2021). Many variant calling pipelines have been developed over the last two decades for handling Restriction‐site Associated DNA (RADseq) data (Catchen et al. 2013; Garrison and Marth 2012; Puritz et al. 2014; Rochette and Catchen 2017). Also, the choice of variant‐calling pipeline introduces variability in genomic studies, as each pipeline contains unique bioinformatic algorithms and methods (Shafer et al. 2017; Wright et al. 2019). Lemopoulos, Uusi‐Heikkilä, Huusko, et al. (2018) used Stacks v1 (Catchen et al. 2013) that supports single end‐reads, demultiplexing, assembling loci within and across individuals and conducting population genetic analyses. The updated version, Stacks v2 (Rochette et al. 2019), adds support for paired‐end reads, processes loci across all individuals simultaneously within a metapopulation, and uses a Bayesian genotype caller to detect SNPs. Another major software, dDocent (Puritz et al. 2014) also uses paired‐end reads throughout and offers flexibility by integrating existing bioinformatics tools, which includes FreeBayes in the entire workflow (Garrison and Marth 2012). FreeBayes, like Stacks v2, is based on a Bayesian approach to variant calling. Both of these pipelines offer high quality SNP calling and have been extensively used for studying population genomics of fish (Brauer et al. 2016; Catchen et al. 2013).

In the present study, our aim was to examine the technical effects of alternative SNP identification methods and determine whether the use of the conspecific reference genome (Hansen et al. 2021) would change the list of candidate genes identified to comparatively explain the migratory differences of brown trout populations (Lemopoulos, Uusi‐Heikkilä, Huusko, et al. 2018). To re‐estimate the population structure for confirmation (Lemopoulos, Uusi‐Heikkilä, Vasemägi, et al. 2018), and to compare migratory and resident populations of brown trout from two watersheds (Lemopoulos, Uusi‐Heikkilä, Huusko, et al. 2018), we ran two different SNP‐calling pipelines, dDocent and the newest version of Stacks (v2) on the publicly available sequence data. We predicted that the change of the reference genome would not change the inferred population structure as reported by Lemopoulos, Uusi‐Heikkilä, Vasemägi, et al. (2018) but it could refine the candidate gene list for brown trout migratory tendency (Lemopoulos, Uusi‐Heikkilä, Huusko, et al. 2018).

## 2 Material and Methods

### 2.1 Sequence Data

**FIGURE 1:**
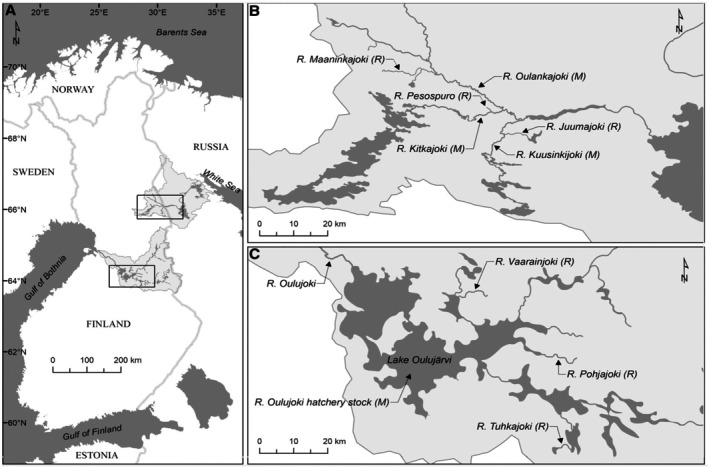
Map showing the study areas in Finland (A) and specifically the Koutajoki (B) and Oulujoki (C) watersheds where fish were collected. The current image is an adaptation of a previous map published in Lemopoulos, Uusi‐Heikkilä, Huusko, et al. (2018).

We used published RADseq data (GenBank accession numbers: Koutajoki: PRJNA431174; Oulujoki: SRP125540) from Lemopoulos, Uusi‐Heikkilä, Huusko, et al. (2018) and Lemopoulos, Uusi‐Heikkilä, Vasemägi, et al. (2018). The RAD‐sequenced brown trout samples represented both migratory and resident populations sampled between 2010 and 2015 in two watersheds in North‐Eastern Finland (for maps and description of these systems, see Lemopoulos, Uusi‐Heikkilä, Huusko, et al. 2018; Lemopoulos, Prokkola, et al. 2019): Koutajoki (resident: MAA, PES, JUU; migratory: OUL, KIT, KUU) and Oulujoki (resident: VAA, POH, TUH; migratory: OUV; Table 1). The resident individuals were sampled in small tributaries, while the migratory fish represented main stems and a hatchery population in Oulujoki (Figure 1; Table 1).

**TABLE 1:** List showing the *N*‐value of all the sequenced individuals, their behaviour (migratory or resident) and from which rivers and watershed they were sampled.

Watershed	River	Code	Behaviour	*N*
Koutajoki	Maaninkajoki	MAA	R	30
	Pesospuro	PES	R	30
	Juumajoki	JUU	R	30
	Kitkajoki	KIT	M	29
	Oulankajoki	OUL	M	30
	Kuusinkijoki	KUU	M	30
Oulujoki	Vaarainijoki	VAA	R	29
	Pohjajoki	POH	R	12
	Tuhkajoki	TUH	R	11
	Oulujoki hatchery stock	OUV	M	28

### 2.2 Alignment and SNP Calling

To separate the effects of using different reference genomes from the effects caused by the variant‐calling pipelines, we applied two distinct SNP calling methods, dDocent v. 2.9.4 (Puritz et al. 2014) and Stacks v.2.65 (Rochette et al. 2019; Rochette and Catchen 2017), for the both sequence datasets. Since dDocent includes only *bwa* as alignment software and Lemopoulos, Uusi‐Heikkilä, Huusko, et al. (2018) used *bowtie2*, we ran Stacks v.2.65 using both *bowtie2* and *bwa* as alignment software to account for variability arising from this difference. The SNPs obtained using these pipelines were examined for overlap using the command *vcftoolz compare* in vcftoolz v. 1.2.3 (Davis 2019).

The Koutajoki library was demultiplexed using *process_radtags* command present in Stacks v.2.65, while the Oulujoki library was already demultiplexed. Following the protocol in Lemopoulos, Uusi‐Heikkilä, Huusko, et al. (2018), for *process_radtags*, we added the *–inline_null* argument to specify that the barcodes were inline with sequence. We also added the *–renz_1* pstI and *–renz_2* bamHI, which were the restriction enzymes used for the library preparation. Argument *‐q* was added to discard reads with low quality scores while the *‐i* argument was added to specify the input file type. Also, the *‐c* argument was added to clean data and remove any reads with an uncalled base and *‐r* argument to rescue barcodes and RAD‐Tag cut sites. Since the barcodes length spanned from 9 to 13 bp, we truncated the reads to 85 bp by using the argument *‐t* 85. Successively, the raw data were fed in dDocent workflow and Stacks. In dDocent, the raw data was aligned to the *S. trutta* reference genome (fSalTru1.1, GCA_901001165.1) using *bwa* v0.7.17 and the successive indexing was done using *samtools* v1.17. The .bam files obtained were then used to call variants, both SNPs and INDELs, using FreeBayes v.1.3.6 (Garrison and Marth 2012). The bioinformatic protocol with Stacks 2.65 followed Lemopoulos, Uusi‐Heikkilä, Huusko, et al. (2018). The RADseq raw data were first aligned to the *S. trutta* reference genome (fSalTru1.1, GCA_901001165.1) using *bowtie2* and *bwa*. For *bowtie2* the arguments used were *‐‐end‐to‐end* (involving every character of the reads) and *‐‐very‐sensitive* as set previously by Lemopoulos, Uusi‐Heikkilä, Huusko, et al. (2018), while the default settings were used when aligning using *bwa mem*. The .sam files obtained from the alignment were indexed and sorted using *samtools* v. 1.21 (Danecek et al. 2021). The resulting .bam files were then fed in the *gstacks*, including the population information as argument, and *population* modules, which were used to call variants. For the population module we followed Lemopoulos, Uusi‐Heikkilä, Huusko, et al. (2018), i.e., the variants were called using the following filtering options: *‐r 0.6 (‐‐min‐samples‐per‐pop 0.6*) which indicates that a minimum of 60% individuals in a population should be required to process a locus. *‐p 6/4* (*‐‐min‐populations 6/4*), which is the minimum number of populations a locus must be present in to process that locus. This parameter was set to 6 populations for Koutajoki and 4 populations for Oulujoki. Additionally, SNPs with a minor allele frequency of at least 0.05 (‐‐min‐maf 0.05) and maximum heterozygosity of 0.5 (‐‐max‐obs‐het 0.5) were retained.

### 2.3 Genome Scan, F‐Statistics and Structure Analyses

All the datasets were filtered for Hardy–Weinberg equilibrium (HWE) population‐wise to remove the SNPs (2868 for Koutajoki bwa+dDocent, 2195 for Oulujoki bwa+dDocent, 1826 for Koutajoki bowtie2+Stacks and 664 for Oulujoki bowtie2+Stacks, 1660 for Koutajoki bwa+Stacks, 646 for Oulujoki bwa+Stacks) departing from HWE. Population‐wise HWE filtering was applied to remove loci subjected to genotyping errors without reducing the potential to infer population structures (Pearman et al. 2022).

This filtering was performed using the function *gl.filter.hwe* present in *dartR* (Mijangos et al. 2022) package for R v. 4.2.2 (R Core Team 2023). This function filters out loci showing significant departure from HW proportions based on observed frequencies of reference homozygotes, heterozygotes and alternate homozygotes (Mijangos et al. 2022). Moreover, by adding the *seppop* option available in the *adegenet* v. 2.1.10 (Jombart and Ahmed 2011) package for R v. 4.2.2 (R Core Team 2023) we performed a population‐wise filtering that considered SNPs that departed from HWE only within each population. The datasets from the two distinct watersheds (Koutajoki and Oulujoki) were analysed separately for both population structure and outlier loci.

Genome scans were done with two widely used approaches: PCAdapt (v. 4.3.3) (Luu et al. 2017) package for R v. 4.2.2 (R Core Team 2023) and Bayescan (v. 2.1) (Foll and Gaggiotti 2008). An environmental association analysis was also performed by using BayeScEnv (v. 1.1) (de Villemereuil and Gaggiotti 2015). PCAdapt detects outlier loci based on population clustering and has increased statistical power compared to Bayesian models (Bekkevold et al. 2020; Luu et al. 2017). The outliers were detected based on q‐values using a cutoff value of *α =* 0.1 in *qvalue* (v. 2.30.0) (Storey et al. 2015) package for R v. 4.2.2 (R Core Team 2023) and applying a Bonferroni correction for multiple testing. BayeScan identifies candidate loci under natural selection using differences in allele frequencies between populations (Foll and Gaggiotti 2008). The geste file was fed to Bayescan with a burn‐in of 50,000 and 50,000 iterations. BayeScEnv aims at detecting local adaptation linked to a given environmental variable using medium‐ to high‐density genotypic data. It considers the population structure, based on the F‐model, which is the model behind the BayeScan software. BayeScEnv was also run with 2000 iterations and a burn‐in phase of 50,000 iterations. In this case, the environmental variables were relative to the migratory distance of each population assigned as 0.5 for migratory populations and −0.5 for resident populations (Lemopoulos, Uusi‐Heikkilä, Huusko, et al. 2018). In the current study, only 1 out of 3 approaches (PCAdapt) identified loci under selection (see Results). Therefore, to have an additional validation we used PCAdapt only on SNPs shared by the SNP calling pipelines. For the variants detected as outliers, we located them in the DNA, determined its effect, and distance from genes using the annotated genome on the Ensembl Variant Effect Predictor (VEP). In VEP, to identify the genes and transcript close or that overlapped each outlier SNP, we consider a Upstream/Downstream distance from the variant of 5 kb. Therefore, we considered only genes present within a total 10 kb window.

Pairwise and global F_*st*_—values (Weir and Cockerham 1984) and their 95%‐confidence intervals were calculated using *hierfstat v*. 05.11 (Goudet and Jombart 2015) package for R v. 4.2.2 (R Core Team 2023) on the SNP datasets excluding the outlier loci that were detected during the previous genome scan step, as it is likely that the genetic population structure differs between neutral SNPs and those possibly under selection. The Structure analyses were done using the software STRUCTURE v2.3.4 (Pritchard et al. 2000), with a burn‐in of 200,000 and 200,000 repetitions according to Lemopoulos, Uusi‐Heikkilä, Vasemägi, et al. (2018). We set the maximum population number to six for Koutajoki and to four for Oulujoki. The results obtained from the STRUCTURE software were successively fed into CLUMPAK (Kopelman et al. 2015) with CLUMPP LargeGreedy algorithm and 2000 repeats, and dynamic MCL threshold for similarity scores. The best *K* was evaluated using Evanno's Delta *K* method (Evanno et al. 2005) (Supporting Information S1–S3). Ancestry proportion was plotted using *pophelper* server (Francis 2017).

## 3 Results

### 3.1 SNP Detection and Comparison Between Pipelines

**FIGURE 2:**
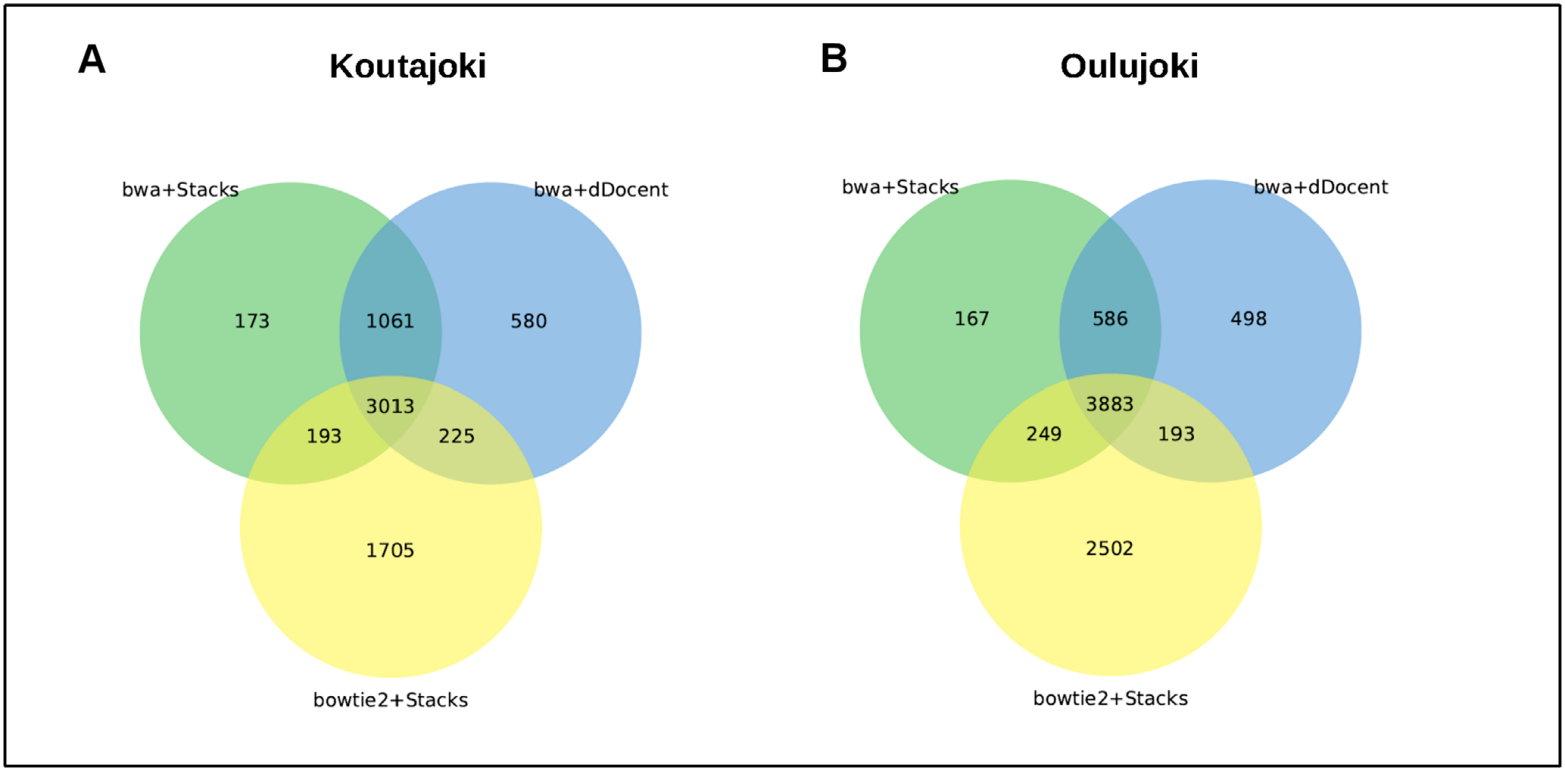
Venn diagrams obtained using vcftoolz and showing the overlaps among the numbers of SNPs obtained after alignment to *Salmo trutta* reference genome and identified using bwa+dDocent (green), bowtie2+Stacks (light blue) and bwa+Stacks (in orange) for Koutajoki (A) and Oulujoki (B) watersheds.

The pipelines identified 4881 and 5164 SNPs for Koutajoki and Oulujoki, respectively using bwa+dDocent, 5236 and 6827 for Koutajoki and Oulujoki, respectively using bowtie2+Stacks and 4441 and 4890 for Koutajoki and Oulujoki, respectively, using bwa+Stacks. After the HWE‐filtering step, 2268 and 4632 SNPs were retained for Koutajoki and Oulujoki, respectively using bwa+dDocent, 3055 and 4500 SNPs using bowtie2+Stacks and 2781 and 4244 using bwa+Stacks. In comparison to Stacks 1.40 used in Lemopoulos, Uusi‐Heikkilä, Huusko, et al. (2018), the number of remaining SNPs was significantly lower (originally 5519 SNPs for Koutajoki and 5670 for Oulujoki). Vcftoolz detected 43% SNPs overlap between the three pipelines for Koutajoki and 48% SNPs overlap between the three pipelines for Oulujoki (Figure 2).

### 3.2 Genetic Structure and Population Differentiation

**FIGURE 3:**
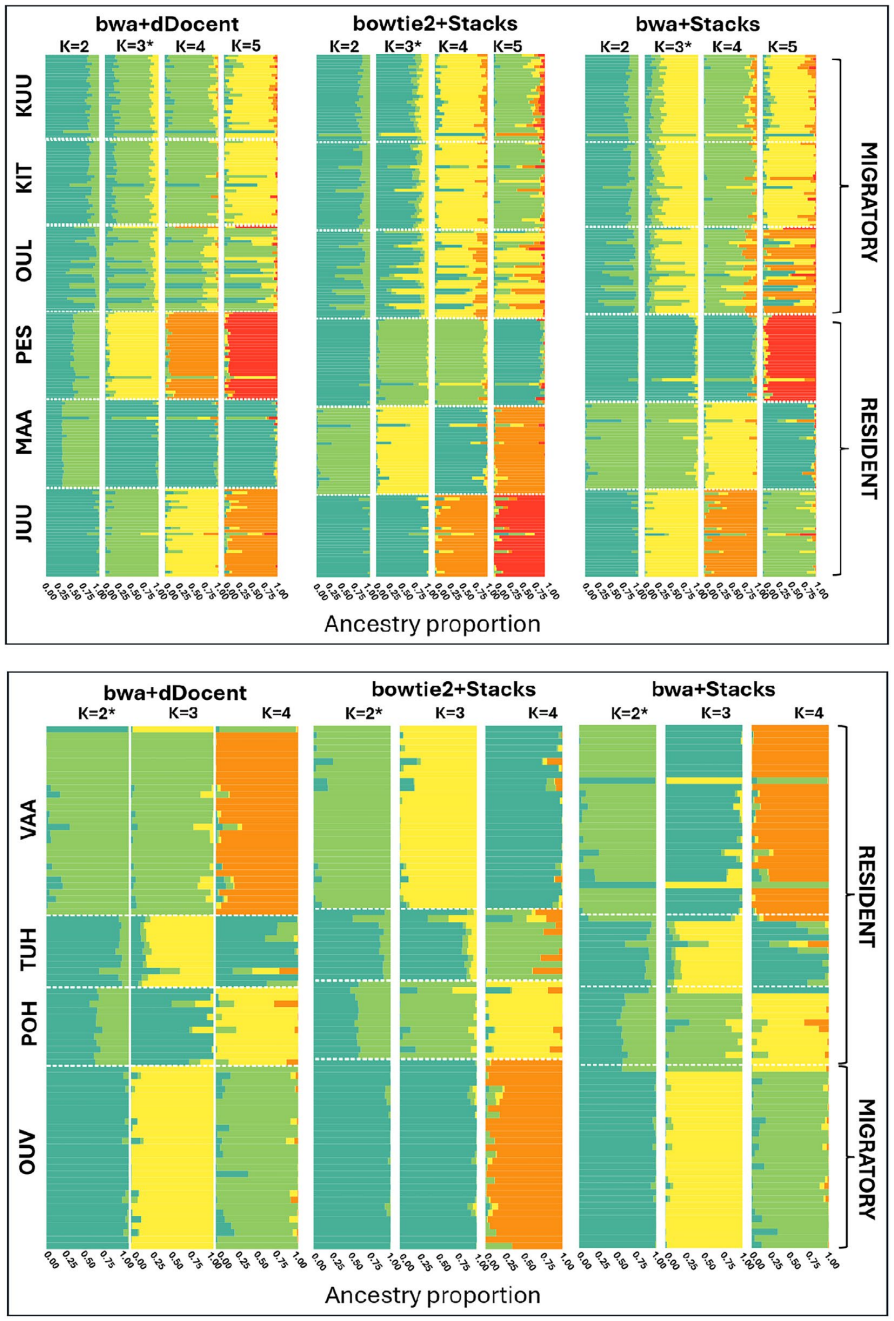
Genetic structure of the studied brown trout populations inhabiting the Koutajoki and Oulujoki watersheds and obtained by running STRUCTURE on bwa+dDocent, bowtie2+Stacks and bwa+Stacks datasets, excluding the outliers. Every column represents a single individual (*N* = 179 and *N* = 80 for Koutajoki and Oulujoki, respectively) and its probability of being assigned to its presumed population. The most probable *K* according to Evanno's delta *K* method is shown with an asterix.

Structure analyses confirmed the original distinction between resident and migratory populations in Koutajoki using both dDocent and Stacks identified SNPs (Supporting Information S1–S3). Different ancestry proportions and relative clusters were initially evident with *K* = 2 for the resident population MAA, that showed a clear divergence from the other resident and migratory populations (Figure 3), especially in the dDocent SNP set. Moreover, with *K* = 3 (the most likely *K* statistically), it was confirmed that the migratory populations were more similar to each other than to the resident populations (Figure 3). *K* = 5 with dDocent further suggested that KIT and KUU cannot be separated, pointing towards common genetic ancestry of those populations (Figure 3). Likewise, for Oulujoki, both datasets obtained from dDocent and Stacks (*K* = 2) (Supporting Information S1–S3) confirmed the documented distinction between the migratory and the resident populations (Figure 3), except for OUV and TUH that seem to share ancestry compared to the other two resident populations.

The divergence of the resident populations from each other and from migratory populations, as well as the lack of divergence among the migratory populations was confirmed by the F_*st*_—values. PES and MAA were the population pair showing the highest divergence (bwa+dDocent‐F_*st*_: 0.294, 95% confidence interval = 0.280–0.308, bowtie2+Stacks‐ F_*st*_: 0.282, 95% confidence interval = 0.282–0.307, bwa+Stacks‐ F_*st*_: 0.310, 95% confidence interval = 0.323–0.296; Tables 2, 3, 4 and Supporting Information S4A–C), followed by MAA and JUU (bwa+dDocent‐F_*st*_: 0.251, 95% confidence interval = 0.237–0.264, bowtie2+Stacks‐F_*st*_: 0.245, 95% confidence interval = 0.234–0.255, bwa+Stacks‐ F_*st*_: 0.259, 95% confidence interval = 0.247–0.270, Tables 2, 3, 4 and Supporting Information S4A–C) and by PES and JUU (bwa+dDocent‐F_*st*_: 0.238, 95% confidence interval = 0.224–0.250, bowtie2+Stacks‐F_*st*_: 0.238, 95% confidence interval = 0.228–0.249, bwa+Stacks‐F_*st*_: 0.246, 95% confidence interval = 0.235–0.258; Tables 2, 3, 4 and Supporting Information S4A–C). The migratory populations, on the other hand, had in general one order of magnitude smaller F_*st*_‐values, with the average F_*st*_ among KIT, OUL and KUU being 0.039 for the three bwa+dDocent, bowtie2+Stacks and bwa+Stacks (Tables 2, 3, 4 and Supporting Information S4A–C).

**TABLE 2:** Pairwise F_*st*_ tables among the Koutajoki (above) and Oulujoki (below) watershed populations and obtained using bwa+dDocent.

Koutajoki bwa+dDocent	JUU	KIT	KUU	MAA	OUL
KIT	0.138				
KUU	0.113	0.016			
MAA	0.251	0.188	0.169		
OUL	0.117	0.058	0.043	0.153	
PES	0.238	0.175	0.163	0.294	0.165

Oulujoki bwa+dDocent	OUV	POH	TUH
POH	0.175		
TUH	0.100	0.202	
VAA	0.155	0.190	0.176

*Note:* The 95%‐confidence intervals can be found in the Supporting Information S4A,D.

**TABLE 3:** Pairwise F_*st*_ tables among the Koutajoki (above) and Oulujoki (below) watershed populations and obtained using bowtie2+Stacks.

Koutajoki bowtie2+Stacks	JUU	KIT	KUU	MAA	OUL
KIT	0.132				
KUU	0.107	0.017			
MAA	0.245	0.180	0.160		
OUL	0.115	0.057	0.043	0.147	
PES	0.238	0.179	0.162	0.294	0.171

Oulujoki bowtie2+Stacks	OUV	POH	TUH
POH	0.198		
TUH	0.110	0.237	
VAA	0.186	0.225	0.211

*Note:* The 95%‐confidence intervals can be found in the Supporting Information S4B,E.

**TABLE 4:** Pairwise F_*st*_ tables among the Koutajoki (above) and Oulujoki (below) watershed populations and obtained using bwa+Stacks.

Koutajoki bwa+Stacks	JUU	KIT	KUU	MAA	OUL
KIT	0.138				
KUU	0.113	0.018			
MAA	0.259	0.192	0.172		
OUL	0.118	0.059	0.045	0.160	
PES	0.246	0.180	0.168	0.310	0.173

Oulujoki bwa+Stacks	OUV	POH	TUH
POH	0.203		
TUH	0.115	0.243	
VAA	0.178	0.219	0.204

*Note:* The 95%‐confidence intervals can be found in the Supporting Information S4C,F.

Similar trends were shown by the F_*st*_‐values calculated on both dDocent and Stacks datasets for Oulujoki, with POH and TUH having the highest F_*st*_ (bwa+dDocent‐F_*st*_: 0.202, 95% confidence interval = 0.193–0.210, bowtie2+Stacks‐F_*st*_: 0.237, 95% confidence interval = 0.227–0.247, bwa+Stacks‐F_*st*_: 0.243, 95% confidence interval = 0.234–0.253; Tables 2, 3, 4 and Supporting Information S4D–F) and OUV and VAA having a lower F_*st*_ in two pipelines (dDocent‐F_*st*_: 0.155, 95% confidence interval = 0.149–0.161, Stacks‐F_*st*_: 0.186, 95% confidence interval = 0.180–0.193, bwa+Stacks‐F_*st*_: 0.178, 95% confidence interval = 0.171–0.185; Tables 2, 3, 4 and Supporting Information S4D–F). The lowest F_*st*_‐value was found between the migratory population OUV and the resident population TUH (dDocent‐F_*st*_: 0.100, 95% confidence interval = 0.094–0.105; Stacks‐F_*st*_: 0.110, 95% confidence interval = 0.104–0.115, bwa+Stacks‐F_*st*_: 0.115, 95% confidence interval = 0.110–0.121; Tables 2, 3, 4 and Supporting Information S4D–F).

### 3.3 Loci Under Selection

**FIGURE 4:**
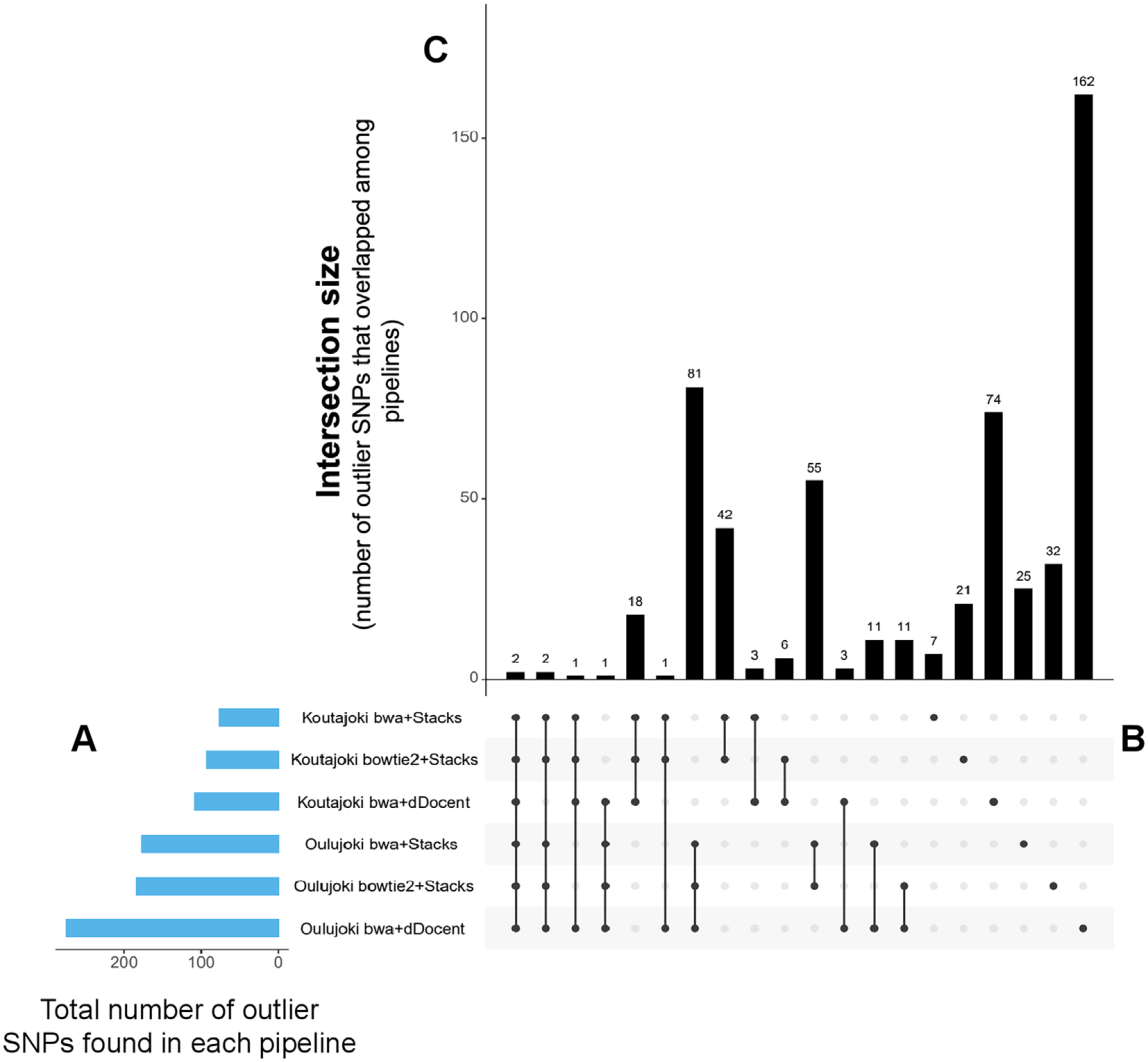
Plot showing the frequency of outlier SNPs that overlapped among different pipelines and watersheds: (A) the horizontal blue bars on the left‐hand side represent the number of outlier SNPs found in each dataset using PCAdapt. (B) The vertical connection filled with dark circles represents the pipeline intersection sets. (C) The top vertical bar charts represent the intersection size, i.e., how many outlier SNPs are shared among different pipelines.

Only PCAdapt identified loci under selection by suggesting 110, 95 and 77 SNPs loci under selection in bwa+dDocent, bowtie2+Stacks and bwa+Stacks SNP sets, respectively, in Koutajoki. In Oulujoki, PCAdapt identified 282, 189 and 177 loci under selection in bwa+dDocent, bowtie2+Stacks and bwa+Stacks SNP sets, respectively (Supplementary S5 and S6). Two of the loci showed general importance by being shared with all the SNP subsets (Figure 4), while other two loci were shared with five out of six datasets and four out of six datasets (Figure 4). Hundred loci, on the other hand, were shared by three SNP subsets (Figure 4). After inspection using VEP, we found that eight of the loci shared at least in 3 datasets were within or very close to candidate genes found to be associated with migratory tendency in previous studies (Table 5). All the rest of the outliers are presented in Supporting Information S7.

**TABLE 5:** List of candidate genes that have been found in 6 (bwa+dDocent Koutajoki, bwa+dDocent Oulujoki, bowtie2+Stacks Koutajoki and bowtie2+Stacks Olulujoki, bwa+Stacks Koutajoki and bwa+Stacks Oulujoki), 5 datasets (bwa+dDocent River Koutajoki, bwa+dDocent River Oulujoki, bowtie2+Stacks River Koutajoki and bowtie2+ Stacks River Olulujoki, bwa+Stacks Oulujoki) or 4 (bwa+dDocent Oulujoki, bowtie2+Stacks Koutajoki, bowtie2+Stacks Olulujoki and bwa+Stacks Oulujoki) and/or had previously observed to be related to migratory or residency behaviour, and their distances to the outlier SNPs that were detected by both dDocent and Stacks 2.65 pipelines.

SNP ID	Location (CHR:POS)	Dataset occurrence	Distance from gene	Gene symbol	Gene name	Function	Previous studies	Species
1_57,386,539_A/C	1:57386539	4	2956	ST6GALNAC2	ST6 N‐acetylgalactosaminide alpha‐2,6‐sialyltransferase 2	Mucus production in anadromous brown trout	Malachowicz et al. 2017; Lemopoulos, Uusi‐Heikkilä, Huusko, et al. 2018	*Salmo trutta m. trutta*
3_9,902,677_C/T	3:9902677	6	0	PRDM9‐like	Histone‐lysine N‐methyltransferase PRDM9‐like	Driver of meiotic recombination hotspot in salmonids	Raynaud et al. 2025	*Onchoryncus mykiss, Salmo salar*
3_9,902,717_T/C	3:9902717	6	0	PRDM9‐like	Histone‐lysine N‐methyltransferase PRDM9‐like	Driver of meiotic recombination hotspot in salmonids	Raynaud et al. 2025	*Onchoryncus mykiss, Salmo salar*
9_16,268,531_C/T	9:16268531	4	0	TMEM132D	Transmembrane protein 132D‐like	Migratory behaviour	Tigano and Russello 2022	*Onchoryncus nerka*
13_17,025,604_C/T	13:17025604	4	0	fbxw11a	F‐box/WD repeat‐containing protein 7‐like	Migratory behaviour	Tigano and Russello 2022	*Onchoryncus nerka*
16_20,774,328_G/A	16:20774328	4	0	arl8a	ADP‐ribosylation factor‐like protein 15	Migratory behaviour	Hale et al. 2013	*Onchoryncus mykiss*
16_20,774,328_G/A	16:20774328	4	0	znf76	Zinc finger protein 76	Smoltification	Lemopoulos, Uusi‐Heikkilä, Huusko, et al. 2018	*Salmo trutta* , *Salmo salar*
16_59,376,134_G/A	16:59376134	5	0	PTPRT	Protein tyrosine phosphatase receptor type T	Unknown in salmonids or other fish	—	*—*
21_282,471_G/A	21:282471	6	0	asap1a	ArfGAP with SH3 domain, ankyrin repeat and PH domain 1a	Role in processes relevant to membrane/cytoskeleton	—	*—*
25_33,689,731_C/A	25:33689731	4	689	CLDN20	Claudin‐20‐like	Freshwater vs marine adaptation	Ferchaud et al. 2014; Wang et al. 2020	*Gasterosteus aculeatus* , *Pungitius pungitius*
30_27,536,599_C/A	30:27536599	4	0	nup210	Nuclear pore membrane glycoprotein 210‐like	Migratory behaviour	Tigano and Russello 2022	*Onchoryncus nerka*
38_6,437,497_C/T	38:6437497	4	7296	KCNJ4	Potassium inwardly rectifying channel subfamily J member 4	Migratory behaviour and adaptation to saltmarsh microhabitats	Tigano and Russello 2022; Wagner et al. 2017; Moran et al. 2024	*Onchoryncus nerka*, *Fundulus heteroclitus* , *Salmo trutta*

*Note:* Since the SNP “16_20,774,328_G/A” was found to be really close to two genes, we listed it twice in the table.

## 4 Discussion

The choice of software for SNP calling had a strong effect on both the number and identity of the detected SNPs, and respectively on the outlier detection results. The new conspecific sequence alignment did not affect the original inferences made on the population structure of the sampled fish (Lemopoulos, Uusi‐Heikkilä, Vasemägi, et al. 2018). However, compared to Lemopoulos, Uusi‐Heikkilä, Huusko, et al. (2018), the alignment of sequences on the brown trout reference genome helped to identify new loci that might associate with migratory dichotomy in brown trout. Two of the candidate SNPs were located within a candidate gene region as identified using the salmon reference genome (Lemopoulos, Uusi‐Heikkilä, Huusko, et al. 2018). Therefore, our results overall are confirmatory and show that technical choices do matter for the outlier detection (see also Manel et al. 2016; Shafer et al. 2017) while for basic population genetics, a set of some thousands of neutral SNPs is enough independently of the technical choices.

The two alternative SNP identification methods yielded different numbers of SNPs, with Stacks generally identifying more SNPs than dDocent when used in combination with bowtie2, for both study systems. This is not surprising due to the inherent differences in the algorithms of these pipelines (Liu et al. 2022; Torkamaneh et al. 2016). dDocent uses Burrows‐Wheeler Alignment algorithm (*bwa*) (Li and Durbin 2009) for the reference genome alignment. It also detects structural variants (indels) other than SNPs and is therefore particularly suitable for long reads (our average read length was 85 bp after barcode trimming). Stacks offers more flexibility when choosing alignment tools, allowing both *bwa* or *bowtie2*. In our study it seems that the combination bowtie2+Stacks resulted in the highest number of SNPs, while using bwa seems a bit more conservative for both the pipelines. Our results showing a different SNP calling success between these pipelines are in line with previous studies (Puritz et al. 2014). However, only about 45% of SNPs were shared between the pipelines, which further highlights the differences between them. In the present study, downstream analyses of population structure were done using the complete filtered SNP dataset from each pipeline, rather than only the SNPs detected by both, which were used for outlier detection for robustness. Nonetheless, the inferred population structure and divergence in both watersheds were consistent across the datasets from both pipelines. This suggests that, although the pipelines identified different sets of SNPs, both produced enough SNPs to infer population structure and divergence, and as such there may not be a need to compare the analysis pipelines for basic population or landscape genetics (c.f. Shafer et al. 2017). However, the higher number of SNPs obtained with Stacks is reflected in higher statistical power in downstream analyses.

Heterospecific reference genomes should reduce alignment success. For example, by aligning RADseq data from the coho salmon ( *Oncorhynchus kisutch* ) to six different salmonid reference genomes, including the one of coho salmon itself, Bohling (2020) observed that increasing phylogenetic distance between the target species and the reference genome reduced read alignment success and in turn the number of SNPs detected. Although the Atlantic salmon has been considered phylogenetically extremely close to the brown trout (Leitwein et al. 2017), these species show differences also in chromosomal arrangements (Leitwein et al. 2017). Against expectations, the use of the brown trout reference genome here resulted in a lower number of SNPs, after filtering, compared to the original study (Lemopoulos, Uusi‐Heikkilä, Huusko, et al. 2018) with the Atlantic salmon reference genome. However, this result is confounded by the different SNP calling procedures, as Lemopoulos, Uusi‐Heikkilä, Huusko, et al. (2018) used the older Stacks v1. The latest version implements a Bayesian genotype caller (BGC), meaning that SNP genotyping relies on a Bayesian prior before being defined. This procedure might explain the discrepancy. The *S. trutta* reference genome should provide significantly better contiguity (N50 = 52.21 Mb in *S. trutta* vs. 17 Mb in *S. salar* ) and structural quality (fewer scaffolds in *S. trutta* compared to *S. salar* ), with fewer gaps compared to *S. salar* according to the assembly statistics for each genome. Consistent with our results, Shafer et al. (2017), used ddRADseq data from the Galápagos sea lion ( *Zalophus wollebaeki* ), and showed that the number of SNPs per locus generally increased as the reference genome became more distantly related. In their study, Shafer et al. (2017) observed that more distant genomes led to low Ts/Tv (transition/transversion) ratio, which suggests errors in variant calling, further recommending avoiding alignment on distantly related genomes, but instead to use phylogenetically as close references as possible. Thus, the discrepancy in the number of SNPs obtained here and in Lemopoulos, Uusi‐Heikkilä, Huusko, et al. (2018) is likely explained partially by erroneous original fragment alignment and partially by improved Bayesian SNP calling algorithm in the new version of Stacks. Filtering steps between the previous study with alignment on *S. salar* reference and the present study were the same for Stacks v.2 and followed similar filtering criteria despite the usage of different filtering software for dDocent. This suggests that the SNP yield could be influenced by technical choices, and close phylogenetic match should in theory always produce more SNPs and less artefacts. In the absence of a conspecific genome, Atlantic salmon genome was the best possible reference for brown trout in Lemopoulos, Uusi‐Heikkilä, Huusko, et al. (2018), and our results here confirm that the choice of the reference genome did not have any relevant influence on the inferred population structure among the studied fish.

In both River Koutajoki and Oulujoki, resident populations were genetically distinct from each other and from the migratory main stem populations, supporting the conclusion of reproductive isolation driven by geographic and behavioural factors (Lemopoulos, Uusi‐Heikkilä, Vasemägi, et al. 2018). In contrast, fish from the main stems, i.e., the migratory stocks, showed higher admixture, as seen in the Structure analyses (Figure 3). The F‐statistics confirmed these patterns across pipelines, with SNPs identified by Stacks producing generally higher F_*st*_‐values than SNPs from dDocent. The clearest difference between the current analyses and those of Lemopoulos, Uusi‐Heikkilä, Vasemägi, et al. (2018) was for the River Tuhkajoki (TUH) population that here had similar ancestry proportion as the hatchery stock suggesting potential stocking‐origin that was not detected in the original analyses. Similar cases of landscape‐driven, as well as a potential behavioural or human induced, population isolation have been commonly observed for brown trout systems (Carlsson and Nilsson 2001; Lagunas et al. 2023). Non‐migratory behaviour may reflect local adaptation and drift following reproductive isolation of formerly migratory founders that have colonised the current ranges after glacial recession (García Marín et al. 1999; Huusko et al. 2017; Hynes et al. 1996). Such a process could occur rather independently in each headwater population and explain why universal migratory genes have not been found. Yet, parallel evolution affecting common genetic regions is also possible (Miller et al. 2012; Pearse et al. 2014; Waples et al. 2004). On the other hand, we did not have individual movement data for all the sampled fish, and some tributaries may have included hatchery‐produced migratory individuals with confounding effects on the analyses.

Aligning RAD tags to the *S. trutta* reference genome yielded novel outlier SNPs compared to the original study with alignment to the *S. salar* genome (Lemopoulos, Uusi‐Heikkilä, Huusko, et al. 2018). Nonetheless, only one approach out of three yielded outliers in both study systems. Why only PCAdapt produced statistically significant outliers may relate to the more conservative approaches used in Bayescan and BayeScEnv (Luu et al. 2017). Moreover, PCAdapt has been shown to be among the most powerful approaches to detect trait‐associated genetic variation in the presence of highly divergent populations like in this study, while Bayescan has been shown to perform poorly when population divergence is high. Bayescan performance has been shown to be highly context‐dependent (de Villemereuil and Gaggiotti 2015), especially in strongly hierarchical scenarios with confounding effects between demography and environmental variables. Therefore, its power could be weak, and the error rate could be high. This might be the reason why this approach was not successful in our study context and further highlight the importance of using more than one approach when searching for signals of adaptive divergence. It is also possible that evolutionary processes such as hitchhiking cause differences in detection of loci under selection with different approaches (e.g., Stephan 2019). Bekkevold et al. (2020) similarly showed contrasting results among genome scan approaches in brown trout. Since only one approach (PCAdapt) produced significant outlier loci, we examined only the outliers shared by the three pipelines and the two watersheds for robustness. By locating the outliers on the reference genome, they were found to be spread throughout the whole genome without evidence of blocks of highly divergent loci within few or small genomic regions (Supporting Information S6 and S7). Notably, among these outliers, 8 were found close to genes that have been identified also in previous studies of migration propensity. One SNP on chromosome 1 was found to be 2956 bp from the gene ST6GALNAC2 (ST6 N‐acetylgalactosaminide alpha‐2,6‐sialyltransferase 2) that is involved in mucus production in brown trout (Malachowicz et al. 2017) and that was identified also in the original study of Lemopoulos, Uusi‐Heikkilä, Huusko, et al. (2018). As fish often encounter a wide variety of microbiota, especially when migrating from one place to another, their immune system needs to differentiate between commensal and pathogenic microbiota to avoid excessive inflammatory responses (McMurtrie et al. 2025). Mucus on the skin and gills might host different microorganisms that contribute to immune defence and healthy skin microbiota, an important function when habitat changes expose fish to potentially new pathogens. Thus, this candidate gene suggests that mucus production plays a role in adaptation to migratory life‐style, warranting further investigation. Another locus was found within the zinc finger protein 76 gene (znf76) confirming results of Lemopoulos, Uusi‐Heikkilä, Huusko, et al. (2018). Zinc‐finger proteins belong to the wide group of proteins involved in several molecular functions (Cassandri et al. 2017). In salmonids, these class of proteins include immune functions during smoltification by being expressed in liver (Harvey et al. 2024) and gills (Seear et al. 2010) of anadromous Atlantic salmon and in coho salmon (Gallagher et al. 2008), suggesting a similar function in brown trout. Another SNP was found about ~7 Kbp from *KCNJ4* (potassium inwardly rectifying channel subfamily J member 4), also suggested to be involved in migratory behaviour in brown trout (Moran et al. 2024) and other salmonids (Tigano and Russello 2022; Wagner et al. 2017) in relation to processes of acclimatisation to different salinity gradients in many other fishes including salmonids (Gideon et al. 2025; Hale et al. 2013). In our study, migratory brown trout were adfluvial without the need to tolerate saltwater. However, genetic variation tied to osmoregulation indicates that adfluvial fish inherit linked traits that support migration, including their ancestral ability to tolerate salinity gradients (Arostegui et al. 2019; Piironen et al. 2013; Vainikka et al. 2023). Other outliers were found nearby or within genes that have been previously considered as candidate genes involved in freshwater vs. marine adaptation, again supporting the hypothesis that ancestral capacity to acclimate to different environmental conditions typical of the anadromy have been conserved also in adfluvial populations. Among those we identified *CLDN20*, detected in several studies (Chen and Narum 2021; Ferchaud et al. 2014; Tian et al. 2023; Wang et al. 2020). Some SNPs were found near gene regions previously observed to be involved in migratory behaviour in salmonids (Hale et al. 2013; Tigano and Russello 2022); among those genes we identified *arl8a*, *fbxw11a*, *nup210* and *TMEM132D*. Interestingly, two outliers were found in all the SNP subsets and both within the same gene PRDM9‐like (histone‐lysine N‐methyltransferase PRDM9‐like), which is involved in meiotic recombination in many species and which was recently discovered in salmonids (Raynaud et al. 2025). Other outliers found in all four datasets were within PTPRT (protein tyrosine phosphatase receptor type T) and asap1a (ArfGAP with SH3 domain, ankyrin repeat and PH domain 1a), whose role in salmonids or other fish is not known. Overall, the fact that several SNPs were in proximity to genes previously found to be involved in migratory/residence behaviour, osmoregulation and growth, suggests that these genes have a role in explaining also the migration dichotomy in the study populations addressed here. In *Onchorhynchus mykiss*, a large chromosomal inversion has been linked to migratory behaviour (Arostegui et al. 2019; Clare et al. 2023), which suggests that a similar pattern in *S. trutta* could not be totally excluded, yet most studies for now generally indicate that variation in migration tendency is shaped by many genes with small effects and that no specific genomic region seems to explain the phenotypic variation.

In conclusion, population structure and divergence patterns remained consistent with the original study (Lemopoulos, Uusi‐Heikkilä, Vasemägi, et al. 2018) yet the re‐analysis yielded fewer selectively neutral SNPs. SNP detection rate varied between pipelines and reference genomes, highlighting the impact of different bioinformatic methods and reference genomes on SNP detection and further downstream inferences. Different outlier analyses revealed a limited overlap among the genome scan approaches, compared to Lemopoulos, Uusi‐Heikkilä, Huusko, et al. (2018), stressing a need for caution when inferring targets of selection. However, we identified several SNPs with known associations to migration, growth, and other biologically relevant traits, suggesting that migratory tendency in brown trout eventually has a multigenic architecture. Since the re‐analysis performed here did not improve the original coverage of the RAD‐sequencing approach, the genomic architecture of migration still awaits new whole‐genome approaches.

## Author Contributions

**Giovanna Mottola:** data curation (equal), formal analysis (equal), investigation (equal), software (equal), visualization (equal), writing – original draft (equal). **Frank Panitz:** formal analysis (equal), writing – review and editing (equal). **Tuomas Leinonen:** funding acquisition (equal), writing – review and editing (equal). **Alexandre Lemopoulos:** writing – review and editing (equal). **Anssi Vainikka:** conceptualization (equal), funding acquisition (equal), project administration (equal), supervision (equal), writing – review and editing (equal).

## Funding

This work was funded by Research Council of Finland grant #347367.

## Conflicts of Interest

The authors declare no conflicts of interest.

## Supporting information


**Supporting Information: S1.** ΔK statistics, calculated using Evanno's method, applied to determine the most likely number of genetic clusters (*K*) within River Koutajoki (graphs above) and Oulujoki (graphs below), using SNPs obtained by running bwa+dDocent pipeline after analyzing them using STRUCTURE software.
**Supporting Information: S2** Δ*K* statistics, calculated using Evanno's method, applied to determine the most likely number of genetic clusters (*K*) within the River Koutajoki and Oulujoki, using SNPs obtained by running bowtie2+Stacks pipeline after analyzing them using STRUCTURE software.
**Supporting Information: S3** ΔK statistics, calculated using Evanno's method, applied to determine the most likely number of genetic clusters (*K*) within the River Koutajoki and Oulujoki, using SNPs obtained by running bwa+Stacks pipeline after analyzing them using STRUCTURE software.
**Supporting Information: S4** *F*_st_ 95%‐confidence intervals for the pairwise *F*_st_ (lower 95% CIs on the lower diagonal, upper 95% CIs on the upper diagonal).
**Supporting Information: S5** Venn Diagrams showing the intersection among three different genome scan approaches (PCAdapt, Bayescan, BayeScEnv) to detect loci under selection using SNPs obtained from bwa+dDocent (in green), bowtie2+Stacks (in blue) and bwa+Stacks (in orange).
**Supporting Information: S6** Manhattan plot showing the distribution of the SNPs found in each dataset and watershed by chromosome (x‐axis) and the log‐transformation of the *p*‐values (−log_10_(*p*)) on the *y*‐axis.
**Supporting Information: S7** List of the all the outliers overlapping between pipelines (bwa+dDocent, bowtie2+Stacks and bwa+Stacks) and watersheds (Koutajoki and Oulujoki), that did not show any known association with migration/residency.

## Data Availability

All the data and codes used in the current manuscript are stored in Dryad and are publicly available at https://doi.org/10.5061/dryad.v6wwpzh9f.
