# Supplementary material for: Effects of Different SNP Calling and Sequence Mapping Choices on the Inference of Genetic Architecture Underlying Migration Tendency
Source: Ecol Evol. 2026 May 27;16(6):e73743. doi: 10.1002/ece3.73743 (PMC13240246; doi:10.1002/ece3.73743)
Supplement: Supplementary file 1 — Supporting Information: S1. ΔK statistics, calculated using Evanno's method, applied to determine the most likely number of genetic clusters (K) within River Koutajoki (graphs above) and Oulujoki (graphs below), using SNPs obtained by running bwa+dDocent pipeline after analyzing them using STRUCTURE software. Supporting Information: S2 ΔK statistics, calculated using Evanno's method, applied to determine the most likely number of genetic clusters (K) within the River Koutajoki and Oulujoki, using SNPs obtained by running bowtie2+Stacks pipeline after analyzing them using STRUCTURE software. Supporting Information: S3 ΔK statistics, calculated using Evanno's method, applied to determine the most likely number of genetic clusters (K) within the River Koutajoki and Oulujoki, using SNPs obtained by running bwa+Stacks pipeline after analyzing them using STRUCTURE software. Supporting Information: S4 F st 95%‐confidence intervals for the pairwise F st (lower 95% CIs on the lower diagonal, upper 95% CIs on the upper diagonal). Supporting Information: S5 Venn Diagrams showing the intersection among three different genome scan approaches (PCAdapt, Bayescan, BayeScEnv) to detect loci under selection using SNPs obtained from bwa+dDocent (in green), bowtie2+Stacks (in blue) and bwa+Stacks (in orange). Supporting Information: S6 Manhattan plot showing the distribution of the SNPs found in each dataset and watershed by chromosome (x‐axis) and the log‐transformation of the p‐values (−log10(p)) on the y‐axis. Supporting Information: S7 List of the all the outliers overlapping between pipelines (bwa+dDocent, bowtie2+Stacks and bwa+Stacks) and watersheds (Koutajoki and Oulujoki), that did not show any known association with migration/residency. [file ECE3-16-e73743-s001.docx]

**Supplementary material**

**Effects of different SNP calling and sequence mapping choices on the inference of genetic architecture underlying migration tendency**

Giovanna Mottola^1,2,*^, Frank Panitz^3^, Tuomas Leinonen^4^, Alexandre Lemopoulos^1^, Anssi Vainikka^1^

1. Department of Environmental and Biological Sciences, University of Eastern Finland, FIN-80101 Joensuu, Finland
2. Genomics and Breeding Unit (GEJA), Natural Resources Institute Finland (LUKE), Kulttuurikuja 1, FIN-20800 Turku, Finland
3. Natural Resources Unit (LUVA), Natural Resources Institute Finland (LUKE), Kulttuurikuja 1, FIN-20800 Turku, Finland
4. Genomics and Breeding Unit (GEJA), Natural Resources Institute Finland (LUKE), Latokartanonkaari 9, FIN-00790 Helsinki, Finland

*corresponding author [giovanna.mottola@uef.fi](mailto:giovanna.mottola@uef.fi)


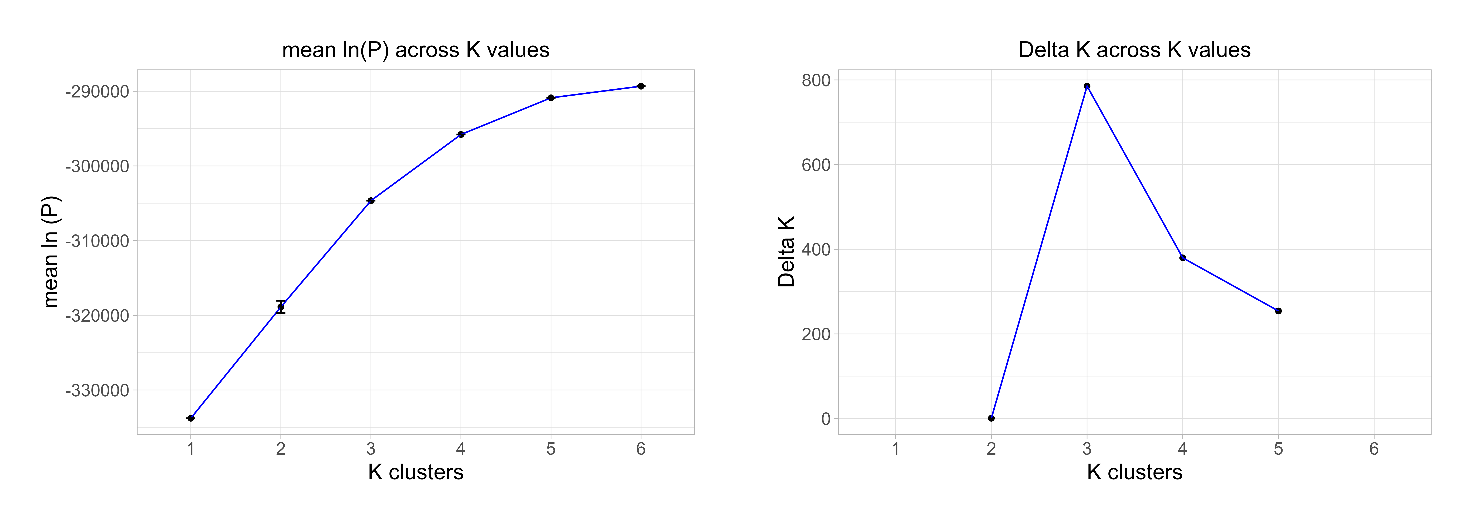


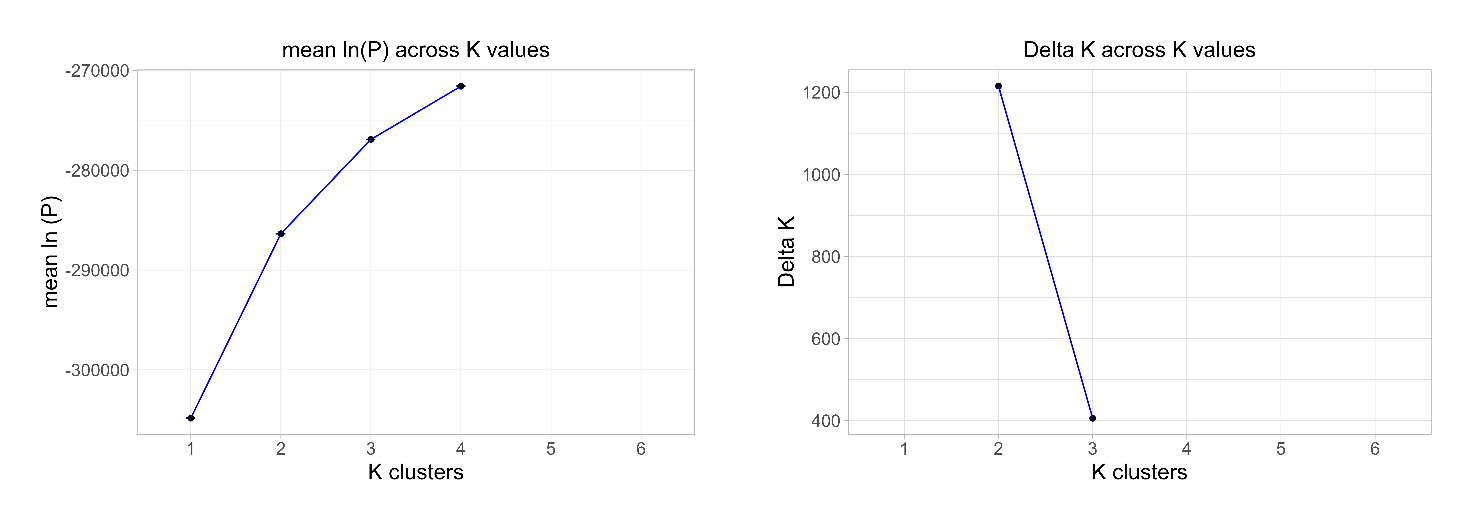


**S1.** ΔK statistics, calculated using Evanno’s method, applied to determine the most likely number of genetic clusters (K) within River Koutajoki (graphs above) and Oulujoki (graphs below), using SNPs obtained by running bwa+dDocent pipeline after analyzing them using STRUCTURE software.


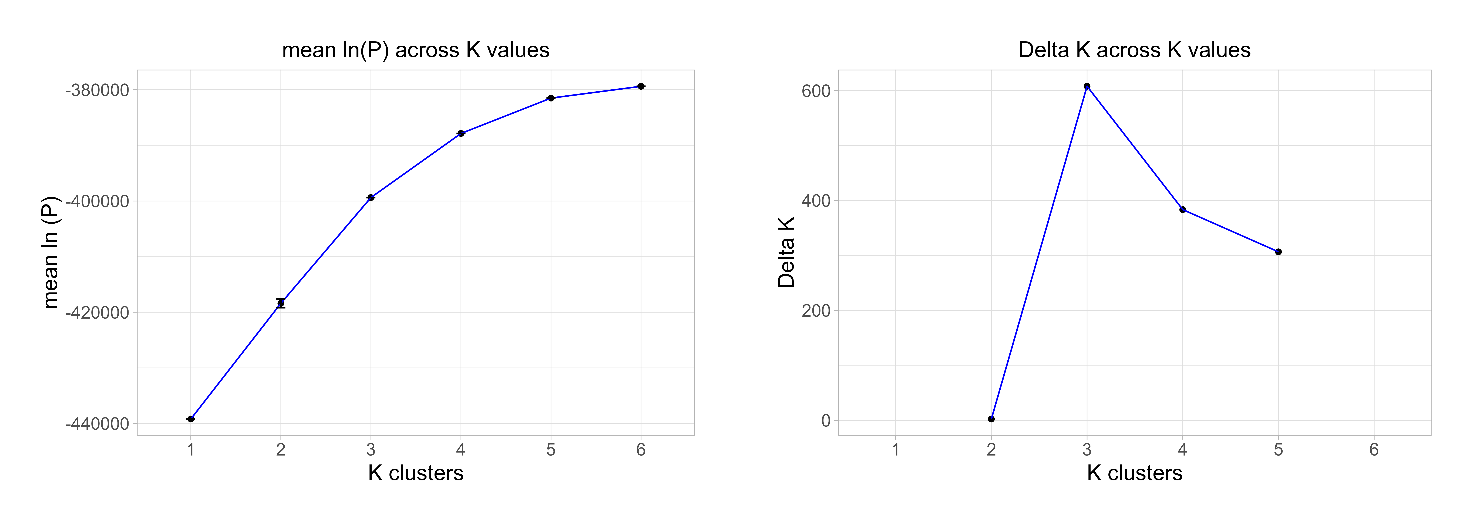


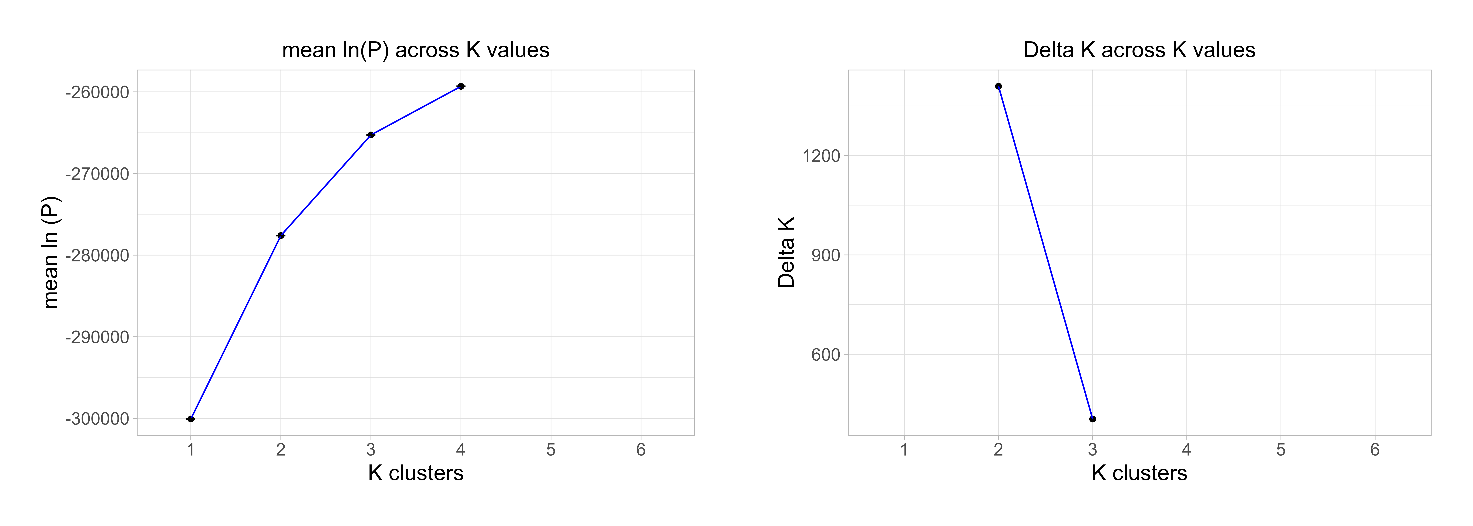


**S2.** ΔK statistics, calculated using Evanno’s method, applied to determine the most likely number of genetic clusters (K) within the River Koutajoki and Oulujoki, using SNPs obtained by running bowtie2+Stacks pipeline after analyzing them using STRUCTURE software.


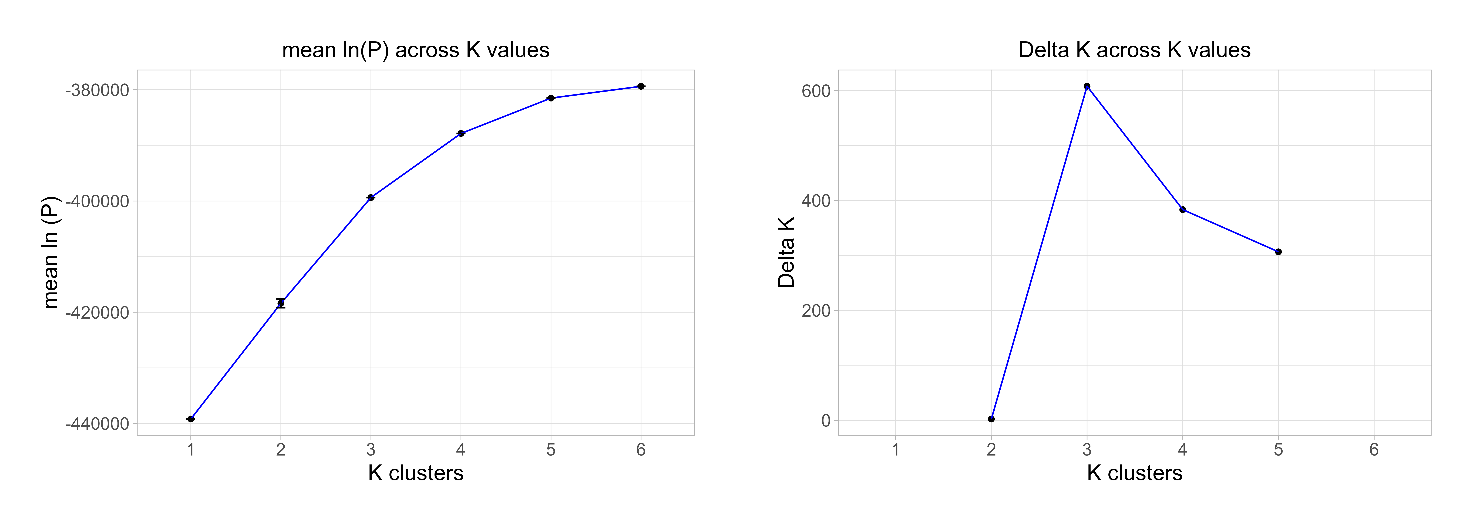


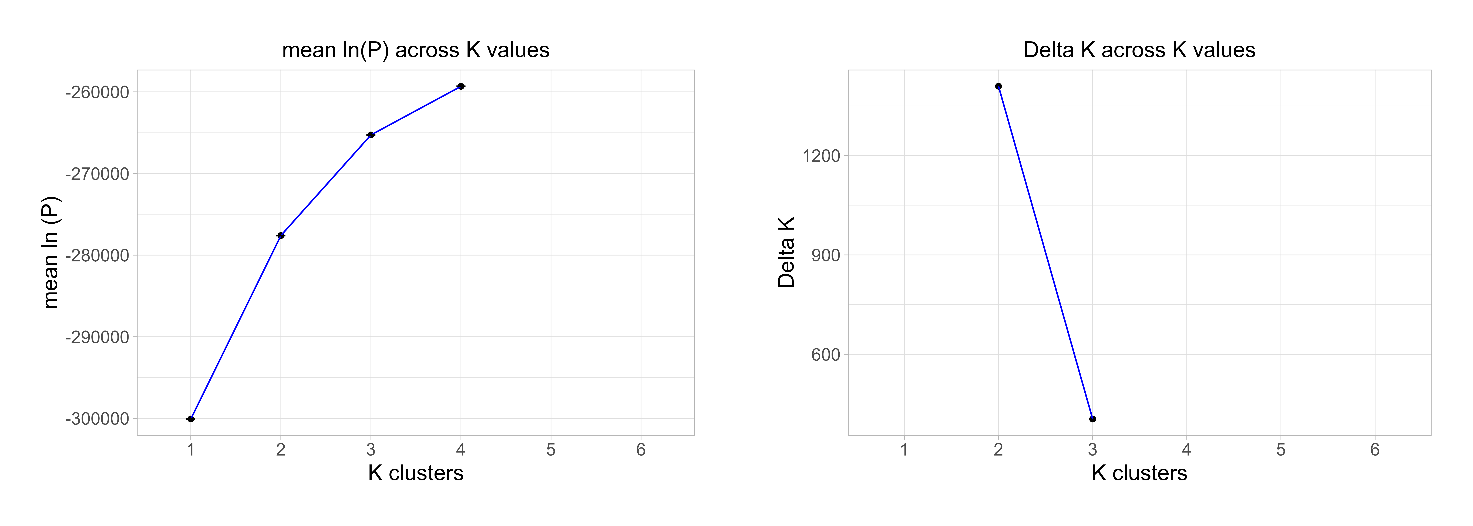


**S3.** ΔK statistics, calculated using Evanno’s method, applied to determine the most likely number of genetic clusters (K) within the River Koutajoki and Oulujoki, using SNPs obtained by running bwa+Stacks pipeline after analyzing them using STRUCTURE software.

**S4.** F*_st_* 95%-confidence intervals for the pairwise F*_st_* (lower 95% CIs on the lower diagonal, upper 95% CIs on the upper diagonal).

**S4A. Koutajoki bwa+dDocent 95% CI (lower and higher limits)**

|  | JUU | KIT | KUU | MAA | OUL | PES |
| --- | --- | --- | --- | --- | --- | --- |
| JUU |  | 0.147 | 0.120 | 0.264 | 0.125 | 0.250 |
| KIT | 0.130 |  | 0.018 | 0.199 | 0.063 | 0.186 |
| KUU | 0.105 | 0.013 |  | 0.179 | 0.046 | 0.174 |
| MAA | 0.238 | 0.178 | 0.159 |  | 0.161 | 0.309 |
| OUL | 0.110 | 0.054 | 0.039 | 0.145 |  | 0.175 |
| PES | 0.225 | 0.164 | 0.153 | 0.280 | 0.155 |  |

**S4B. Koutajoki bowtie2+Stacks 95%-CI (lower and higher limits)**

|  | JUU | MAA | PES | OUL | KIT | KUU |
| --- | --- | --- | --- | --- | --- | --- |
| JUU |  | 0.256 | 0.250 | 0.122 | 0.140 | 0.113 |
| MAA | 0.234 |  | 0.307 | 0.155 | 0.189 | 0.169 |
| PES | 0.228 | 0.283 |  | 0.181 | 0.189 | 0.171 |
| OUL | 0.109 | 0.141 | 0.164 |  | 0.061 | 0.046 |
| KIT | 0.125 | 0.172 | 0.171 | 0.054 |  | 0.019 |
| KUU | 0.101 | 0.153 | 0.155 | 0.039 | 0.015 |  |

**S4C. Koutajoki bwa+Stacks 95%-CI (lower and higher limits)**

|  | JUU | MAA | PES | OUL | KIT | KUU |
| --- | --- | --- | --- | --- | --- | --- |
| JUU |  | 0.146 | 0.119 | 0.270 | 0.125 | 0.258 |
| MAA | 0.130 |  | 0.020 | 0.202 | 0.064 | 0.190 |
| PES | 0.106 | 0.016 |  | 0.180 | 0.048 | 0.176 |
| OUL | 0.247 | 0.182 | 0.163 |  | 0.168 | 0.323 |
| KIT | 0.111 | 0.055 | 0.041 | 0.152 |  | 0.181 |
| KUU | 0.235 | 0.171 | 0.159 | 0.296 | 0.165 |  |

**S4D. Oulujoki bwa+dDocent 95%-CI (lower and higher limits)**

|  | OUV | POH | TUH | VAA |
| --- | --- | --- | --- | --- |
| OUV |  | 0.183 | 0.105 | 0.162 |
| POH | 0.168 |  | 0.211 | 0.198 |
| TUH | 0.094 | 0.193 |  | 0.183 |
| VAA | 0.150 | 0.182 | 0.168 |  |

**S4E. Oulujoki bowtie2+Stacks 95%-CI (lower and higher limits)**

|  | OUV | POH | TUH | VAA |
| --- | --- | --- | --- | --- |
| OUV |  | 0.207 | 0.116 | 0.193 |
| POH | 0.190 |  | 0.247 | 0.234 |
| TUH | 0.105 | 0.228 |  | 0.219 |
| VAA | 0.180 | 0.215 | 0.203 |  |

**S4F. Oulujoki bwa+Stacks 95%-CI (lower and higher limits)**

|  | OUV | POH | TUH | VAA |
| --- | --- | --- | --- | --- |
| OUV |  | 0.185 | 0.227 | 0.213 |
| POH | 0.171 |  | 0.211 | 0.121 |
| TUH | 0.209 | 0.195 |  | 0.253 |
| VAA | 0.196 | 0.110 | 0.234 |  |


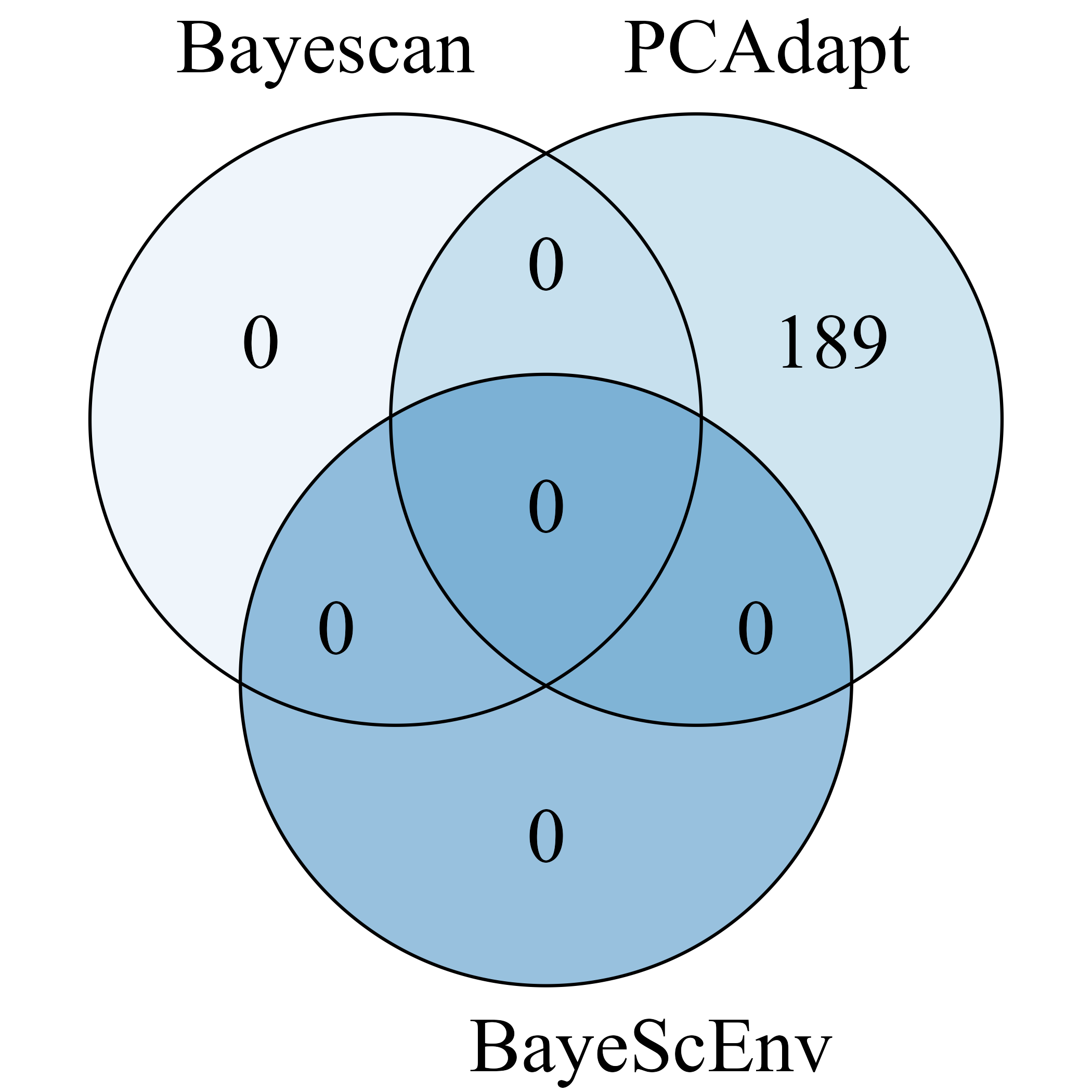

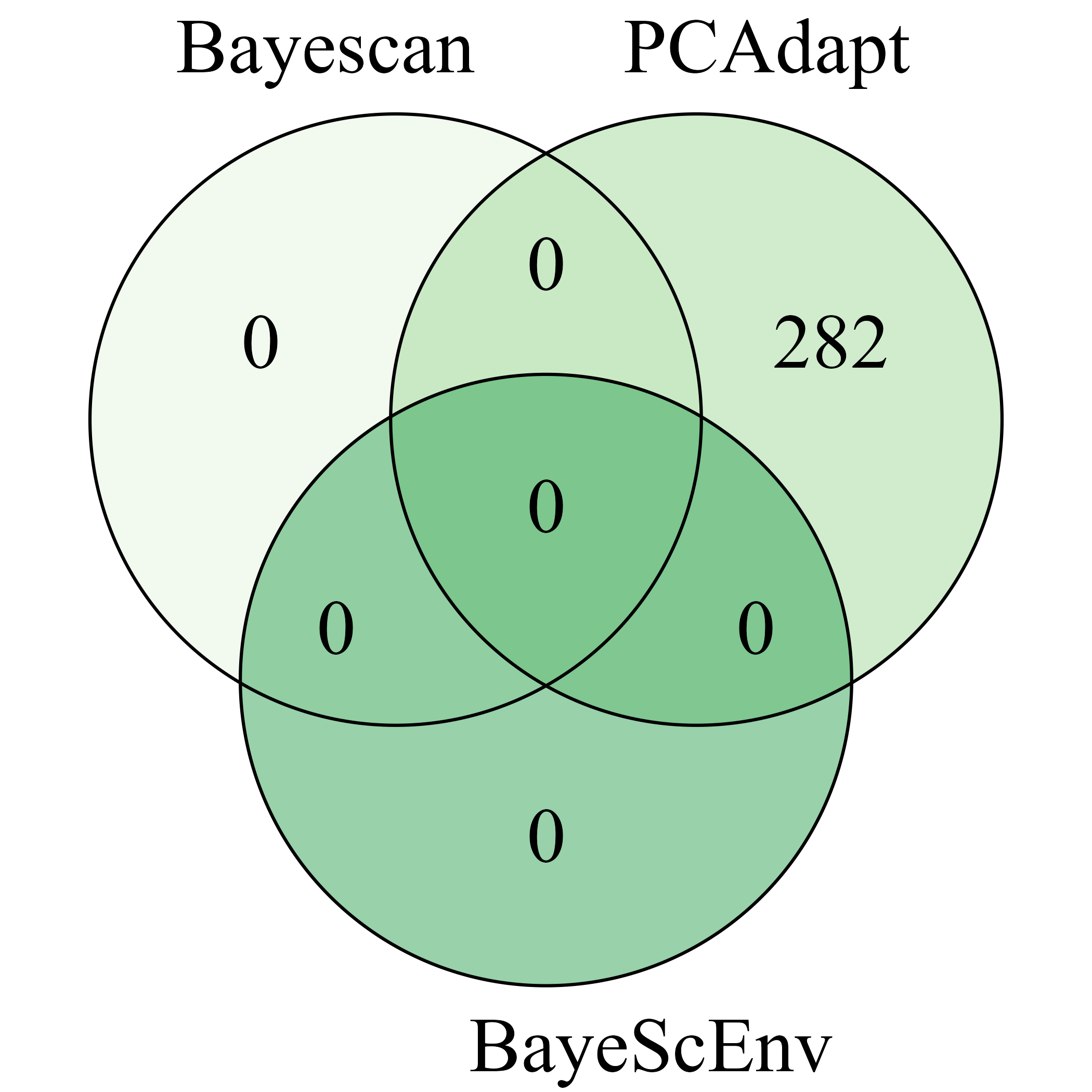

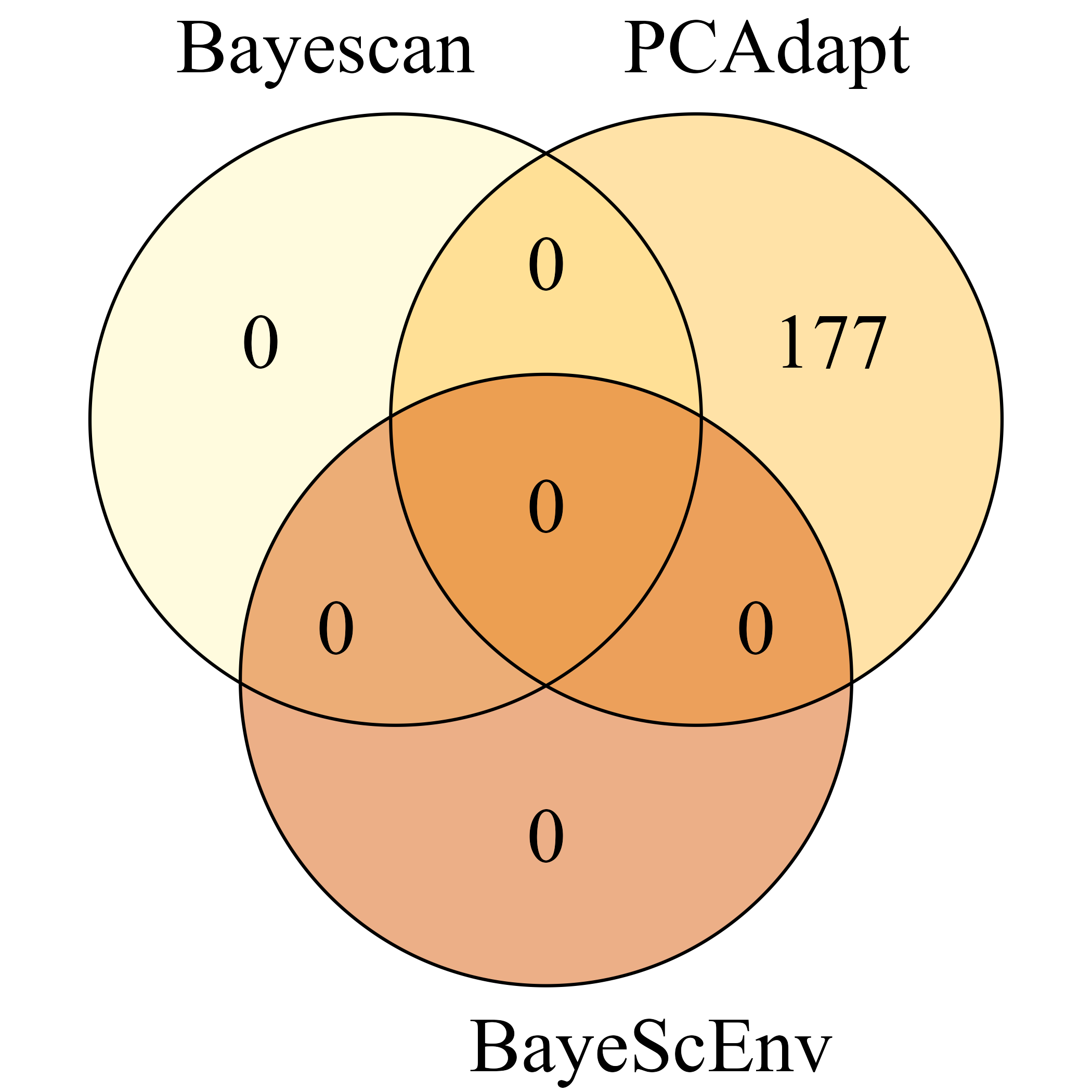

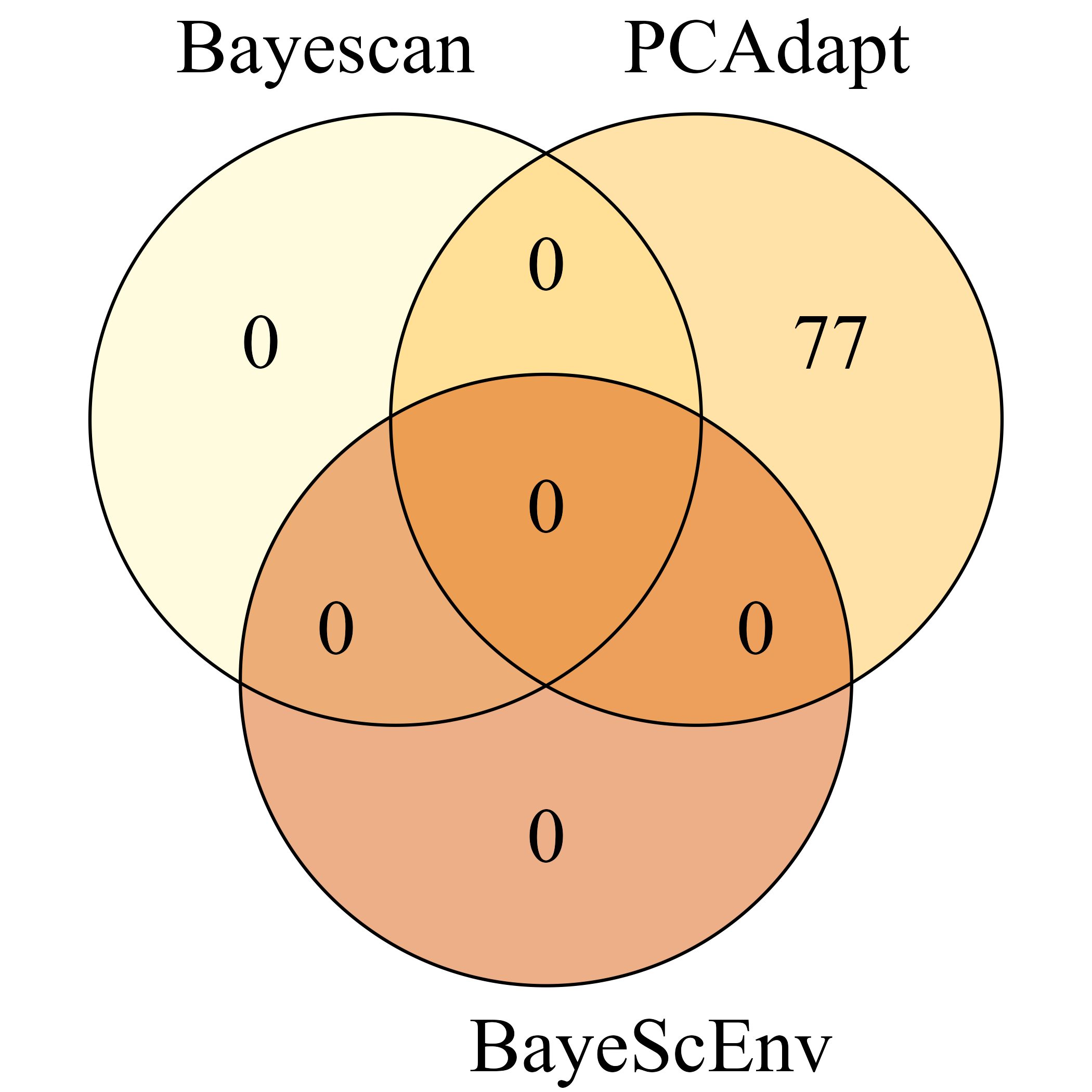

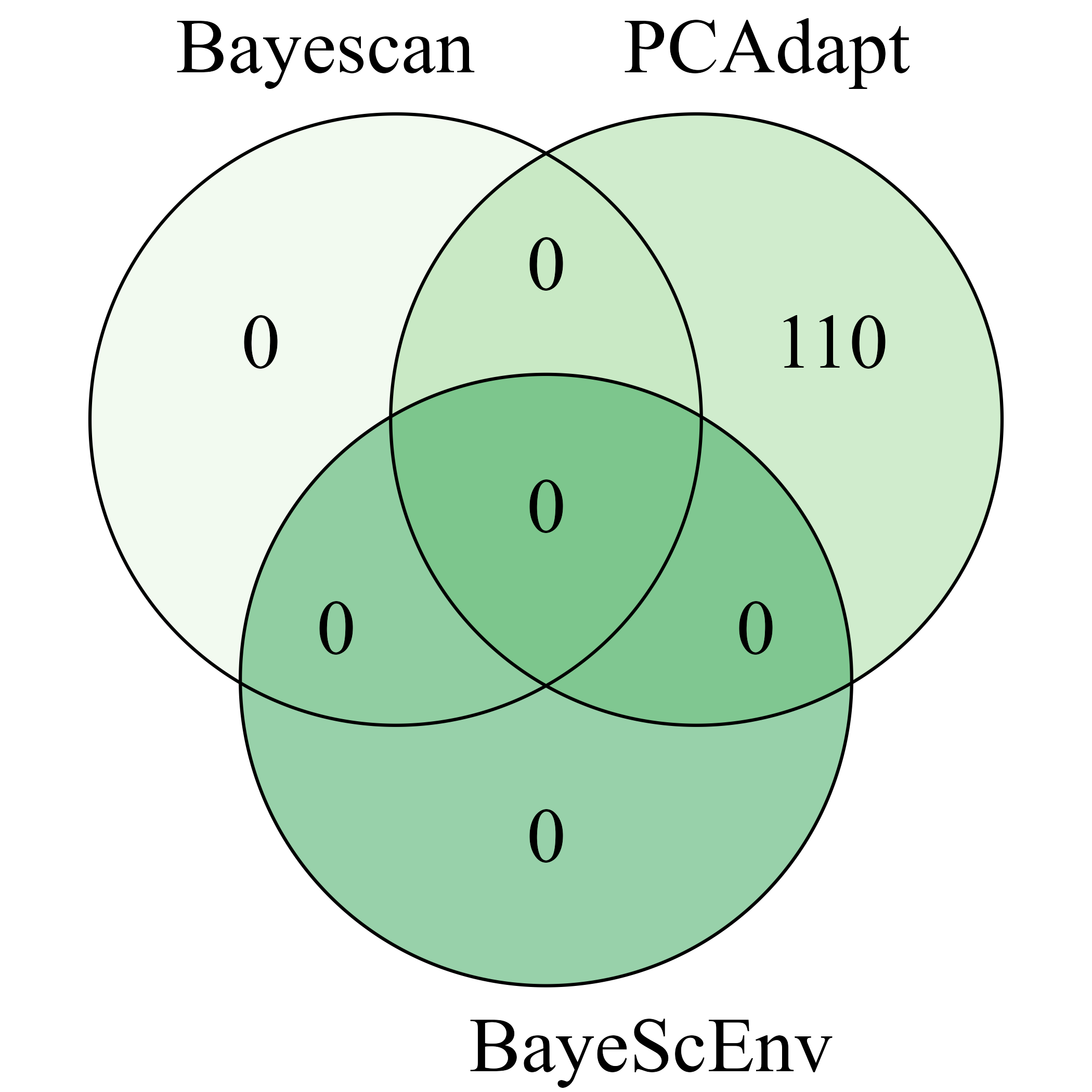

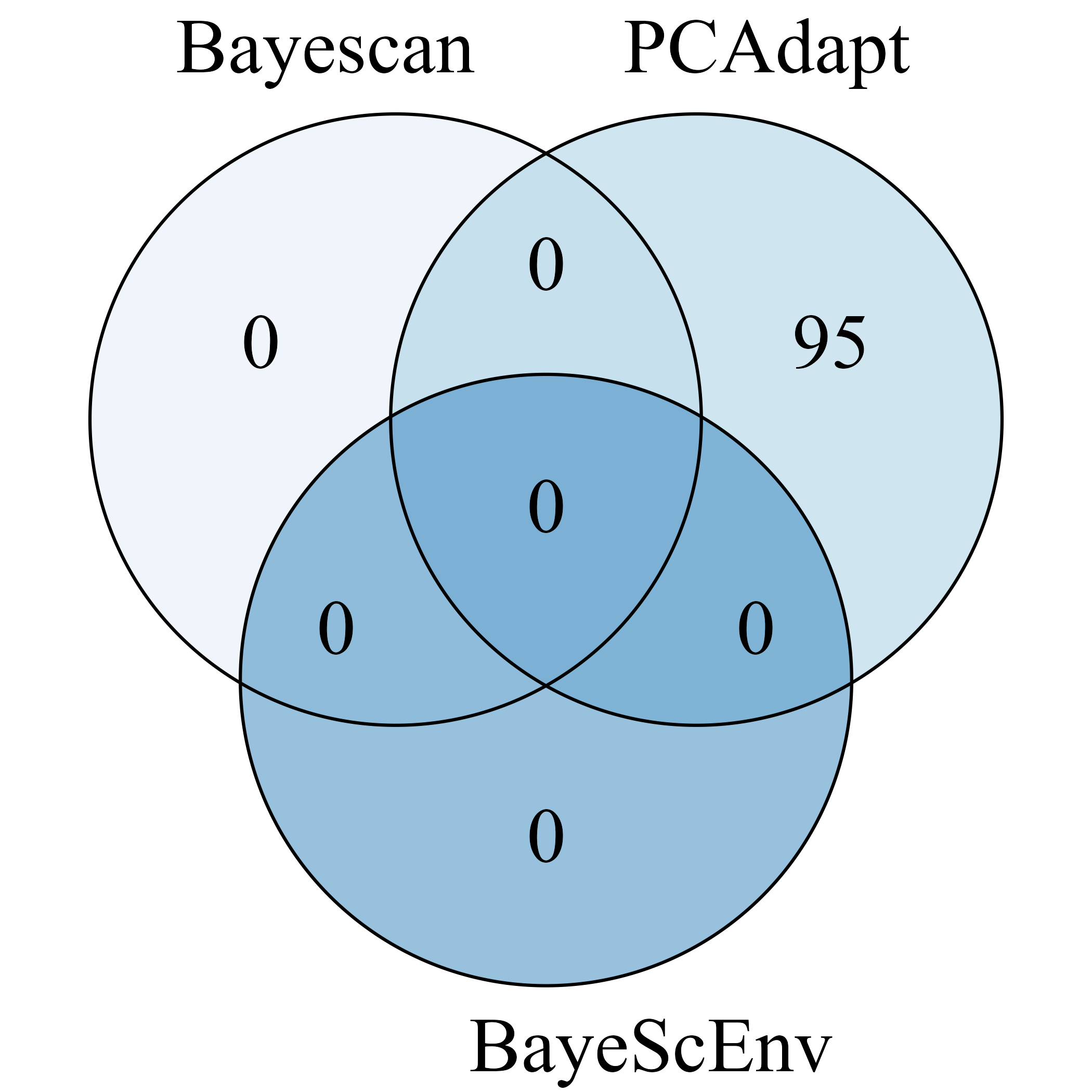

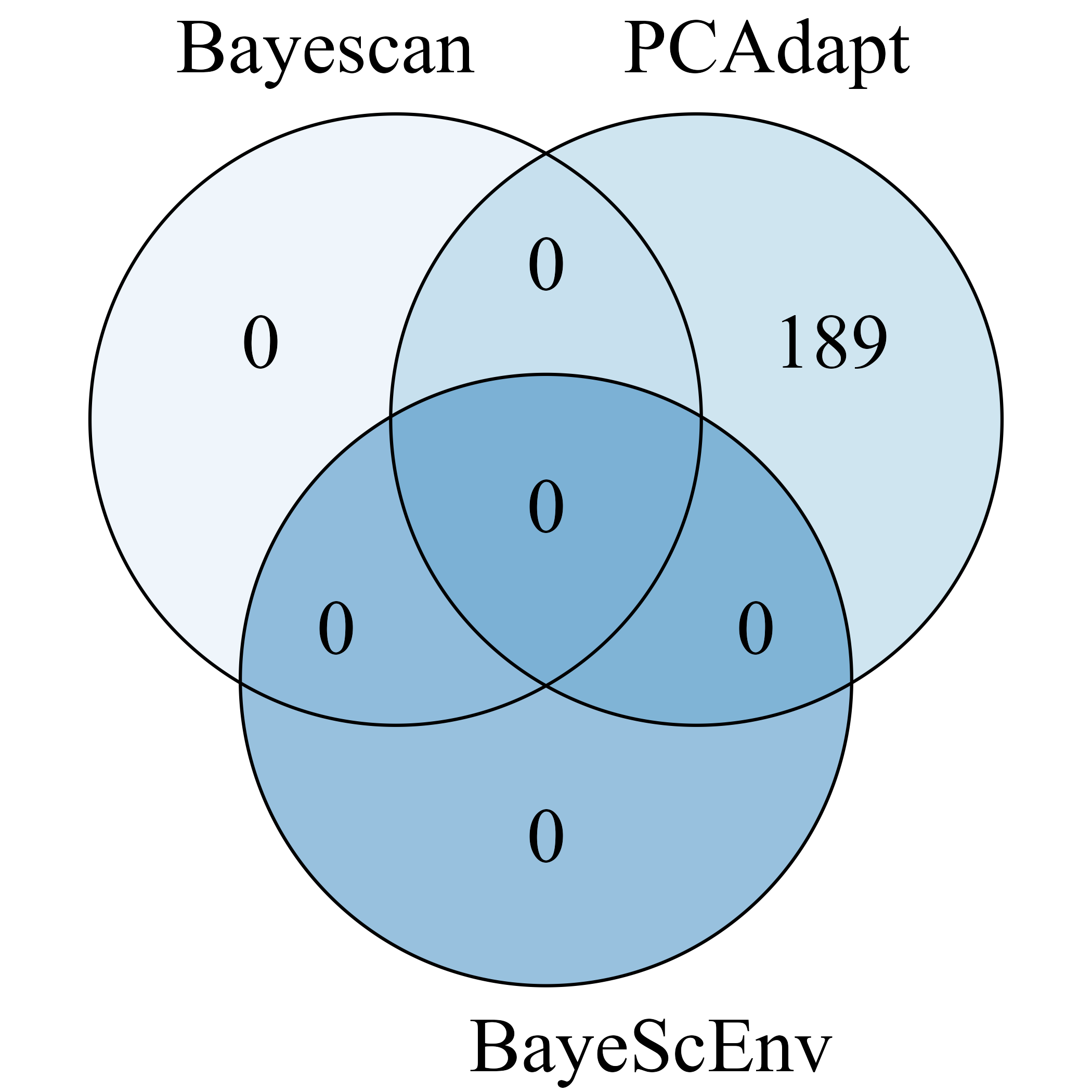

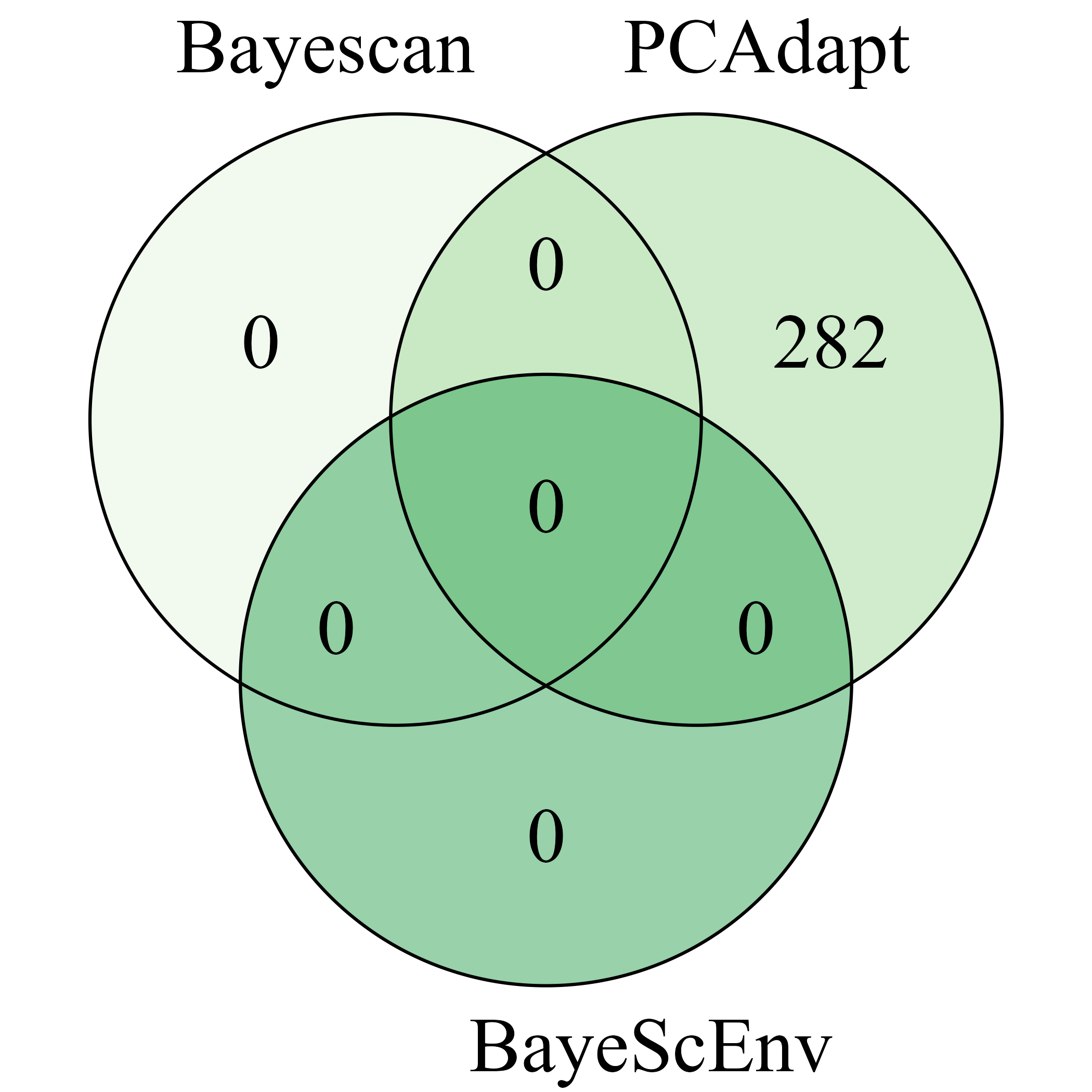

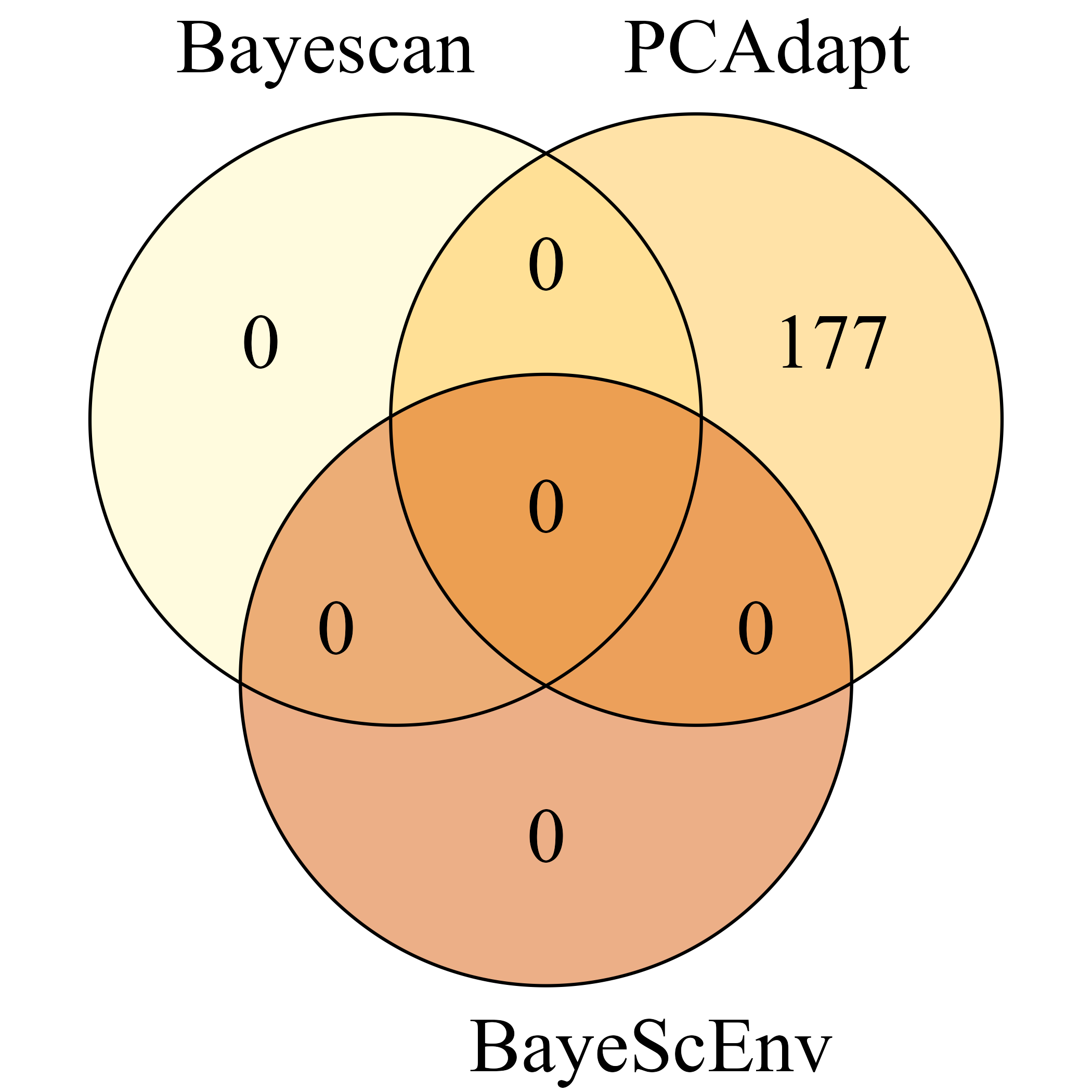

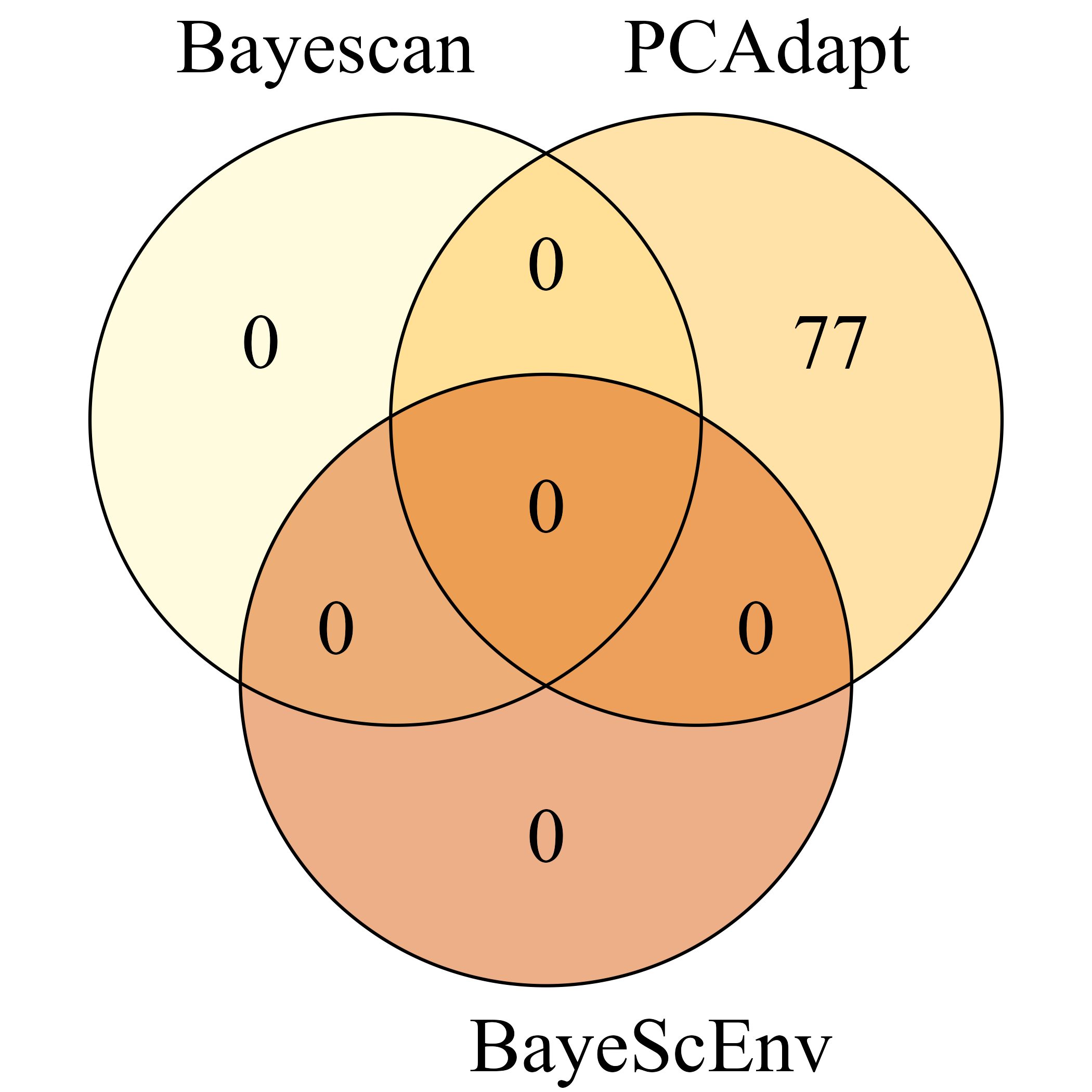

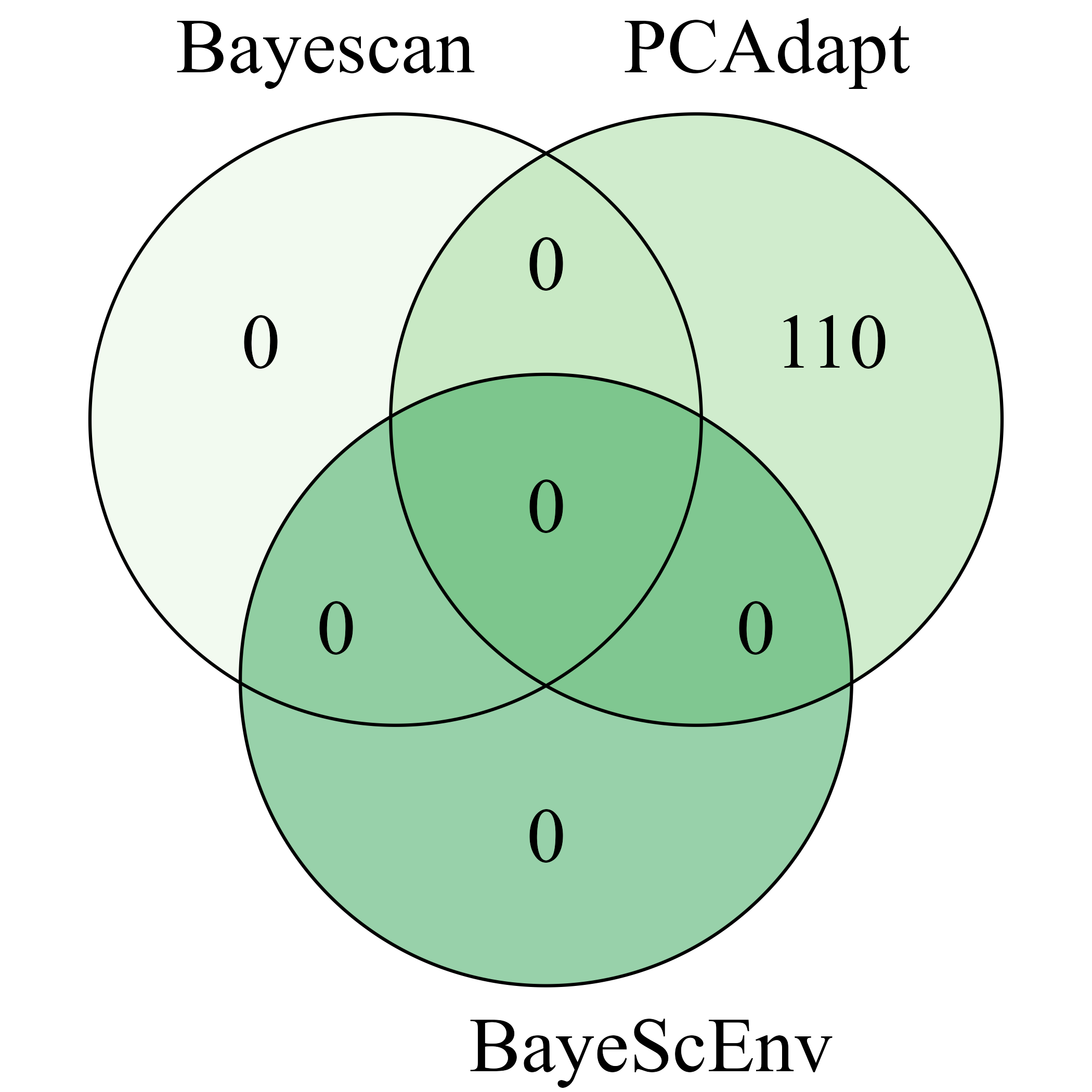

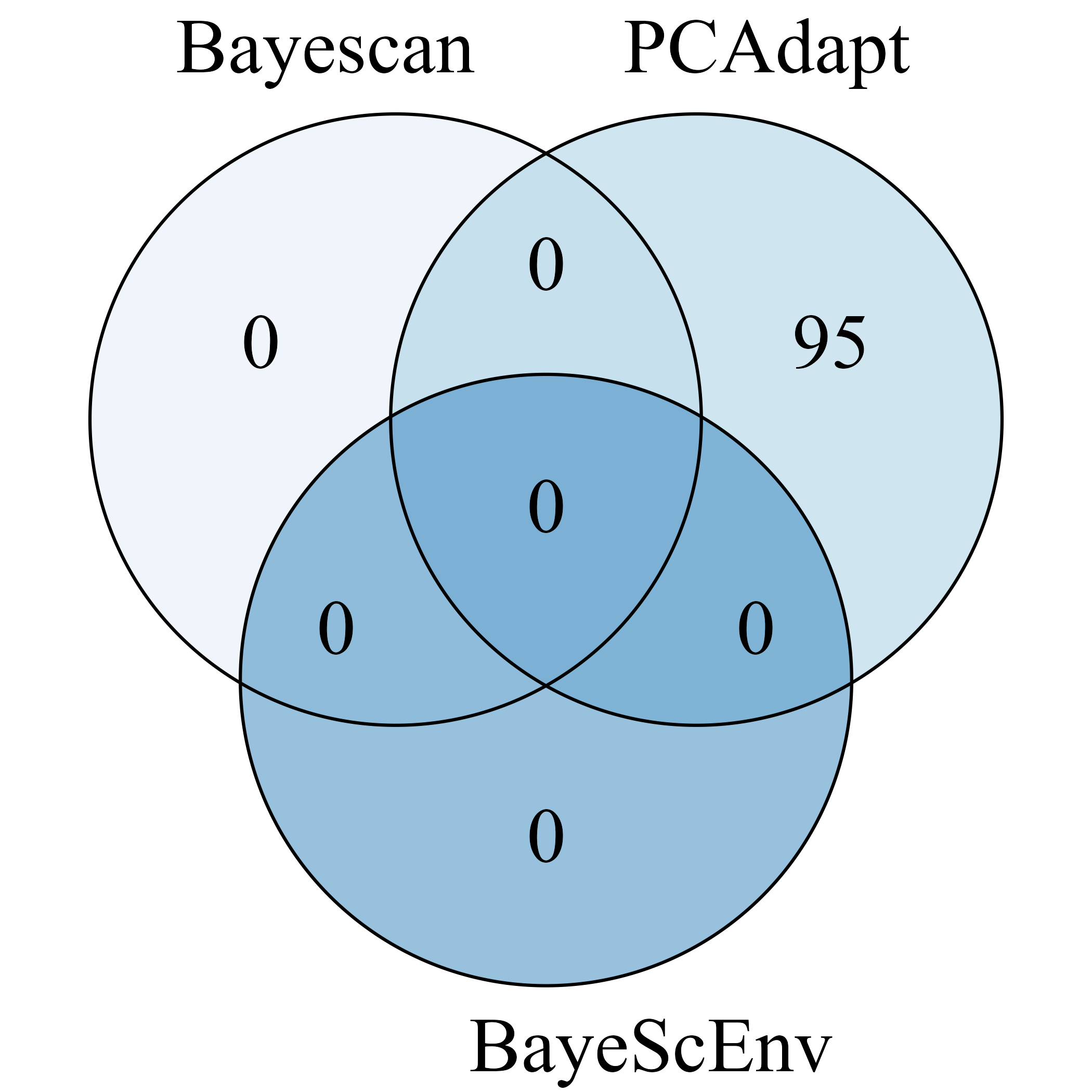


**S5.** Venn Diagrams showing the intersection among three different genome scan approaches (PCAdapt, Bayescan, BayeScEnv) to detect loci under selection using SNPs obtained from bwa+dDocent (in green), bowtie2+Stacks (in blue) and bwa+Stacks (in orange). The first two diagrams above show the overlap for the Koutajoki dataset, while the second two diagrams below show the overlap for the Oulujoki dataset.

**
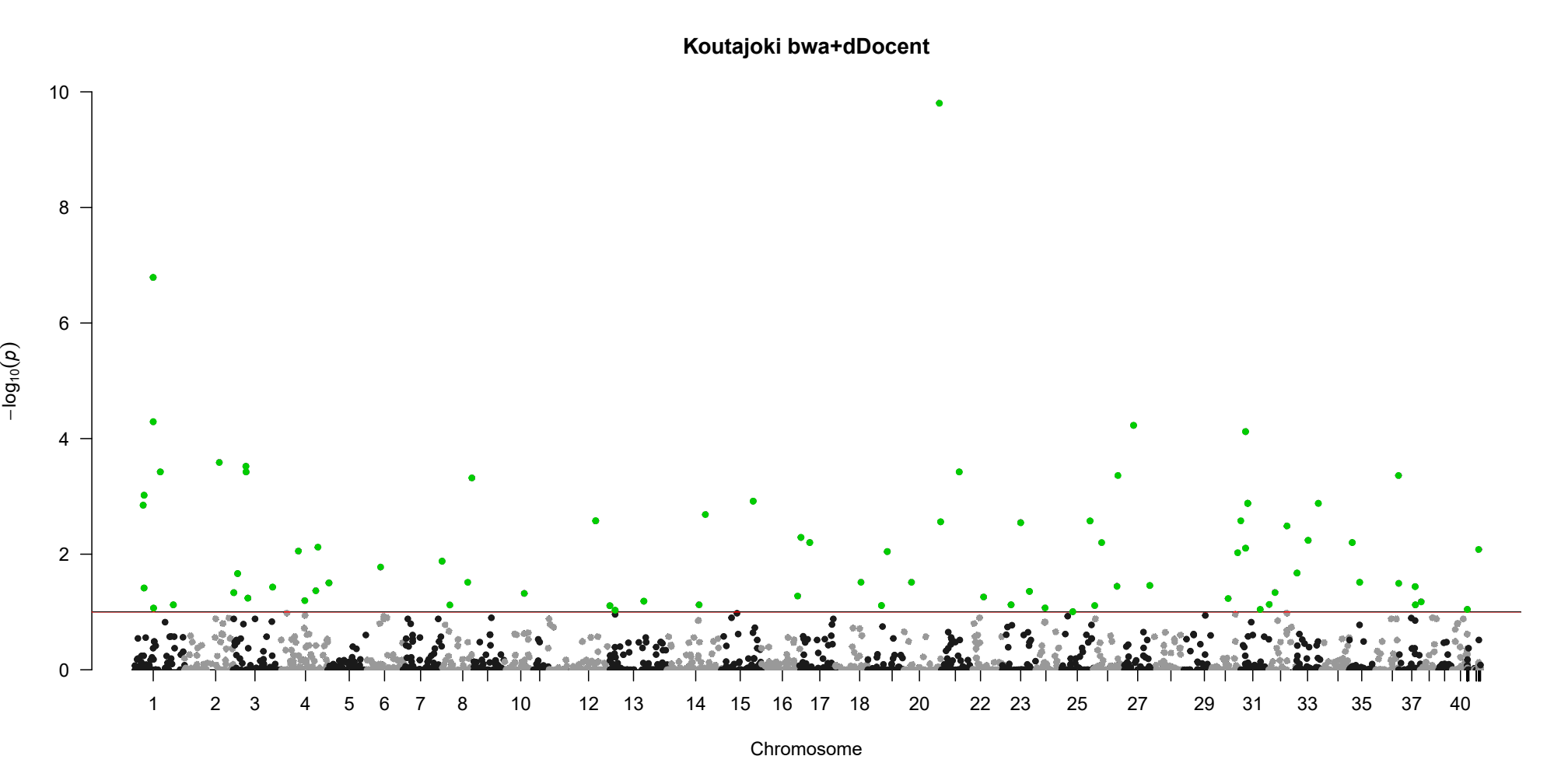
**

A

**
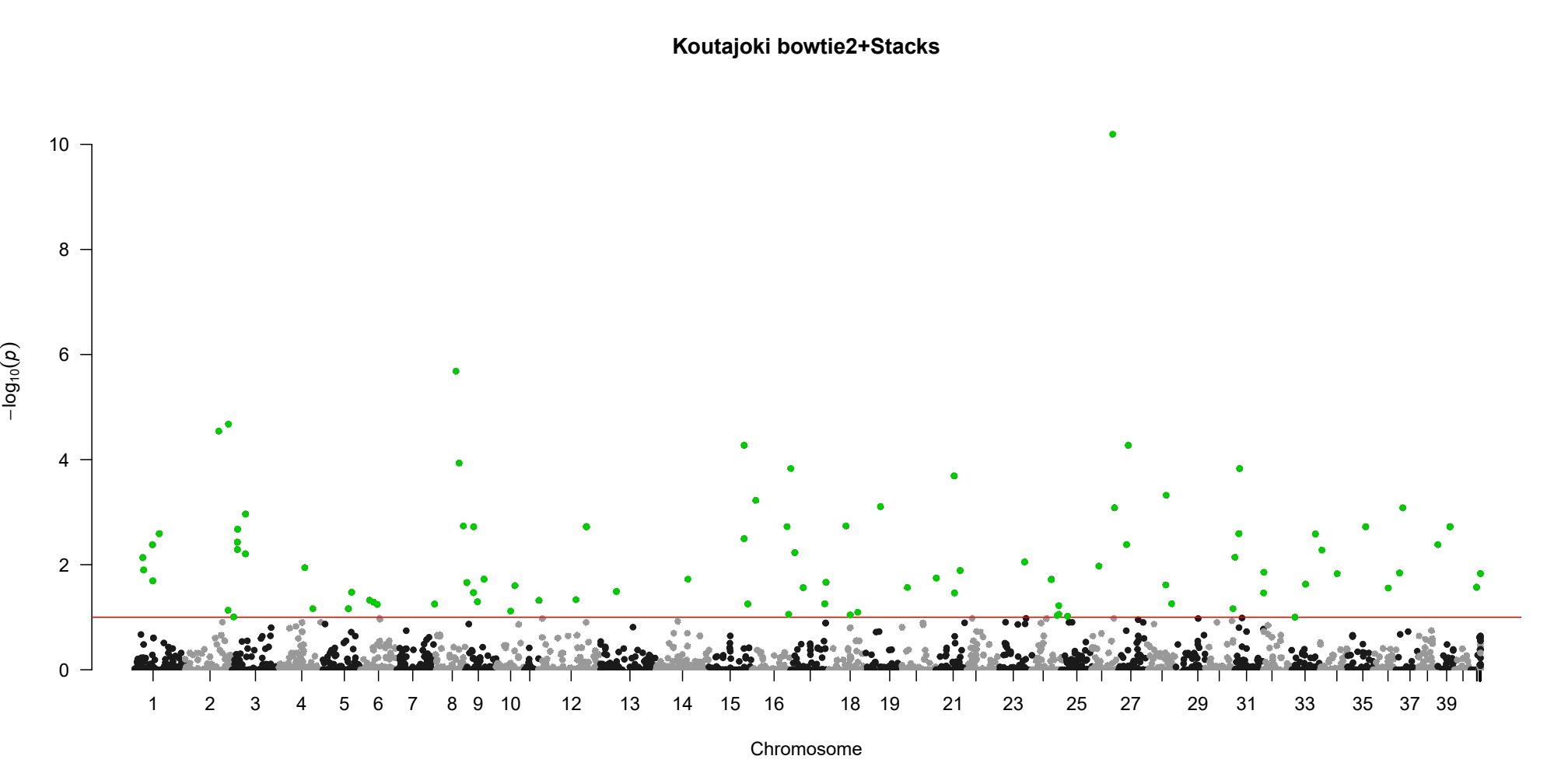
**

B

**
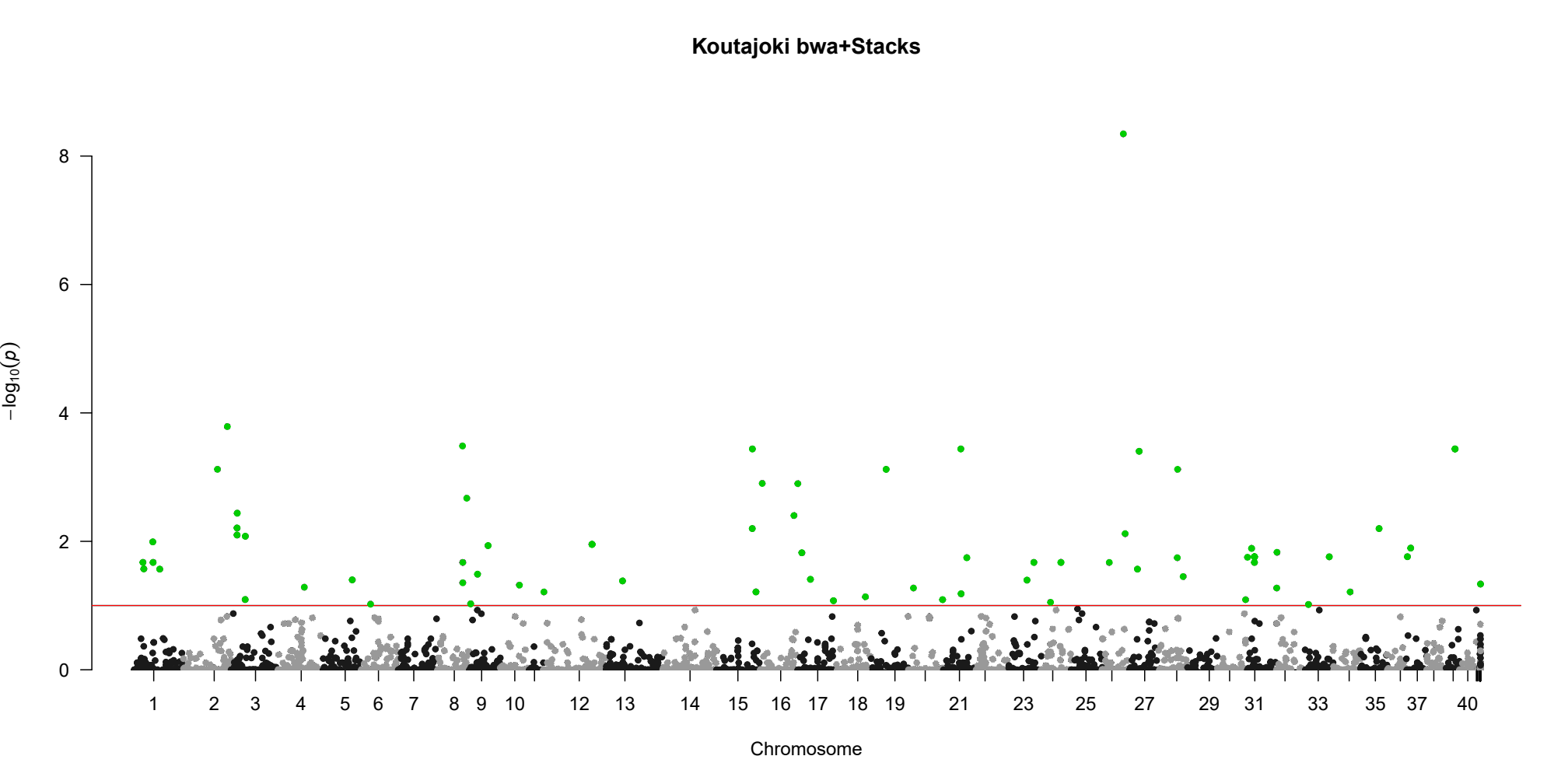
**

C

**
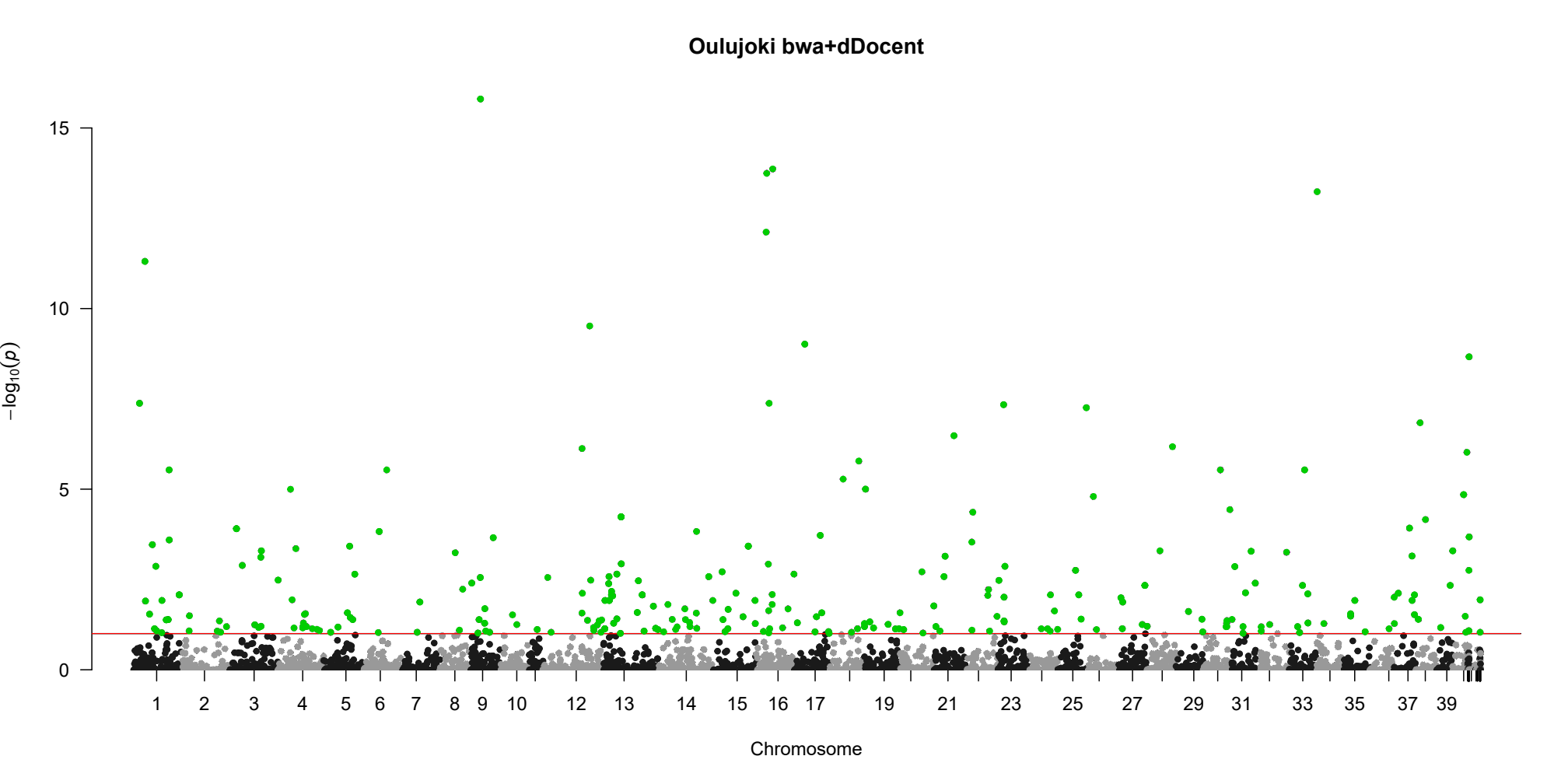
**

D

**
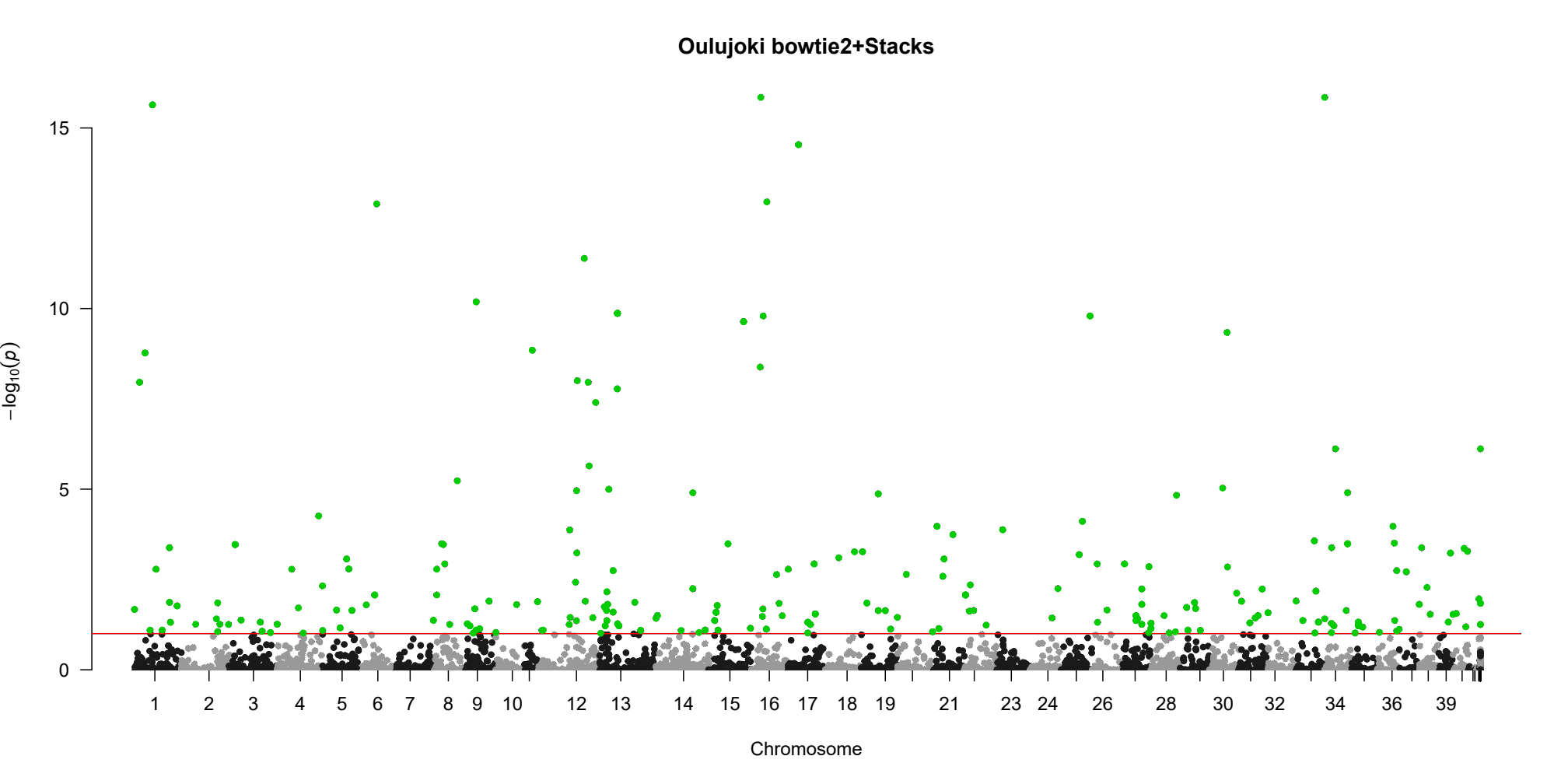
**

E

**
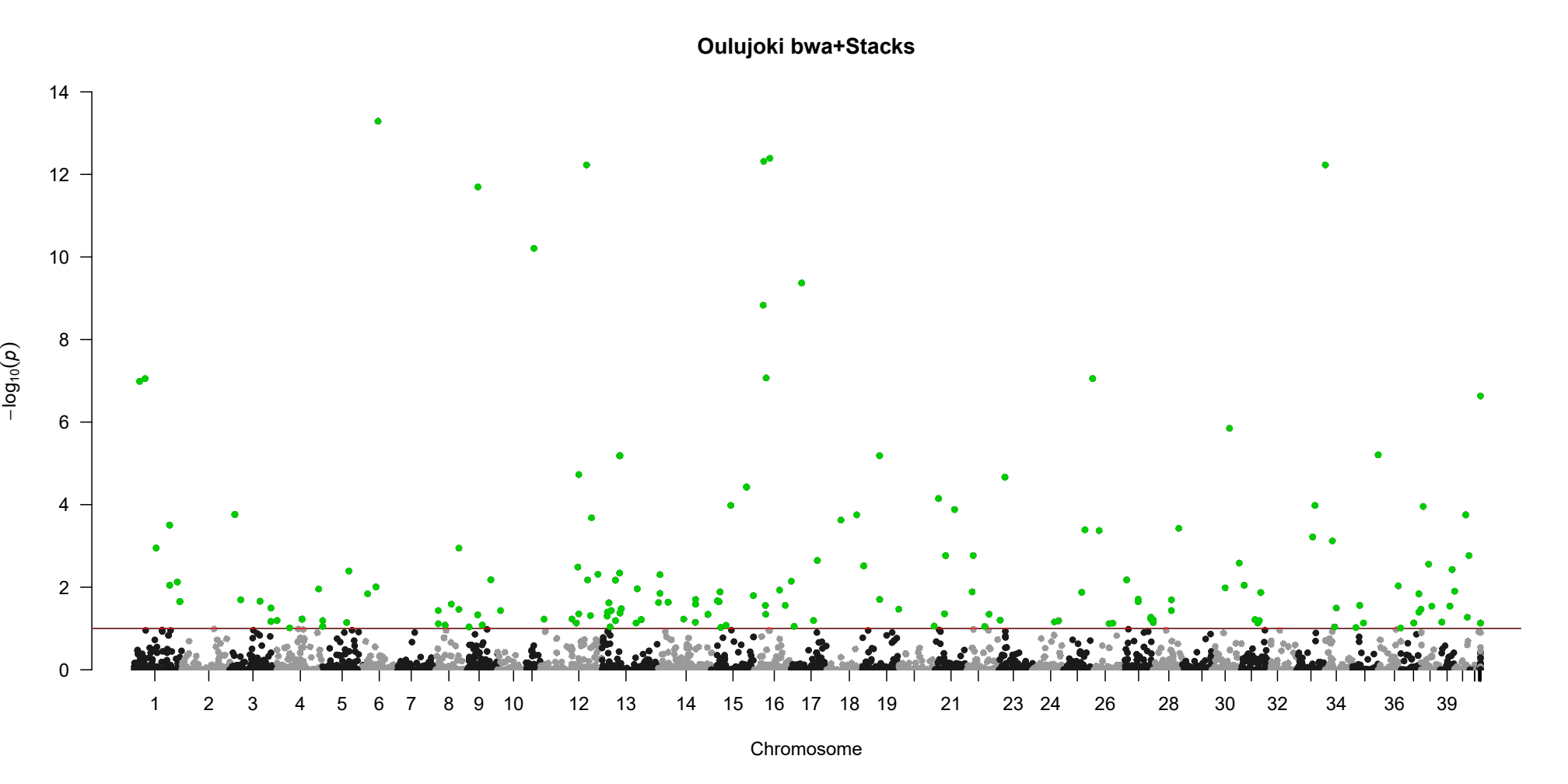
**

F

**S6.** Manhattan plot showing the distribution of the SNPs found in each dataset and watershed by chromosome (x-axis) and the log-transformation of the p-values (-log_10_(p)) on the y-axis. (A) Koutajoki bwa+dDocent; (B) Koutajoki bowtie2+Stacks; (C) Koutajoki bwa+Stacks; (D) Oulujoki bwa+dDocent; (E) Oulujoki bowtie2+Stacks; (F) Oulujoki bwa+Stacks. SNPs in green are the outliers found in each dataset using PCAdapt.

**S7.** List of the all the outliers overlapping between pipelines (bwa+dDocent, bowtie2+Stacks and bwa+Stacks) and watersheds (Koutajoki and Oulujoki), that did not show any known association with migration/residency. For each outlier it is shown the position, the consequence and the closest gene according to Variant Effect Predictor (VEP) in Ensembl. The maximum distance for a SNP to be considered close to a gene was set at 5 kb Upstream/Downstream. As a result, some SNPs in the table are associated with multiple nearby genes, since they fall within 10 kb of more than one gene.

| CHR | Location | REF | Consequence | Symbol | Gene |
| --- | --- | --- | --- | --- | --- |
| [1](https://www.ensembl.org/Salmo_trutta/Location/View?contigviewbottom=variation_feature_variation%3Dnormal;db=core;r=1:11220974-11221074;tl=RpFid1fPWgCVa7tG-11457638) | 11221024 | A | intergenic_variant | - | - |
| [1](https://www.ensembl.org/Salmo_trutta/Location/View?contigviewbottom=variation_feature_variation%3Dnormal;db=core;r=1:19660349-19660449;tl=RpFid1fPWgCVa7tG-11457638) | 19660399 | C | intron_variant | usta | [ENSSTUG00000011441](https://www.ensembl.org/Salmo_trutta/Gene/Summary?db=core;g=ENSSTUG00000011441;tl=RpFid1fPWgCVa7tG-11457638) |
| [1](https://www.ensembl.org/Salmo_trutta/Location/View?contigviewbottom=variation_feature_variation%3Dnormal;db=core;r=1:31161961-31162061;tl=RpFid1fPWgCVa7tG-11457638) | 31162011 | G | downstream_gene_variant | ginm1 | [ENSSTUG00000035148](https://www.ensembl.org/Salmo_trutta/Gene/Summary?db=core;g=ENSSTUG00000035148;tl=RpFid1fPWgCVa7tG-11457638) |
| [1](https://www.ensembl.org/Salmo_trutta/Location/View?contigviewbottom=variation_feature_variation%3Dnormal;db=core;r=1:31161961-31162061;tl=RpFid1fPWgCVa7tG-11457638) | 31162011 | G | intron_variant | katna1 | [ENSSTUG00000035084](https://www.ensembl.org/Salmo_trutta/Gene/Summary?db=core;g=ENSSTUG00000035084;tl=RpFid1fPWgCVa7tG-11457638) |
| [1](https://www.ensembl.org/Salmo_trutta/Location/View?contigviewbottom=variation_feature_variation%3Dnormal;db=core;r=1:31161961-31162061;tl=RpFid1fPWgCVa7tG-11457638) | 31162011 | G | downstream_gene_variant | lats1 | [ENSSTUG00000035063](https://www.ensembl.org/Salmo_trutta/Gene/Summary?db=core;g=ENSSTUG00000035063;tl=RpFid1fPWgCVa7tG-11457638) |
| [1](https://www.ensembl.org/Salmo_trutta/Location/View?contigviewbottom=variation_feature_variation%3Dnormal;db=core;r=1:31161961-31162061;tl=RpFid1fPWgCVa7tG-11457638) | 31162011 | G | upstream_gene_variant | ppil4 | [ENSSTUG00000035181](https://www.ensembl.org/Salmo_trutta/Gene/Summary?db=core;g=ENSSTUG00000035181;tl=RpFid1fPWgCVa7tG-11457638) |
| [1](https://www.ensembl.org/Salmo_trutta/Location/View?contigviewbottom=variation_feature_variation%3Dnormal;db=core;r=1:36608019-36608119;tl=RpFid1fPWgCVa7tG-11457638) | 36608069 | G | downstream_gene_variant | asb2a.1 | [ENSSTUG00000034582](https://www.ensembl.org/Salmo_trutta/Gene/Summary?db=core;g=ENSSTUG00000034582;tl=RpFid1fPWgCVa7tG-11457638) |
| [1](https://www.ensembl.org/Salmo_trutta/Location/View?contigviewbottom=variation_feature_variation%3Dnormal;db=core;r=1:45760981-45761081;tl=RpFid1fPWgCVa7tG-11457638) | 45761031 | T | downstream_gene_variant | - | [ENSSTUG00000001056](https://www.ensembl.org/Salmo_trutta/Gene/Summary?db=core;g=ENSSTUG00000001056;tl=RpFid1fPWgCVa7tG-11457638) |
| [1](https://www.ensembl.org/Salmo_trutta/Location/View?contigviewbottom=variation_feature_variation%3Dnormal;db=core;r=1:46075192-46075292;tl=RpFid1fPWgCVa7tG-11457638) | 46075242 | A | intron_variant | - | [ENSSTUG00000001794](https://www.ensembl.org/Salmo_trutta/Gene/Summary?db=core;g=ENSSTUG00000001794;tl=RpFid1fPWgCVa7tG-11457638) |
| [1](https://www.ensembl.org/Salmo_trutta/Location/View?contigviewbottom=variation_feature_variation%3Dnormal;db=core;r=1:57258953-57259053;tl=RpFid1fPWgCVa7tG-11457638) | 57259003 | C | intron_variant | NHERF1 | [ENSSTUG00000018321](https://www.ensembl.org/Salmo_trutta/Gene/Summary?db=core;g=ENSSTUG00000018321;tl=RpFid1fPWgCVa7tG-11457638) |
| [1](https://www.ensembl.org/Salmo_trutta/Location/View?contigviewbottom=variation_feature_variation%3Dnormal;db=core;r=1:57386489-57386589;tl=RpFid1fPWgCVa7tG-11457638) | 57386539 | C | upstream_gene_variant | CASKIN2 | [ENSSTUG00000018327](https://www.ensembl.org/Salmo_trutta/Gene/Summary?db=core;g=ENSSTUG00000018327;tl=RpFid1fPWgCVa7tG-11457638) |
| [1](https://www.ensembl.org/Salmo_trutta/Location/View?contigviewbottom=variation_feature_variation%3Dnormal;db=core;r=1:57386489-57386589;tl=RpFid1fPWgCVa7tG-11457638) | 57386539 | C | upstream_gene_variant | ST6GALNAC2 | [ENSSTUG00000018476](https://www.ensembl.org/Salmo_trutta/Gene/Summary?db=core;g=ENSSTUG00000018476;tl=RpFid1fPWgCVa7tG-11457638) |
| [1](https://www.ensembl.org/Salmo_trutta/Location/View?contigviewbottom=variation_feature_variation%3Dnormal;db=core;r=1:57386489-57386589;tl=RpFid1fPWgCVa7tG-11457638) | 57386539 | C | downstream_gene_variant | - | [ENSSTUG00000018491](https://www.ensembl.org/Salmo_trutta/Gene/Summary?db=core;g=ENSSTUG00000018491;tl=RpFid1fPWgCVa7tG-11457638) |
| [1](https://www.ensembl.org/Salmo_trutta/Location/View?contigviewbottom=variation_feature_variation%3Dnormal;db=core;r=1:58783438-58783538;tl=RpFid1fPWgCVa7tG-11457638) | 58783488 | G | downstream_gene_variant | dhrs7ca | [ENSSTUG00000017593](https://www.ensembl.org/Salmo_trutta/Gene/Summary?db=core;g=ENSSTUG00000017593;tl=RpFid1fPWgCVa7tG-11457638) |
| [1](https://www.ensembl.org/Salmo_trutta/Location/View?contigviewbottom=variation_feature_variation%3Dnormal;db=core;r=1:58783438-58783538;tl=RpFid1fPWgCVa7tG-11457638) | 58783488 | G | downstream_gene_variant | im:6904045 | [ENSSTUG00000017587](https://www.ensembl.org/Salmo_trutta/Gene/Summary?db=core;g=ENSSTUG00000017587;tl=RpFid1fPWgCVa7tG-11457638) |
| [1](https://www.ensembl.org/Salmo_trutta/Location/View?contigviewbottom=variation_feature_variation%3Dnormal;db=core;r=1:58783438-58783538;tl=RpFid1fPWgCVa7tG-11457638) | 58783488 | G | upstream_gene_variant | - | [ENSSTUG00000017585](https://www.ensembl.org/Salmo_trutta/Gene/Summary?db=core;g=ENSSTUG00000017585;tl=RpFid1fPWgCVa7tG-11457638) |
| [1](https://www.ensembl.org/Salmo_trutta/Location/View?contigviewbottom=variation_feature_variation%3Dnormal;db=core;r=1:69128259-69128359;tl=RpFid1fPWgCVa7tG-11457638) | 69128309 | G | downstream_gene_variant | parp14rs1 | [ENSSTUG00000000946](https://www.ensembl.org/Salmo_trutta/Gene/Summary?db=core;g=ENSSTUG00000000946;tl=RpFid1fPWgCVa7tG-11457638) |
| [2](https://www.ensembl.org/Salmo_trutta/Location/View?contigviewbottom=variation_feature_variation%3Dnormal;db=core;r=2:55064892-55064992;tl=RpFid1fPWgCVa7tG-11457638) | 55064942 | A | intron_variant | get4 | [ENSSTUG00000031741](https://www.ensembl.org/Salmo_trutta/Gene/Summary?db=core;g=ENSSTUG00000031741;tl=RpFid1fPWgCVa7tG-11457638) |
| [2](https://www.ensembl.org/Salmo_trutta/Location/View?contigviewbottom=variation_feature_variation%3Dnormal;db=core;r=2:60304085-60304185;tl=RpFid1fPWgCVa7tG-11457638) | 60304135 | G | upstream_gene_variant | slc6a16b | [ENSSTUG00000012847](https://www.ensembl.org/Salmo_trutta/Gene/Summary?db=core;g=ENSSTUG00000012847;tl=RpFid1fPWgCVa7tG-11457638) |
| [3](https://www.ensembl.org/Salmo_trutta/Location/View?contigviewbottom=variation_feature_variation%3Dnormal;db=core;r=3:9902627-9902727;tl=RpFid1fPWgCVa7tG-11457638) | 9902677 | T | intron_variant | - | [ENSSTUG00000014278](https://www.ensembl.org/Salmo_trutta/Gene/Summary?db=core;g=ENSSTUG00000014278;tl=RpFid1fPWgCVa7tG-11457638) |
| [3](https://www.ensembl.org/Salmo_trutta/Location/View?contigviewbottom=variation_feature_variation%3Dnormal;db=core;r=3:9902667-9902767;tl=RpFid1fPWgCVa7tG-11457638) | 9902717 | C | intron_variant | - | [ENSSTUG00000014278](https://www.ensembl.org/Salmo_trutta/Gene/Summary?db=core;g=ENSSTUG00000014278;tl=RpFid1fPWgCVa7tG-11457638) |
| [3](https://www.ensembl.org/Salmo_trutta/Location/View?contigviewbottom=variation_feature_variation%3Dnormal;db=core;r=3:18972464-18972564;tl=RpFid1fPWgCVa7tG-11457638) | 18972514 | T | intron_variant | med12 | [ENSSTUG00000011833](https://www.ensembl.org/Salmo_trutta/Gene/Summary?db=core;g=ENSSTUG00000011833;tl=RpFid1fPWgCVa7tG-11457638) |
| [3](https://www.ensembl.org/Salmo_trutta/Location/View?contigviewbottom=variation_feature_variation%3Dnormal;db=core;r=3:38413640-38413740;tl=RpFid1fPWgCVa7tG-11457638) | 38413690 | G | intron_variant | si:zfos-2326c3.2 | [ENSSTUG00000042915](https://www.ensembl.org/Salmo_trutta/Gene/Summary?db=core;g=ENSSTUG00000042915;tl=RpFid1fPWgCVa7tG-11457638) |
| [3](https://www.ensembl.org/Salmo_trutta/Location/View?contigviewbottom=variation_feature_variation%3Dnormal;db=core;r=3:48594893-48594993;tl=RpFid1fPWgCVa7tG-11457638) | 48594943 | T | intron_variant | - | [ENSSTUG00000028932](https://www.ensembl.org/Salmo_trutta/Gene/Summary?db=core;g=ENSSTUG00000028932;tl=RpFid1fPWgCVa7tG-11457638) |
| [3](https://www.ensembl.org/Salmo_trutta/Location/View?contigviewbottom=variation_feature_variation%3Dnormal;db=core;r=3:74590719-74590819;tl=RpFid1fPWgCVa7tG-11457638) | 74590769 | C | intron_variant | ca14 | [ENSSTUG00000000877](https://www.ensembl.org/Salmo_trutta/Gene/Summary?db=core;g=ENSSTUG00000000877;tl=RpFid1fPWgCVa7tG-11457638) |
| [3](https://www.ensembl.org/Salmo_trutta/Location/View?contigviewbottom=variation_feature_variation%3Dnormal;db=core;r=3:74590719-74590819;tl=RpFid1fPWgCVa7tG-11457638) | 74590769 | C | intron_variant | - | [ENSSTUG00000000868](https://www.ensembl.org/Salmo_trutta/Gene/Summary?db=core;g=ENSSTUG00000000868;tl=RpFid1fPWgCVa7tG-11457638) |
| [4](https://www.ensembl.org/Salmo_trutta/Location/View?contigviewbottom=variation_feature_variation%3Dnormal;db=core;r=4:35409080-35409180;tl=RpFid1fPWgCVa7tG-11457638) | 35409130 | A | intergenic_variant | - | - |
| [4](https://www.ensembl.org/Salmo_trutta/Location/View?contigviewbottom=variation_feature_variation%3Dnormal;db=core;r=4:42888888-42888988;tl=RpFid1fPWgCVa7tG-11457638) | 42888938 | A | intron_variant | SCFD2 | [ENSSTUG00000005961](https://www.ensembl.org/Salmo_trutta/Gene/Summary?db=core;g=ENSSTUG00000005961;tl=RpFid1fPWgCVa7tG-11457638) |
| [4](https://www.ensembl.org/Salmo_trutta/Location/View?contigviewbottom=variation_feature_variation%3Dnormal;db=core;r=4:64627080-64627180;tl=RpFid1fPWgCVa7tG-11457638) | 64627130 | A | upstream_gene_variant | - | [ENSSTUG00000014399](https://www.ensembl.org/Salmo_trutta/Gene/Summary?db=core;g=ENSSTUG00000014399;tl=RpFid1fPWgCVa7tG-11457638) |
| [4](https://www.ensembl.org/Salmo_trutta/Location/View?contigviewbottom=variation_feature_variation%3Dnormal;db=core;r=4:66600260-66600360;tl=RpFid1fPWgCVa7tG-11457638) | 66600310 | A | intergenic_variant | - | - |
| [4](https://www.ensembl.org/Salmo_trutta/Location/View?contigviewbottom=variation_feature_variation%3Dnormal;db=core;r=4:72489551-72489651;tl=RpFid1fPWgCVa7tG-11457638) | 72489601 | A | intron_variant | dapk2b | [ENSSTUG00000016347](https://www.ensembl.org/Salmo_trutta/Gene/Summary?db=core;g=ENSSTUG00000016347;tl=RpFid1fPWgCVa7tG-11457638) |
| [4](https://www.ensembl.org/Salmo_trutta/Location/View?contigviewbottom=variation_feature_variation%3Dnormal;db=core;r=4:72824006-72824106;tl=RpFid1fPWgCVa7tG-11457638) | 72824056 | G | upstream_gene_variant | bnip2 | [ENSSTUG00000016629](https://www.ensembl.org/Salmo_trutta/Gene/Summary?db=core;g=ENSSTUG00000016629;tl=RpFid1fPWgCVa7tG-11457638) |
| [5](https://www.ensembl.org/Salmo_trutta/Location/View?contigviewbottom=variation_feature_variation%3Dnormal;db=core;r=5:41212427-41212527;tl=RpFid1fPWgCVa7tG-11457638) | 41212477 | T | intron_variant | nrxn2b | [ENSSTUG00000046980](https://www.ensembl.org/Salmo_trutta/Gene/Summary?db=core;g=ENSSTUG00000046980;tl=RpFid1fPWgCVa7tG-11457638) |
| [5](https://www.ensembl.org/Salmo_trutta/Location/View?contigviewbottom=variation_feature_variation%3Dnormal;db=core;r=5:44658866-44658966;tl=RpFid1fPWgCVa7tG-11457638) | 44658916 | A | 5_prime_UTR_variant | SEC24C | [ENSSTUG00000002697](https://www.ensembl.org/Salmo_trutta/Gene/Summary?db=core;g=ENSSTUG00000002697;tl=RpFid1fPWgCVa7tG-11457638) |
| [5](https://www.ensembl.org/Salmo_trutta/Location/View?contigviewbottom=variation_feature_variation%3Dnormal;db=core;r=5:44658866-44658966;tl=RpFid1fPWgCVa7tG-11457638) | 44658916 | A | missense_variant | - | [ENSSTUG00000005339](https://www.ensembl.org/Salmo_trutta/Gene/Summary?db=core;g=ENSSTUG00000005339;tl=RpFid1fPWgCVa7tG-11457638) |
| [6](https://www.ensembl.org/Salmo_trutta/Location/View?contigviewbottom=variation_feature_variation%3Dnormal;db=core;r=6:10479697-10479797;tl=RpFid1fPWgCVa7tG-11457638) | 10479747 | A | intron_variant | ctnnd2a | [ENSSTUG00000022222](https://www.ensembl.org/Salmo_trutta/Gene/Summary?db=core;g=ENSSTUG00000022222;tl=RpFid1fPWgCVa7tG-11457638) |
| [6](https://www.ensembl.org/Salmo_trutta/Location/View?contigviewbottom=variation_feature_variation%3Dnormal;db=core;r=6:23445642-23445742;tl=RpFid1fPWgCVa7tG-11457638) | 23445692 | T | upstream_gene_variant | anxa13 | [ENSSTUG00000015743](https://www.ensembl.org/Salmo_trutta/Gene/Summary?db=core;g=ENSSTUG00000015743;tl=RpFid1fPWgCVa7tG-11457638) |
| [6](https://www.ensembl.org/Salmo_trutta/Location/View?contigviewbottom=variation_feature_variation%3Dnormal;db=core;r=6:23445649-23445749;tl=RpFid1fPWgCVa7tG-11457638) | 23445699 | G | upstream_gene_variant | anxa13 | [ENSSTUG00000015743](https://www.ensembl.org/Salmo_trutta/Gene/Summary?db=core;g=ENSSTUG00000015743;tl=RpFid1fPWgCVa7tG-11457638) |
| [6](https://www.ensembl.org/Salmo_trutta/Location/View?contigviewbottom=variation_feature_variation%3Dnormal;db=core;r=6:26511179-26511279;tl=RpFid1fPWgCVa7tG-11457638) | 26511229 | A | upstream_gene_variant | ABCF2 | [ENSSTUG00000031241](https://www.ensembl.org/Salmo_trutta/Gene/Summary?db=core;g=ENSSTUG00000031241;tl=RpFid1fPWgCVa7tG-11457638) |
| [6](https://www.ensembl.org/Salmo_trutta/Location/View?contigviewbottom=variation_feature_variation%3Dnormal;db=core;r=6:26511179-26511279;tl=RpFid1fPWgCVa7tG-11457638) | 26511229 | A | 3_prime_UTR_variant | chpf2 | [ENSSTUG00000031785](https://www.ensembl.org/Salmo_trutta/Gene/Summary?db=core;g=ENSSTUG00000031785;tl=RpFid1fPWgCVa7tG-11457638) |
| [6](https://www.ensembl.org/Salmo_trutta/Location/View?contigviewbottom=variation_feature_variation%3Dnormal;db=core;r=6:26511179-26511279;tl=RpFid1fPWgCVa7tG-11457638) | 26511229 | A | downstream_gene_variant | smarcd3b | [ENSSTUG00000031852](https://www.ensembl.org/Salmo_trutta/Gene/Summary?db=core;g=ENSSTUG00000031852;tl=RpFid1fPWgCVa7tG-11457638) |
| [8](https://www.ensembl.org/Salmo_trutta/Location/View?contigviewbottom=variation_feature_variation%3Dnormal;db=core;r=8:5892969-5893069;tl=RpFid1fPWgCVa7tG-11457638) | 5893019 | T | intergenic_variant | - | - |
| [8](https://www.ensembl.org/Salmo_trutta/Location/View?contigviewbottom=variation_feature_variation%3Dnormal;db=core;r=8:5892992-5893092;tl=RpFid1fPWgCVa7tG-11457638) | 5893042 | A | intergenic_variant | - | - |
| [8](https://www.ensembl.org/Salmo_trutta/Location/View?contigviewbottom=variation_feature_variation%3Dnormal;db=core;r=8:17757026-17757126;tl=RpFid1fPWgCVa7tG-11457638) | 17757076 | C | downstream_gene_variant | - | [ENSSTUG00000045820](https://www.ensembl.org/Salmo_trutta/Gene/Summary?db=core;g=ENSSTUG00000045820;tl=RpFid1fPWgCVa7tG-11457638) |
| [8](https://www.ensembl.org/Salmo_trutta/Location/View?contigviewbottom=variation_feature_variation%3Dnormal;db=core;r=8:25870842-25870942;tl=RpFid1fPWgCVa7tG-11457638) | 25870892 | G | intergenic_variant | - | - |
| [8](https://www.ensembl.org/Salmo_trutta/Location/View?contigviewbottom=variation_feature_variation%3Dnormal;db=core;r=8:37509034-37509134;tl=RpFid1fPWgCVa7tG-11457638) | 37509084 | G | intron_variant | plxnc1 | [ENSSTUG00000011483](https://www.ensembl.org/Salmo_trutta/Gene/Summary?db=core;g=ENSSTUG00000011483;tl=RpFid1fPWgCVa7tG-11457638) |
| [8](https://www.ensembl.org/Salmo_trutta/Location/View?contigviewbottom=variation_feature_variation%3Dnormal;db=core;r=8:37509034-37509134;tl=RpFid1fPWgCVa7tG-11457638) | 37509084 | G | downstream_gene_variant | - | [ENSSTUG00000011475](https://www.ensembl.org/Salmo_trutta/Gene/Summary?db=core;g=ENSSTUG00000011475;tl=RpFid1fPWgCVa7tG-11457638) |
| [8](https://www.ensembl.org/Salmo_trutta/Location/View?contigviewbottom=variation_feature_variation%3Dnormal;db=core;r=8:37509034-37509134;tl=RpFid1fPWgCVa7tG-11457638) | 37509084 | G | downstream_gene_variant | - | [ENSSTUG00000011476](https://www.ensembl.org/Salmo_trutta/Gene/Summary?db=core;g=ENSSTUG00000011476;tl=RpFid1fPWgCVa7tG-11457638) |
| [9](https://www.ensembl.org/Salmo_trutta/Location/View?contigviewbottom=variation_feature_variation%3Dnormal;db=core;r=9:2679729-2679829;tl=RpFid1fPWgCVa7tG-11457638) | 2679779 | A | intron_variant | grid2 | [ENSSTUG00000015841](https://www.ensembl.org/Salmo_trutta/Gene/Summary?db=core;g=ENSSTUG00000015841;tl=RpFid1fPWgCVa7tG-11457638) |
| [9](https://www.ensembl.org/Salmo_trutta/Location/View?contigviewbottom=variation_feature_variation%3Dnormal;db=core;r=9:6633486-6633586;tl=RpFid1fPWgCVa7tG-11457638) | 6633536 | C | intergenic_variant | - | - |
| [9](https://www.ensembl.org/Salmo_trutta/Location/View?contigviewbottom=variation_feature_variation%3Dnormal;db=core;r=9:16015854-16015954;tl=RpFid1fPWgCVa7tG-11457638) | 16015904 | G | intron_variant | rimbp2b | [ENSSTUG00000020210](https://www.ensembl.org/Salmo_trutta/Gene/Summary?db=core;g=ENSSTUG00000020210;tl=RpFid1fPWgCVa7tG-11457638) |
| [9](https://www.ensembl.org/Salmo_trutta/Location/View?contigviewbottom=variation_feature_variation%3Dnormal;db=core;r=9:16268481-16268581;tl=RpFid1fPWgCVa7tG-11457638) | 16268531 | T | intron_variant | TMEM132D | [ENSSTUG00000020332](https://www.ensembl.org/Salmo_trutta/Gene/Summary?db=core;g=ENSSTUG00000020332;tl=RpFid1fPWgCVa7tG-11457638) |
| [9](https://www.ensembl.org/Salmo_trutta/Location/View?contigviewbottom=variation_feature_variation%3Dnormal;db=core;r=9:23019093-23019193;tl=RpFid1fPWgCVa7tG-11457638) | 23019143 | T | intron_variant | iscua | [ENSSTUG00000031811](https://www.ensembl.org/Salmo_trutta/Gene/Summary?db=core;g=ENSSTUG00000031811;tl=RpFid1fPWgCVa7tG-11457638) |
| [9](https://www.ensembl.org/Salmo_trutta/Location/View?contigviewbottom=variation_feature_variation%3Dnormal;db=core;r=9:23019093-23019193;tl=RpFid1fPWgCVa7tG-11457638) | 23019143 | T | upstream_gene_variant | sart3 | [ENSSTUG00000031648](https://www.ensembl.org/Salmo_trutta/Gene/Summary?db=core;g=ENSSTUG00000031648;tl=RpFid1fPWgCVa7tG-11457638) |
| [9](https://www.ensembl.org/Salmo_trutta/Location/View?contigviewbottom=variation_feature_variation%3Dnormal;db=core;r=9:23019093-23019193;tl=RpFid1fPWgCVa7tG-11457638) | 23019143 | T | downstream_gene_variant | si:ch211-191d15.2 | [ENSSTUG00000031825](https://www.ensembl.org/Salmo_trutta/Gene/Summary?db=core;g=ENSSTUG00000031825;tl=RpFid1fPWgCVa7tG-11457638) |
| [9](https://www.ensembl.org/Salmo_trutta/Location/View?contigviewbottom=variation_feature_variation%3Dnormal;db=core;r=9:36087122-36087222;tl=RpFid1fPWgCVa7tG-11457638) | 36087172 | G | intron_variant | KCNT1 | [ENSSTUG00000021279](https://www.ensembl.org/Salmo_trutta/Gene/Summary?db=core;g=ENSSTUG00000021279;tl=RpFid1fPWgCVa7tG-11457638) |
| [11](https://www.ensembl.org/Salmo_trutta/Location/View?contigviewbottom=variation_feature_variation%3Dnormal;db=core;r=11:11920621-11920721;tl=RpFid1fPWgCVa7tG-11457638) | 11920671 | C | intron_variant | fbxw7 | [ENSSTUG00000035149](https://www.ensembl.org/Salmo_trutta/Gene/Summary?db=core;g=ENSSTUG00000035149;tl=RpFid1fPWgCVa7tG-11457638) |
| [12](https://www.ensembl.org/Salmo_trutta/Location/View?contigviewbottom=variation_feature_variation%3Dnormal;db=core;r=12:8421382-8421482;tl=RpFid1fPWgCVa7tG-11457638) | 8421432 | A | intron_variant | luzp2 | [ENSSTUG00000006120](https://www.ensembl.org/Salmo_trutta/Gene/Summary?db=core;g=ENSSTUG00000006120;tl=RpFid1fPWgCVa7tG-11457638) |
| [12](https://www.ensembl.org/Salmo_trutta/Location/View?contigviewbottom=variation_feature_variation%3Dnormal;db=core;r=12:10333655-10333755;tl=RpFid1fPWgCVa7tG-11457638) | 10333705 | A | intergenic_variant | - | - |
| [12](https://www.ensembl.org/Salmo_trutta/Location/View?contigviewbottom=variation_feature_variation%3Dnormal;db=core;r=12:50476237-50476337;tl=RpFid1fPWgCVa7tG-11457638) | 50476287 | C | intron_variant | rapgef1b | [ENSSTUG00000043677](https://www.ensembl.org/Salmo_trutta/Gene/Summary?db=core;g=ENSSTUG00000043677;tl=RpFid1fPWgCVa7tG-11457638) |
| [12](https://www.ensembl.org/Salmo_trutta/Location/View?contigviewbottom=variation_feature_variation%3Dnormal;db=core;r=12:51159517-51159617;tl=RpFid1fPWgCVa7tG-11457638) | 51159567 | A | downstream_gene_variant | sptan1 | [ENSSTUG00000046139](https://www.ensembl.org/Salmo_trutta/Gene/Summary?db=core;g=ENSSTUG00000046139;tl=RpFid1fPWgCVa7tG-11457638) |
| [12](https://www.ensembl.org/Salmo_trutta/Location/View?contigviewbottom=variation_feature_variation%3Dnormal;db=core;r=12:57916059-57916159;tl=RpFid1fPWgCVa7tG-11457638) | 57916109 | A | intergenic_variant | - | - |
| [12](https://www.ensembl.org/Salmo_trutta/Location/View?contigviewbottom=variation_feature_variation%3Dnormal;db=core;r=12:60177680-60177780;tl=RpFid1fPWgCVa7tG-11457638) | 60177730 | A | upstream_gene_variant | atg10 | [ENSSTUG00000005057](https://www.ensembl.org/Salmo_trutta/Gene/Summary?db=core;g=ENSSTUG00000005057;tl=RpFid1fPWgCVa7tG-11457638) |
| [12](https://www.ensembl.org/Salmo_trutta/Location/View?contigviewbottom=variation_feature_variation%3Dnormal;db=core;r=12:60177680-60177780;tl=RpFid1fPWgCVa7tG-11457638) | 60177730 | A | downstream_gene_variant | zcchc9 | [ENSSTUG00000005085](https://www.ensembl.org/Salmo_trutta/Gene/Summary?db=core;g=ENSSTUG00000005085;tl=RpFid1fPWgCVa7tG-11457638) |
| [12](https://www.ensembl.org/Salmo_trutta/Location/View?contigviewbottom=variation_feature_variation%3Dnormal;db=core;r=12:60177680-60177780;tl=RpFid1fPWgCVa7tG-11457638) | 60177730 | A | upstream_gene_variant | - | [ENSSTUG00000005062](https://www.ensembl.org/Salmo_trutta/Gene/Summary?db=core;g=ENSSTUG00000005062;tl=RpFid1fPWgCVa7tG-11457638) |
| [12](https://www.ensembl.org/Salmo_trutta/Location/View?contigviewbottom=variation_feature_variation%3Dnormal;db=core;r=12:61583040-61583140;tl=RpFid1fPWgCVa7tG-11457638) | 61583090 | C | intron_variant | lhfpl2a | [ENSSTUG00000007040](https://www.ensembl.org/Salmo_trutta/Gene/Summary?db=core;g=ENSSTUG00000007040;tl=RpFid1fPWgCVa7tG-11457638) |
| [12](https://www.ensembl.org/Salmo_trutta/Location/View?contigviewbottom=variation_feature_variation%3Dnormal;db=core;r=12:61738537-61738637;tl=RpFid1fPWgCVa7tG-11457638) | 61738587 | G | intron_variant | AP3B1 | [ENSSTUG00000007196](https://www.ensembl.org/Salmo_trutta/Gene/Summary?db=core;g=ENSSTUG00000007196;tl=RpFid1fPWgCVa7tG-11457638) |
| [12](https://www.ensembl.org/Salmo_trutta/Location/View?contigviewbottom=variation_feature_variation%3Dnormal;db=core;r=12:62169337-62169437;tl=RpFid1fPWgCVa7tG-11457638) | 62169387 | T | intron_variant | SV2C | [ENSSTUG00000010522](https://www.ensembl.org/Salmo_trutta/Gene/Summary?db=core;g=ENSSTUG00000010522;tl=RpFid1fPWgCVa7tG-11457638) |
| [12](https://www.ensembl.org/Salmo_trutta/Location/View?contigviewbottom=variation_feature_variation%3Dnormal;db=core;r=12:62900973-62901073;tl=RpFid1fPWgCVa7tG-11457638) | 62901023 | C | upstream_gene_variant | - | [ENSSTUG00000014154](https://www.ensembl.org/Salmo_trutta/Gene/Summary?db=core;g=ENSSTUG00000014154;tl=RpFid1fPWgCVa7tG-11457638) |
| [12](https://www.ensembl.org/Salmo_trutta/Location/View?contigviewbottom=variation_feature_variation%3Dnormal;db=core;r=12:73588620-73588720;tl=RpFid1fPWgCVa7tG-11457638) | 73588670 | T | intergenic_variant | - | - |
| [12](https://www.ensembl.org/Salmo_trutta/Location/View?contigviewbottom=variation_feature_variation%3Dnormal;db=core;r=12:75206556-75206656;tl=RpFid1fPWgCVa7tG-11457638) | 75206606 | G | intron_variant | si:dkey-28o19.1 | [ENSSTUG00000029971](https://www.ensembl.org/Salmo_trutta/Gene/Summary?db=core;g=ENSSTUG00000029971;tl=RpFid1fPWgCVa7tG-11457638) |
| [12](https://www.ensembl.org/Salmo_trutta/Location/View?contigviewbottom=variation_feature_variation%3Dnormal;db=core;r=12:79586870-79586970;tl=RpFid1fPWgCVa7tG-11457638) | 79586920 | G | intron_variant | ltbp3 | [ENSSTUG00000043277](https://www.ensembl.org/Salmo_trutta/Gene/Summary?db=core;g=ENSSTUG00000043277;tl=RpFid1fPWgCVa7tG-11457638) |
| [12](https://www.ensembl.org/Salmo_trutta/Location/View?contigviewbottom=variation_feature_variation%3Dnormal;db=core;r=12:81193424-81193524;tl=RpFid1fPWgCVa7tG-11457638) | 81193474 | A | missense_variant | phldb2a | [ENSSTUG00000042035](https://www.ensembl.org/Salmo_trutta/Gene/Summary?db=core;g=ENSSTUG00000042035;tl=RpFid1fPWgCVa7tG-11457638) |
| [12](https://www.ensembl.org/Salmo_trutta/Location/View?contigviewbottom=variation_feature_variation%3Dnormal;db=core;r=12:91164452-91164552;tl=RpFid1fPWgCVa7tG-11457638) | 91164502 | T | intron_variant | frem2a | [ENSSTUG00000014003](https://www.ensembl.org/Salmo_trutta/Gene/Summary?db=core;g=ENSSTUG00000014003;tl=RpFid1fPWgCVa7tG-11457638) |
| [13](https://www.ensembl.org/Salmo_trutta/Location/View?contigviewbottom=variation_feature_variation%3Dnormal;db=core;r=13:4620957-4621057;tl=RpFid1fPWgCVa7tG-11457638) | 4621007 | A | downstream_gene_variant | ccna2 | [ENSSTUG00000005315](https://www.ensembl.org/Salmo_trutta/Gene/Summary?db=core;g=ENSSTUG00000005315;tl=RpFid1fPWgCVa7tG-11457638) |
| [13](https://www.ensembl.org/Salmo_trutta/Location/View?contigviewbottom=variation_feature_variation%3Dnormal;db=core;r=13:11417721-11417821;tl=RpFid1fPWgCVa7tG-11457638) | 11417771 | T | intron_variant | - | [ENSSTUG00000029764](https://www.ensembl.org/Salmo_trutta/Gene/Summary?db=core;g=ENSSTUG00000029764;tl=RpFid1fPWgCVa7tG-11457638) |
| [13](https://www.ensembl.org/Salmo_trutta/Location/View?contigviewbottom=variation_feature_variation%3Dnormal;db=core;r=13:13503196-13503296;tl=RpFid1fPWgCVa7tG-11457638) | 13503246 | G | downstream_gene_variant | naa15a | [ENSSTUG00000033994](https://www.ensembl.org/Salmo_trutta/Gene/Summary?db=core;g=ENSSTUG00000033994;tl=RpFid1fPWgCVa7tG-11457638) |
| [13](https://www.ensembl.org/Salmo_trutta/Location/View?contigviewbottom=variation_feature_variation%3Dnormal;db=core;r=13:13503196-13503296;tl=RpFid1fPWgCVa7tG-11457638) | 13503246 | G | upstream_gene_variant | rab33ba | [ENSSTUG00000036352](https://www.ensembl.org/Salmo_trutta/Gene/Summary?db=core;g=ENSSTUG00000036352;tl=RpFid1fPWgCVa7tG-11457638) |
| [13](https://www.ensembl.org/Salmo_trutta/Location/View?contigviewbottom=variation_feature_variation%3Dnormal;db=core;r=13:13503196-13503296;tl=RpFid1fPWgCVa7tG-11457638) | 13503246 | G | intron_variant | ugl | [ENSSTUG00000036309](https://www.ensembl.org/Salmo_trutta/Gene/Summary?db=core;g=ENSSTUG00000036309;tl=RpFid1fPWgCVa7tG-11457638) |
| [13](https://www.ensembl.org/Salmo_trutta/Location/View?contigviewbottom=variation_feature_variation%3Dnormal;db=core;r=13:13503196-13503296;tl=RpFid1fPWgCVa7tG-11457638) | 13503246 | G | downstream_gene_variant | - | [ENSSTUG00000036306](https://www.ensembl.org/Salmo_trutta/Gene/Summary?db=core;g=ENSSTUG00000036306;tl=RpFid1fPWgCVa7tG-11457638) |
| [13](https://www.ensembl.org/Salmo_trutta/Location/View?contigviewbottom=variation_feature_variation%3Dnormal;db=core;r=13:13996886-13996986;tl=RpFid1fPWgCVa7tG-11457638) | 13996936 | T | intergenic_variant | - | - |
| [13](https://www.ensembl.org/Salmo_trutta/Location/View?contigviewbottom=variation_feature_variation%3Dnormal;db=core;r=13:13996896-13996996;tl=RpFid1fPWgCVa7tG-11457638) | 13996946 | C | intergenic_variant | - | - |
| [13](https://www.ensembl.org/Salmo_trutta/Location/View?contigviewbottom=variation_feature_variation%3Dnormal;db=core;r=13:17025554-17025654;tl=RpFid1fPWgCVa7tG-11457638) | 17025604 | T | splice_polypyrimidine_tract_variant, intron_variant | fbxw11a | [ENSSTUG00000043926](https://www.ensembl.org/Salmo_trutta/Gene/Summary?db=core;g=ENSSTUG00000043926;tl=RpFid1fPWgCVa7tG-11457638) |
| [13](https://www.ensembl.org/Salmo_trutta/Location/View?contigviewbottom=variation_feature_variation%3Dnormal;db=core;r=13:17025554-17025654;tl=RpFid1fPWgCVa7tG-11457638) | 17025604 | T | downstream_gene_variant | fgf18a | [ENSSTUG00000043988](https://www.ensembl.org/Salmo_trutta/Gene/Summary?db=core;g=ENSSTUG00000043988;tl=RpFid1fPWgCVa7tG-11457638) |
| [13](https://www.ensembl.org/Salmo_trutta/Location/View?contigviewbottom=variation_feature_variation%3Dnormal;db=core;r=13:23587455-23587555;tl=RpFid1fPWgCVa7tG-11457638) | 23587505 | G | intron_variant | aff2 | [ENSSTUG00000027163](https://www.ensembl.org/Salmo_trutta/Gene/Summary?db=core;g=ENSSTUG00000027163;tl=RpFid1fPWgCVa7tG-11457638) |
| [13](https://www.ensembl.org/Salmo_trutta/Location/View?contigviewbottom=variation_feature_variation%3Dnormal;db=core;r=13:23587463-23587563;tl=RpFid1fPWgCVa7tG-11457638) | 23587513 | A | intron_variant | aff2 | [ENSSTUG00000027163](https://www.ensembl.org/Salmo_trutta/Gene/Summary?db=core;g=ENSSTUG00000027163;tl=RpFid1fPWgCVa7tG-11457638) |
| [13](https://www.ensembl.org/Salmo_trutta/Location/View?contigviewbottom=variation_feature_variation%3Dnormal;db=core;r=13:29984559-29984659;tl=RpFid1fPWgCVa7tG-11457638) | 29984609 | T | downstream_gene_variant | eif4ebp3l | [ENSSTUG00000043166](https://www.ensembl.org/Salmo_trutta/Gene/Summary?db=core;g=ENSSTUG00000043166;tl=RpFid1fPWgCVa7tG-11457638) |
| [13](https://www.ensembl.org/Salmo_trutta/Location/View?contigviewbottom=variation_feature_variation%3Dnormal;db=core;r=13:30191190-30191290;tl=RpFid1fPWgCVa7tG-11457638) | 30191240 | C | upstream_gene_variant | rmnd5b | [ENSSTUG00000045442](https://www.ensembl.org/Salmo_trutta/Gene/Summary?db=core;g=ENSSTUG00000045442;tl=RpFid1fPWgCVa7tG-11457638) |
| [13](https://www.ensembl.org/Salmo_trutta/Location/View?contigviewbottom=variation_feature_variation%3Dnormal;db=core;r=13:30191190-30191290;tl=RpFid1fPWgCVa7tG-11457638) | 30191240 | C | missense_variant | - | [ENSSTUG00000045434](https://www.ensembl.org/Salmo_trutta/Gene/Summary?db=core;g=ENSSTUG00000045434;tl=RpFid1fPWgCVa7tG-11457638) |
| [13](https://www.ensembl.org/Salmo_trutta/Location/View?contigviewbottom=variation_feature_variation%3Dnormal;db=core;r=13:30191212-30191312;tl=RpFid1fPWgCVa7tG-11457638) | 30191262 | G | upstream_gene_variant | rmnd5b | [ENSSTUG00000045442](https://www.ensembl.org/Salmo_trutta/Gene/Summary?db=core;g=ENSSTUG00000045442;tl=RpFid1fPWgCVa7tG-11457638) |
| [13](https://www.ensembl.org/Salmo_trutta/Location/View?contigviewbottom=variation_feature_variation%3Dnormal;db=core;r=13:30191212-30191312;tl=RpFid1fPWgCVa7tG-11457638) | 30191262 | G | synonymous_variant | - | [ENSSTUG00000045434](https://www.ensembl.org/Salmo_trutta/Gene/Summary?db=core;g=ENSSTUG00000045434;tl=RpFid1fPWgCVa7tG-11457638) |
| [13](https://www.ensembl.org/Salmo_trutta/Location/View?contigviewbottom=variation_feature_variation%3Dnormal;db=core;r=13:30191221-30191321;tl=RpFid1fPWgCVa7tG-11457638) | 30191271 | T | upstream_gene_variant | rmnd5b | [ENSSTUG00000045442](https://www.ensembl.org/Salmo_trutta/Gene/Summary?db=core;g=ENSSTUG00000045442;tl=RpFid1fPWgCVa7tG-11457638) |
| [13](https://www.ensembl.org/Salmo_trutta/Location/View?contigviewbottom=variation_feature_variation%3Dnormal;db=core;r=13:30191221-30191321;tl=RpFid1fPWgCVa7tG-11457638) | 30191271 | T | synonymous_variant | - | [ENSSTUG00000045434](https://www.ensembl.org/Salmo_trutta/Gene/Summary?db=core;g=ENSSTUG00000045434;tl=RpFid1fPWgCVa7tG-11457638) |
| [13](https://www.ensembl.org/Salmo_trutta/Location/View?contigviewbottom=variation_feature_variation%3Dnormal;db=core;r=13:30413585-30413685;tl=RpFid1fPWgCVa7tG-11457638) | 30413635 | T | downstream_gene_variant | afap1l1a | [ENSSTUG00000045558](https://www.ensembl.org/Salmo_trutta/Gene/Summary?db=core;g=ENSSTUG00000045558;tl=RpFid1fPWgCVa7tG-11457638) |
| [13](https://www.ensembl.org/Salmo_trutta/Location/View?contigviewbottom=variation_feature_variation%3Dnormal;db=core;r=13:30413585-30413685;tl=RpFid1fPWgCVa7tG-11457638) | 30413635 | T | downstream_gene_variant | gabrp | [ENSSTUG00000045548](https://www.ensembl.org/Salmo_trutta/Gene/Summary?db=core;g=ENSSTUG00000045548;tl=RpFid1fPWgCVa7tG-11457638) |
| [13](https://www.ensembl.org/Salmo_trutta/Location/View?contigviewbottom=variation_feature_variation%3Dnormal;db=core;r=13:32506765-32506865;tl=RpFid1fPWgCVa7tG-11457638) | 32506815 | T | splice_region_variant, splice_polypyrimidine_tract_variant,intron_variant | frmpd3 | [ENSSTUG00000006720](https://www.ensembl.org/Salmo_trutta/Gene/Summary?db=core;g=ENSSTUG00000006720;tl=RpFid1fPWgCVa7tG-11457638) |
| [13](https://www.ensembl.org/Salmo_trutta/Location/View?contigviewbottom=variation_feature_variation%3Dnormal;db=core;r=13:55266743-55266843;tl=RpFid1fPWgCVa7tG-11457638) | 55266793 | A | intergenic_variant | - | - |
| [13](https://www.ensembl.org/Salmo_trutta/Location/View?contigviewbottom=variation_feature_variation%3Dnormal;db=core;r=13:56999140-56999240;tl=RpFid1fPWgCVa7tG-11457638) | 56999190 | C | upstream_gene_variant | zgc:152863 | [ENSSTUG00000039713](https://www.ensembl.org/Salmo_trutta/Gene/Summary?db=core;g=ENSSTUG00000039713;tl=RpFid1fPWgCVa7tG-11457638) |
| [13](https://www.ensembl.org/Salmo_trutta/Location/View?contigviewbottom=variation_feature_variation%3Dnormal;db=core;r=13:56999140-56999240;tl=RpFid1fPWgCVa7tG-11457638) | 56999190 | C | 3_prime_UTR_variant | - | [ENSSTUG00000039724](https://www.ensembl.org/Salmo_trutta/Gene/Summary?db=core;g=ENSSTUG00000039724;tl=RpFid1fPWgCVa7tG-11457638) |
| [13](https://www.ensembl.org/Salmo_trutta/Location/View?contigviewbottom=variation_feature_variation%3Dnormal;db=core;r=13:62913370-62913470;tl=RpFid1fPWgCVa7tG-11457638) | 62913420 | C | intron_variant | bcas3 | [ENSSTUG00000046908](https://www.ensembl.org/Salmo_trutta/Gene/Summary?db=core;g=ENSSTUG00000046908;tl=RpFid1fPWgCVa7tG-11457638) |
| [13](https://www.ensembl.org/Salmo_trutta/Location/View?contigviewbottom=variation_feature_variation%3Dnormal;db=core;r=13:62913398-62913498;tl=RpFid1fPWgCVa7tG-11457638) | 62913448 | C | intron_variant | bcas3 | [ENSSTUG00000046908](https://www.ensembl.org/Salmo_trutta/Gene/Summary?db=core;g=ENSSTUG00000046908;tl=RpFid1fPWgCVa7tG-11457638) |
| [14](https://www.ensembl.org/Salmo_trutta/Location/View?contigviewbottom=variation_feature_variation%3Dnormal;db=core;r=14:2052450-2052550;tl=RpFid1fPWgCVa7tG-11457638) | 2052500 | A | intron_variant | slmapa | [ENSSTUG00000001585](https://www.ensembl.org/Salmo_trutta/Gene/Summary?db=core;g=ENSSTUG00000001585;tl=RpFid1fPWgCVa7tG-11457638) |
| [14](https://www.ensembl.org/Salmo_trutta/Location/View?contigviewbottom=variation_feature_variation%3Dnormal;db=core;r=14:4262023-4262123;tl=RpFid1fPWgCVa7tG-11457638) | 4262073 | C | intergenic_variant | - | - |
| [14](https://www.ensembl.org/Salmo_trutta/Location/View?contigviewbottom=variation_feature_variation%3Dnormal;db=core;r=14:4262027-4262127;tl=RpFid1fPWgCVa7tG-11457638) | 4262077 | T | intergenic_variant | - | - |
| [14](https://www.ensembl.org/Salmo_trutta/Location/View?contigviewbottom=variation_feature_variation%3Dnormal;db=core;r=14:40758261-40758361;tl=RpFid1fPWgCVa7tG-11457638) | 40758311 | G | downstream_gene_variant | SRGAP3 | [ENSSTUG00000010018](https://www.ensembl.org/Salmo_trutta/Gene/Summary?db=core;g=ENSSTUG00000010018;tl=RpFid1fPWgCVa7tG-11457638) |
| [14](https://www.ensembl.org/Salmo_trutta/Location/View?contigviewbottom=variation_feature_variation%3Dnormal;db=core;r=14:58568545-58568645;tl=RpFid1fPWgCVa7tG-11457638) | 58568595 | A | upstream_gene_variant | c14h2orf73 | [ENSSTUG00000008369](https://www.ensembl.org/Salmo_trutta/Gene/Summary?db=core;g=ENSSTUG00000008369;tl=RpFid1fPWgCVa7tG-11457638) |
| [14](https://www.ensembl.org/Salmo_trutta/Location/View?contigviewbottom=variation_feature_variation%3Dnormal;db=core;r=14:58846143-58846243;tl=RpFid1fPWgCVa7tG-11457638) | 58846193 | T | intergenic_variant | - | - |
| [14](https://www.ensembl.org/Salmo_trutta/Location/View?contigviewbottom=variation_feature_variation%3Dnormal;db=core;r=14:58846146-58846246;tl=RpFid1fPWgCVa7tG-11457638) | 58846196 | A | intergenic_variant | - | - |
| [14](https://www.ensembl.org/Salmo_trutta/Location/View?contigviewbottom=variation_feature_variation%3Dnormal;db=core;r=14:58902369-58902469;tl=RpFid1fPWgCVa7tG-11457638) | 58902419 | G | intron_variant | si:dkey-49c17.4 | [ENSSTUG00000014356](https://www.ensembl.org/Salmo_trutta/Gene/Summary?db=core;g=ENSSTUG00000014356;tl=RpFid1fPWgCVa7tG-11457638) |
| [14](https://www.ensembl.org/Salmo_trutta/Location/View?contigviewbottom=variation_feature_variation%3Dnormal;db=core;r=14:77878578-77878678;tl=RpFid1fPWgCVa7tG-11457638) | 77878628 | A | upstream_gene_variant | - | [ENSSTUG00000036208](https://www.ensembl.org/Salmo_trutta/Gene/Summary?db=core;g=ENSSTUG00000036208;tl=RpFid1fPWgCVa7tG-11457638) |
| [14](https://www.ensembl.org/Salmo_trutta/Location/View?contigviewbottom=variation_feature_variation%3Dnormal;db=core;r=14:77878578-77878678;tl=RpFid1fPWgCVa7tG-11457638) | 77878628 | A | intron_variant | - | [ENSSTUG00000036135](https://www.ensembl.org/Salmo_trutta/Gene/Summary?db=core;g=ENSSTUG00000036135;tl=RpFid1fPWgCVa7tG-11457638) |
| [14](https://www.ensembl.org/Salmo_trutta/Location/View?contigviewbottom=variation_feature_variation%3Dnormal;db=core;r=14:77878578-77878678;tl=RpFid1fPWgCVa7tG-11457638) | 77878628 | A | upstream_gene_variant | - | [ENSSTUG00000036220](https://www.ensembl.org/Salmo_trutta/Gene/Summary?db=core;g=ENSSTUG00000036220;tl=RpFid1fPWgCVa7tG-11457638) |
| [14](https://www.ensembl.org/Salmo_trutta/Location/View?contigviewbottom=variation_feature_variation%3Dnormal;db=core;r=14:77878594-77878694;tl=RpFid1fPWgCVa7tG-11457638) | 77878644 | T | upstream_gene_variant | - | [ENSSTUG00000036208](https://www.ensembl.org/Salmo_trutta/Gene/Summary?db=core;g=ENSSTUG00000036208;tl=RpFid1fPWgCVa7tG-11457638) |
| [14](https://www.ensembl.org/Salmo_trutta/Location/View?contigviewbottom=variation_feature_variation%3Dnormal;db=core;r=14:77878594-77878694;tl=RpFid1fPWgCVa7tG-11457638) | 77878644 | T | upstream_gene_variant | - | [ENSSTUG00000036220](https://www.ensembl.org/Salmo_trutta/Gene/Summary?db=core;g=ENSSTUG00000036220;tl=RpFid1fPWgCVa7tG-11457638) |
| [14](https://www.ensembl.org/Salmo_trutta/Location/View?contigviewbottom=variation_feature_variation%3Dnormal;db=core;r=14:77878594-77878694;tl=RpFid1fPWgCVa7tG-11457638) | 77878644 | T | intron_variant | - | [ENSSTUG00000036135](https://www.ensembl.org/Salmo_trutta/Gene/Summary?db=core;g=ENSSTUG00000036135;tl=RpFid1fPWgCVa7tG-11457638) |
| [15](https://www.ensembl.org/Salmo_trutta/Location/View?contigviewbottom=variation_feature_variation%3Dnormal;db=core;r=15:9702542-9702642;tl=RpFid1fPWgCVa7tG-11457638) | 9702592 | A | intron_variant | p2rx1 | [ENSSTUG00000017171](https://www.ensembl.org/Salmo_trutta/Gene/Summary?db=core;g=ENSSTUG00000017171;tl=RpFid1fPWgCVa7tG-11457638) |
| [15](https://www.ensembl.org/Salmo_trutta/Location/View?contigviewbottom=variation_feature_variation%3Dnormal;db=core;r=15:11909888-11909988;tl=RpFid1fPWgCVa7tG-11457638) | 11909938 | G | intron_variant | ubtd2 | [ENSSTUG00000011499](https://www.ensembl.org/Salmo_trutta/Gene/Summary?db=core;g=ENSSTUG00000011499;tl=RpFid1fPWgCVa7tG-11457638) |
| [15](https://www.ensembl.org/Salmo_trutta/Location/View?contigviewbottom=variation_feature_variation%3Dnormal;db=core;r=15:11909889-11909989;tl=RpFid1fPWgCVa7tG-11457638) | 11909939 | A | intron_variant | ubtd2 | [ENSSTUG00000011499](https://www.ensembl.org/Salmo_trutta/Gene/Summary?db=core;g=ENSSTUG00000011499;tl=RpFid1fPWgCVa7tG-11457638) |
| [15](https://www.ensembl.org/Salmo_trutta/Location/View?contigviewbottom=variation_feature_variation%3Dnormal;db=core;r=15:13634808-13634908;tl=RpFid1fPWgCVa7tG-11457638) | 13634858 | A | intron_variant | NR3C1 | [ENSSTUG00000016475](https://www.ensembl.org/Salmo_trutta/Gene/Summary?db=core;g=ENSSTUG00000016475;tl=RpFid1fPWgCVa7tG-11457638) |
| [15](https://www.ensembl.org/Salmo_trutta/Location/View?contigviewbottom=variation_feature_variation%3Dnormal;db=core;r=15:22952408-22952508;tl=RpFid1fPWgCVa7tG-11457638) | 22952458 | G | intron_variant | exoc3l2a | [ENSSTUG00000011550](https://www.ensembl.org/Salmo_trutta/Gene/Summary?db=core;g=ENSSTUG00000011550;tl=RpFid1fPWgCVa7tG-11457638) |
| [15](https://www.ensembl.org/Salmo_trutta/Location/View?contigviewbottom=variation_feature_variation%3Dnormal;db=core;r=15:30182283-30182383;tl=RpFid1fPWgCVa7tG-11457638) | 30182333 | C | upstream_gene_variant | CBL | [ENSSTUG00000002617](https://www.ensembl.org/Salmo_trutta/Gene/Summary?db=core;g=ENSSTUG00000002617;tl=RpFid1fPWgCVa7tG-11457638) |
| [15](https://www.ensembl.org/Salmo_trutta/Location/View?contigviewbottom=variation_feature_variation%3Dnormal;db=core;r=15:30182283-30182383;tl=RpFid1fPWgCVa7tG-11457638) | 30182333 | C | upstream_gene_variant | polr2j | [ENSSTUG00000002891](https://www.ensembl.org/Salmo_trutta/Gene/Summary?db=core;g=ENSSTUG00000002891;tl=RpFid1fPWgCVa7tG-11457638) |
| [15](https://www.ensembl.org/Salmo_trutta/Location/View?contigviewbottom=variation_feature_variation%3Dnormal;db=core;r=15:54277328-54277428;tl=RpFid1fPWgCVa7tG-11457638) | 54277378 | T | upstream_gene_variant | apc | [ENSSTUG00000036593](https://www.ensembl.org/Salmo_trutta/Gene/Summary?db=core;g=ENSSTUG00000036593;tl=RpFid1fPWgCVa7tG-11457638) |
| [15](https://www.ensembl.org/Salmo_trutta/Location/View?contigviewbottom=variation_feature_variation%3Dnormal;db=core;r=15:54277350-54277450;tl=RpFid1fPWgCVa7tG-11457638) | 54277400 | T | upstream_gene_variant | apc | [ENSSTUG00000036593](https://www.ensembl.org/Salmo_trutta/Gene/Summary?db=core;g=ENSSTUG00000036593;tl=RpFid1fPWgCVa7tG-11457638) |
| [15](https://www.ensembl.org/Salmo_trutta/Location/View?contigviewbottom=variation_feature_variation%3Dnormal;db=core;r=15:64786852-64786952;tl=RpFid1fPWgCVa7tG-11457638) | 64786902 | C | intergenic_variant | - | - |
| [16](https://www.ensembl.org/Salmo_trutta/Location/View?contigviewbottom=variation_feature_variation%3Dnormal;db=core;r=16:13815854-13815954;tl=RpFid1fPWgCVa7tG-11457638) | 13815904 | C | synonymous_variant | helz2a | [ENSSTUG00000031078](https://www.ensembl.org/Salmo_trutta/Gene/Summary?db=core;g=ENSSTUG00000031078;tl=RpFid1fPWgCVa7tG-11457638) |
| [16](https://www.ensembl.org/Salmo_trutta/Location/View?contigviewbottom=variation_feature_variation%3Dnormal;db=core;r=16:13815854-13815954;tl=RpFid1fPWgCVa7tG-11457638) | 13815904 | C | intron_variant | stmn3 | [ENSSTUG00000031072](https://www.ensembl.org/Salmo_trutta/Gene/Summary?db=core;g=ENSSTUG00000031072;tl=RpFid1fPWgCVa7tG-11457638) |
| [16](https://www.ensembl.org/Salmo_trutta/Location/View?contigviewbottom=variation_feature_variation%3Dnormal;db=core;r=16:16206254-16206354;tl=RpFid1fPWgCVa7tG-11457638) | 16206304 | G | downstream_gene_variant | tpra1 | [ENSSTUG00000046616](https://www.ensembl.org/Salmo_trutta/Gene/Summary?db=core;g=ENSSTUG00000046616;tl=RpFid1fPWgCVa7tG-11457638) |
| [16](https://www.ensembl.org/Salmo_trutta/Location/View?contigviewbottom=variation_feature_variation%3Dnormal;db=core;r=16:17063212-17063312;tl=RpFid1fPWgCVa7tG-11457638) | 17063262 | T | downstream_gene_variant | MINAR1 | [ENSSTUG00000047472](https://www.ensembl.org/Salmo_trutta/Gene/Summary?db=core;g=ENSSTUG00000047472;tl=RpFid1fPWgCVa7tG-11457638) |
| [16](https://www.ensembl.org/Salmo_trutta/Location/View?contigviewbottom=variation_feature_variation%3Dnormal;db=core;r=16:17063212-17063312;tl=RpFid1fPWgCVa7tG-11457638) | 17063262 | T | upstream_gene_variant | - | [ENSSTUG00000047451](https://www.ensembl.org/Salmo_trutta/Gene/Summary?db=core;g=ENSSTUG00000047451;tl=RpFid1fPWgCVa7tG-11457638) |
| [16](https://www.ensembl.org/Salmo_trutta/Location/View?contigviewbottom=variation_feature_variation%3Dnormal;db=core;r=16:19609427-19609527;tl=RpFid1fPWgCVa7tG-11457638) | 19609477 | A | intron_variant | cacna1da | [ENSSTUG00000022566](https://www.ensembl.org/Salmo_trutta/Gene/Summary?db=core;g=ENSSTUG00000022566;tl=RpFid1fPWgCVa7tG-11457638) |
| [16](https://www.ensembl.org/Salmo_trutta/Location/View?contigviewbottom=variation_feature_variation%3Dnormal;db=core;r=16:20249993-20250093;tl=RpFid1fPWgCVa7tG-11457638) | 20250043 | C | downstream_gene_variant | atxn7l2a | [ENSSTUG00000032994](https://www.ensembl.org/Salmo_trutta/Gene/Summary?db=core;g=ENSSTUG00000032994;tl=RpFid1fPWgCVa7tG-11457638) |
| [16](https://www.ensembl.org/Salmo_trutta/Location/View?contigviewbottom=variation_feature_variation%3Dnormal;db=core;r=16:20249993-20250093;tl=RpFid1fPWgCVa7tG-11457638) | 20250043 | C | synonymous_variant | cyb561d1 | [ENSSTUG00000033002](https://www.ensembl.org/Salmo_trutta/Gene/Summary?db=core;g=ENSSTUG00000033002;tl=RpFid1fPWgCVa7tG-11457638) |
| [16](https://www.ensembl.org/Salmo_trutta/Location/View?contigviewbottom=variation_feature_variation%3Dnormal;db=core;r=16:20774278-20774378;tl=RpFid1fPWgCVa7tG-11457638) | 20774328 | A | upstream_gene_variant | arl8a | [ENSSTUG00000014640](https://www.ensembl.org/Salmo_trutta/Gene/Summary?db=core;g=ENSSTUG00000014640;tl=RpFid1fPWgCVa7tG-11457638) |
| [16](https://www.ensembl.org/Salmo_trutta/Location/View?contigviewbottom=variation_feature_variation%3Dnormal;db=core;r=16:20774278-20774378;tl=RpFid1fPWgCVa7tG-11457638) | 20774328 | A | splice_polypyrimidine_tract_variant, intron_variant | zgc:162255 | [ENSSTUG00000014623](https://www.ensembl.org/Salmo_trutta/Gene/Summary?db=core;g=ENSSTUG00000014623;tl=RpFid1fPWgCVa7tG-11457638) |
| [16](https://www.ensembl.org/Salmo_trutta/Location/View?contigviewbottom=variation_feature_variation%3Dnormal;db=core;r=16:20774278-20774378;tl=RpFid1fPWgCVa7tG-11457638) | 20774328 | A | upstream_gene_variant | znf76 | [ENSSTUG00000014594](https://www.ensembl.org/Salmo_trutta/Gene/Summary?db=core;g=ENSSTUG00000014594;tl=RpFid1fPWgCVa7tG-11457638) |
| [16](https://www.ensembl.org/Salmo_trutta/Location/View?contigviewbottom=variation_feature_variation%3Dnormal;db=core;r=16:26400178-26400278;tl=RpFid1fPWgCVa7tG-11457638) | 26400228 | C | intergenic_variant | - | - |
| [16](https://www.ensembl.org/Salmo_trutta/Location/View?contigviewbottom=variation_feature_variation%3Dnormal;db=core;r=16:41394892-41394992;tl=RpFid1fPWgCVa7tG-11457638) | 41394942 | G | intron_variant | ptprga | [ENSSTUG00000009135](https://www.ensembl.org/Salmo_trutta/Gene/Summary?db=core;g=ENSSTUG00000009135;tl=RpFid1fPWgCVa7tG-11457638) |
| [16](https://www.ensembl.org/Salmo_trutta/Location/View?contigviewbottom=variation_feature_variation%3Dnormal;db=core;r=16:50191753-50191853;tl=RpFid1fPWgCVa7tG-11457638) | 50191803 | C | intron_variant | sema3fa | [ENSSTUG00000049371](https://www.ensembl.org/Salmo_trutta/Gene/Summary?db=core;g=ENSSTUG00000049371;tl=RpFid1fPWgCVa7tG-11457638) |
| [16](https://www.ensembl.org/Salmo_trutta/Location/View?contigviewbottom=variation_feature_variation%3Dnormal;db=core;r=16:59376084-59376184;tl=RpFid1fPWgCVa7tG-11457638) | 59376134 | A | intron_variant | PTPRT | [ENSSTUG00000039419](https://www.ensembl.org/Salmo_trutta/Gene/Summary?db=core;g=ENSSTUG00000039419;tl=RpFid1fPWgCVa7tG-11457638) |
| [17](https://www.ensembl.org/Salmo_trutta/Location/View?contigviewbottom=variation_feature_variation%3Dnormal;db=core;r=17:16639178-16639278;tl=RpFid1fPWgCVa7tG-11457638) | 16639228 | C | intron_variant | si:zfos-588f8.1 | [ENSSTUG00000015770](https://www.ensembl.org/Salmo_trutta/Gene/Summary?db=core;g=ENSSTUG00000015770;tl=RpFid1fPWgCVa7tG-11457638) |
| [17](https://www.ensembl.org/Salmo_trutta/Location/View?contigviewbottom=variation_feature_variation%3Dnormal;db=core;r=17:34832240-34832340;tl=RpFid1fPWgCVa7tG-11457638) | 34832290 | C | upstream_gene_variant | prr5a | [ENSSTUG00000034832](https://www.ensembl.org/Salmo_trutta/Gene/Summary?db=core;g=ENSSTUG00000034832;tl=RpFid1fPWgCVa7tG-11457638) |
| [17](https://www.ensembl.org/Salmo_trutta/Location/View?contigviewbottom=variation_feature_variation%3Dnormal;db=core;r=17:34832240-34832340;tl=RpFid1fPWgCVa7tG-11457638) | 34832290 | C | upstream_gene_variant | rad52 | [ENSSTUG00000034865](https://www.ensembl.org/Salmo_trutta/Gene/Summary?db=core;g=ENSSTUG00000034865;tl=RpFid1fPWgCVa7tG-11457638) |
| [17](https://www.ensembl.org/Salmo_trutta/Location/View?contigviewbottom=variation_feature_variation%3Dnormal;db=core;r=17:34832240-34832340;tl=RpFid1fPWgCVa7tG-11457638) | 34832290 | C | downstream_gene_variant | wnk1a | [ENSSTUG00000034878](https://www.ensembl.org/Salmo_trutta/Gene/Summary?db=core;g=ENSSTUG00000034878;tl=RpFid1fPWgCVa7tG-11457638) |
| [17](https://www.ensembl.org/Salmo_trutta/Location/View?contigviewbottom=variation_feature_variation%3Dnormal;db=core;r=17:39958814-39958914;tl=RpFid1fPWgCVa7tG-11457638) | 39958864 | A | intron_variant | ppfibp1b | [ENSSTUG00000050051](https://www.ensembl.org/Salmo_trutta/Gene/Summary?db=core;g=ENSSTUG00000050051;tl=RpFid1fPWgCVa7tG-11457638) |
| [17](https://www.ensembl.org/Salmo_trutta/Location/View?contigviewbottom=variation_feature_variation%3Dnormal;db=core;r=17:40679428-40679528;tl=RpFid1fPWgCVa7tG-11457638) | 40679478 | G | synonymous_variant | mtss1la | [ENSSTUG00000002460](https://www.ensembl.org/Salmo_trutta/Gene/Summary?db=core;g=ENSSTUG00000002460;tl=RpFid1fPWgCVa7tG-11457638) |
| [18](https://www.ensembl.org/Salmo_trutta/Location/View?contigviewbottom=variation_feature_variation%3Dnormal;db=core;r=18:24277747-24277847;tl=RpFid1fPWgCVa7tG-11457638) | 24277797 | A | intron_variant | ADGRA1 | [ENSSTUG00000009543](https://www.ensembl.org/Salmo_trutta/Gene/Summary?db=core;g=ENSSTUG00000009543;tl=RpFid1fPWgCVa7tG-11457638) |
| [18](https://www.ensembl.org/Salmo_trutta/Location/View?contigviewbottom=variation_feature_variation%3Dnormal;db=core;r=18:48577238-48577338;tl=RpFid1fPWgCVa7tG-11457638) | 48577288 | G | intron_variant | DST | [ENSSTUG00000046156](https://www.ensembl.org/Salmo_trutta/Gene/Summary?db=core;g=ENSSTUG00000046156;tl=RpFid1fPWgCVa7tG-11457638) |
| [19](https://www.ensembl.org/Salmo_trutta/Location/View?contigviewbottom=variation_feature_variation%3Dnormal;db=core;r=19:2172663-2172763;tl=RpFid1fPWgCVa7tG-11457638) | 2172713 | A | intergenic_variant | - | - |
| [19](https://www.ensembl.org/Salmo_trutta/Location/View?contigviewbottom=variation_feature_variation%3Dnormal;db=core;r=19:8816485-8816585;tl=RpFid1fPWgCVa7tG-11457638) | 8816535 | G | downstream_gene_variant | adrb2b | [ENSSTUG00000004048](https://www.ensembl.org/Salmo_trutta/Gene/Summary?db=core;g=ENSSTUG00000004048;tl=RpFid1fPWgCVa7tG-11457638) |
| [19](https://www.ensembl.org/Salmo_trutta/Location/View?contigviewbottom=variation_feature_variation%3Dnormal;db=core;r=19:26342353-26342453;tl=RpFid1fPWgCVa7tG-11457638) | 26342403 | A | downstream_gene_variant | nup88 | [ENSSTUG00000017702](https://www.ensembl.org/Salmo_trutta/Gene/Summary?db=core;g=ENSSTUG00000017702;tl=RpFid1fPWgCVa7tG-11457638) |
| [19](https://www.ensembl.org/Salmo_trutta/Location/View?contigviewbottom=variation_feature_variation%3Dnormal;db=core;r=19:26342353-26342453;tl=RpFid1fPWgCVa7tG-11457638) | 26342403 | A | downstream_gene_variant | rabep1 | [ENSSTUG00000018241](https://www.ensembl.org/Salmo_trutta/Gene/Summary?db=core;g=ENSSTUG00000018241;tl=RpFid1fPWgCVa7tG-11457638) |
| [19](https://www.ensembl.org/Salmo_trutta/Location/View?contigviewbottom=variation_feature_variation%3Dnormal;db=core;r=19:26441259-26441359;tl=RpFid1fPWgCVa7tG-11457638) | 26441309 | G | upstream_gene_variant | - | [ENSSTUG00000019672](https://www.ensembl.org/Salmo_trutta/Gene/Summary?db=core;g=ENSSTUG00000019672;tl=RpFid1fPWgCVa7tG-11457638) |
| [19](https://www.ensembl.org/Salmo_trutta/Location/View?contigviewbottom=variation_feature_variation%3Dnormal;db=core;r=19:37251960-37252060;tl=RpFid1fPWgCVa7tG-11457638) | 37252010 | T | intron_variant | tenm1 | [ENSSTUG00000032523](https://www.ensembl.org/Salmo_trutta/Gene/Summary?db=core;g=ENSSTUG00000032523;tl=RpFid1fPWgCVa7tG-11457638) |
| [20](https://www.ensembl.org/Salmo_trutta/Location/View?contigviewbottom=variation_feature_variation%3Dnormal;db=core;r=20:486386-486486;tl=RpFid1fPWgCVa7tG-11457638) | 486436 | C | intron_variant | dip2a | [ENSSTUG00000011791](https://www.ensembl.org/Salmo_trutta/Gene/Summary?db=core;g=ENSSTUG00000011791;tl=RpFid1fPWgCVa7tG-11457638) |
| [21](https://www.ensembl.org/Salmo_trutta/Location/View?contigviewbottom=variation_feature_variation%3Dnormal;db=core;r=21:282421-282521;tl=RpFid1fPWgCVa7tG-11457638) | 282471 | A | intron_variant | asap1a | [ENSSTUG00000028518](https://www.ensembl.org/Salmo_trutta/Gene/Summary?db=core;g=ENSSTUG00000028518;tl=RpFid1fPWgCVa7tG-11457638) |
| [21](https://www.ensembl.org/Salmo_trutta/Location/View?contigviewbottom=variation_feature_variation%3Dnormal;db=core;r=21:7025548-7025648;tl=RpFid1fPWgCVa7tG-11457638) | 7025598 | C | intergenic_variant | - | - |
| [21](https://www.ensembl.org/Salmo_trutta/Location/View?contigviewbottom=variation_feature_variation%3Dnormal;db=core;r=21:9982438-9982538;tl=RpFid1fPWgCVa7tG-11457638) | 9982488 | T | intron_variant | brinp3b | [ENSSTUG00000047899](https://www.ensembl.org/Salmo_trutta/Gene/Summary?db=core;g=ENSSTUG00000047899;tl=RpFid1fPWgCVa7tG-11457638) |
| [21](https://www.ensembl.org/Salmo_trutta/Location/View?contigviewbottom=variation_feature_variation%3Dnormal;db=core;r=21:16119318-16119418;tl=RpFid1fPWgCVa7tG-11457638) | 16119368 | T | intron_variant | dpydb | [ENSSTUG00000029381](https://www.ensembl.org/Salmo_trutta/Gene/Summary?db=core;g=ENSSTUG00000029381;tl=RpFid1fPWgCVa7tG-11457638) |
| [21](https://www.ensembl.org/Salmo_trutta/Location/View?contigviewbottom=variation_feature_variation%3Dnormal;db=core;r=21:17843596-17843696;tl=RpFid1fPWgCVa7tG-11457638) | 17843646 | A | intron_variant | gigyf2 | [ENSSTUG00000025011](https://www.ensembl.org/Salmo_trutta/Gene/Summary?db=core;g=ENSSTUG00000025011;tl=RpFid1fPWgCVa7tG-11457638) |
| [21](https://www.ensembl.org/Salmo_trutta/Location/View?contigviewbottom=variation_feature_variation%3Dnormal;db=core;r=21:31573392-31573492;tl=RpFid1fPWgCVa7tG-11457638) | 31573442 | A | downstream_gene_variant | dtna | [ENSSTUG00000006673](https://www.ensembl.org/Salmo_trutta/Gene/Summary?db=core;g=ENSSTUG00000006673;tl=RpFid1fPWgCVa7tG-11457638) |
| [22](https://www.ensembl.org/Salmo_trutta/Location/View?contigviewbottom=variation_feature_variation%3Dnormal;db=core;r=22:8578003-8578103;tl=RpFid1fPWgCVa7tG-11457638) | 8578053 | A | downstream_gene_variant | - | [ENSSTUG00000007000](https://www.ensembl.org/Salmo_trutta/Gene/Summary?db=core;g=ENSSTUG00000007000;tl=RpFid1fPWgCVa7tG-11457638) |
| [22](https://www.ensembl.org/Salmo_trutta/Location/View?contigviewbottom=variation_feature_variation%3Dnormal;db=core;r=22:8578003-8578103;tl=RpFid1fPWgCVa7tG-11457638) | 8578053 | A | upstream_gene_variant | - | [ENSSTUG00000006997](https://www.ensembl.org/Salmo_trutta/Gene/Summary?db=core;g=ENSSTUG00000006997;tl=RpFid1fPWgCVa7tG-11457638) |
| [22](https://www.ensembl.org/Salmo_trutta/Location/View?contigviewbottom=variation_feature_variation%3Dnormal;db=core;r=22:10134324-10134424;tl=RpFid1fPWgCVa7tG-11457638) | 10134374 | A | upstream_gene_variant | - | [ENSSTUG00000011995](https://www.ensembl.org/Salmo_trutta/Gene/Summary?db=core;g=ENSSTUG00000011995;tl=RpFid1fPWgCVa7tG-11457638) |
| [22](https://www.ensembl.org/Salmo_trutta/Location/View?contigviewbottom=variation_feature_variation%3Dnormal;db=core;r=22:15321645-15321745;tl=RpFid1fPWgCVa7tG-11457638) | 15321695 | C | downstream_gene_variant | sbno2b | [ENSSTUG00000043793](https://www.ensembl.org/Salmo_trutta/Gene/Summary?db=core;g=ENSSTUG00000043793;tl=RpFid1fPWgCVa7tG-11457638) |
| [22](https://www.ensembl.org/Salmo_trutta/Location/View?contigviewbottom=variation_feature_variation%3Dnormal;db=core;r=22:27912337-27912437;tl=RpFid1fPWgCVa7tG-11457638) | 27912387 | T | intron_variant, non_coding_transcript_variant | - | [ENSSTUG00000008021](https://www.ensembl.org/Salmo_trutta/Gene/Summary?db=core;g=ENSSTUG00000008021;tl=RpFid1fPWgCVa7tG-11457638) |
| [22](https://www.ensembl.org/Salmo_trutta/Location/View?contigviewbottom=variation_feature_variation%3Dnormal;db=core;r=22:34582908-34583008;tl=RpFid1fPWgCVa7tG-11457638) | 34582958 | T | intergenic_variant | - | - |
| [23](https://www.ensembl.org/Salmo_trutta/Location/View?contigviewbottom=variation_feature_variation%3Dnormal;db=core;r=23:1765970-1766070;tl=RpFid1fPWgCVa7tG-11457638) | 1766020 | G | intron_variant | notch1a | [ENSSTUG00000035570](https://www.ensembl.org/Salmo_trutta/Gene/Summary?db=core;g=ENSSTUG00000035570;tl=RpFid1fPWgCVa7tG-11457638) |
| [23](https://www.ensembl.org/Salmo_trutta/Location/View?contigviewbottom=variation_feature_variation%3Dnormal;db=core;r=23:8982844-8982944;tl=RpFid1fPWgCVa7tG-11457638) | 8982894 | T | downstream_gene_variant | SCD5 | [ENSSTUG00000014929](https://www.ensembl.org/Salmo_trutta/Gene/Summary?db=core;g=ENSSTUG00000014929;tl=RpFid1fPWgCVa7tG-11457638) |
| [23](https://www.ensembl.org/Salmo_trutta/Location/View?contigviewbottom=variation_feature_variation%3Dnormal;db=core;r=23:8982844-8982944;tl=RpFid1fPWgCVa7tG-11457638) | 8982894 | T | downstream_gene_variant | sec31a | [ENSSTUG00000013337](https://www.ensembl.org/Salmo_trutta/Gene/Summary?db=core;g=ENSSTUG00000013337;tl=RpFid1fPWgCVa7tG-11457638) |
| [23](https://www.ensembl.org/Salmo_trutta/Location/View?contigviewbottom=variation_feature_variation%3Dnormal;db=core;r=23:8982844-8982944;tl=RpFid1fPWgCVa7tG-11457638) | 8982894 | T | upstream_gene_variant | tmem150c | [ENSSTUG00000014936](https://www.ensembl.org/Salmo_trutta/Gene/Summary?db=core;g=ENSSTUG00000014936;tl=RpFid1fPWgCVa7tG-11457638) |
| [25](https://www.ensembl.org/Salmo_trutta/Location/View?contigviewbottom=variation_feature_variation%3Dnormal;db=core;r=25:28742740-28742840;tl=RpFid1fPWgCVa7tG-11457638) | 28742790 | G | intron_variant | - | [ENSSTUG00000010202](https://www.ensembl.org/Salmo_trutta/Gene/Summary?db=core;g=ENSSTUG00000010202;tl=RpFid1fPWgCVa7tG-11457638) |
| [25](https://www.ensembl.org/Salmo_trutta/Location/View?contigviewbottom=variation_feature_variation%3Dnormal;db=core;r=25:33689681-33689781;tl=RpFid1fPWgCVa7tG-11457638) | 33689731 | A | upstream_gene_variant | CLDN20 | [ENSSTUG00000003398](https://www.ensembl.org/Salmo_trutta/Gene/Summary?db=core;g=ENSSTUG00000003398;tl=RpFid1fPWgCVa7tG-11457638) |
| [25](https://www.ensembl.org/Salmo_trutta/Location/View?contigviewbottom=variation_feature_variation%3Dnormal;db=core;r=25:33689681-33689781;tl=RpFid1fPWgCVa7tG-11457638) | 33689731 | A | intron_variant | tfb1m | [ENSSTUG00000003383](https://www.ensembl.org/Salmo_trutta/Gene/Summary?db=core;g=ENSSTUG00000003383;tl=RpFid1fPWgCVa7tG-11457638) |
| [25](https://www.ensembl.org/Salmo_trutta/Location/View?contigviewbottom=variation_feature_variation%3Dnormal;db=core;r=25:45484654-45484754;tl=RpFid1fPWgCVa7tG-11457638) | 45484704 | C | intergenic_variant | - | - |
| [26](https://www.ensembl.org/Salmo_trutta/Location/View?contigviewbottom=variation_feature_variation%3Dnormal;db=core;r=26:7744018-7744118;tl=RpFid1fPWgCVa7tG-11457638) | 7744068 | T | intergenic_variant | - | - |
| [26](https://www.ensembl.org/Salmo_trutta/Location/View?contigviewbottom=variation_feature_variation%3Dnormal;db=core;r=26:8231147-8231247;tl=RpFid1fPWgCVa7tG-11457638) | 8231197 | T | intergenic_variant | - | - |
| [26](https://www.ensembl.org/Salmo_trutta/Location/View?contigviewbottom=variation_feature_variation%3Dnormal;db=core;r=26:22925316-22925416;tl=RpFid1fPWgCVa7tG-11457638) | 22925366 | A | synonymous_variant | taok1a | [ENSSTUG00000023371](https://www.ensembl.org/Salmo_trutta/Gene/Summary?db=core;g=ENSSTUG00000023371;tl=RpFid1fPWgCVa7tG-11457638) |
| [26](https://www.ensembl.org/Salmo_trutta/Location/View?contigviewbottom=variation_feature_variation%3Dnormal;db=core;r=26:28726381-28726481;tl=RpFid1fPWgCVa7tG-11457638) | 28726431 | G | upstream_gene_variant | mrps23 | [ENSSTUG00000011788](https://www.ensembl.org/Salmo_trutta/Gene/Summary?db=core;g=ENSSTUG00000011788;tl=RpFid1fPWgCVa7tG-11457638) |
| [26](https://www.ensembl.org/Salmo_trutta/Location/View?contigviewbottom=variation_feature_variation%3Dnormal;db=core;r=26:28726381-28726481;tl=RpFid1fPWgCVa7tG-11457638) | 28726431 | G | missense_variant | NOS2 | [ENSSTUG00000011802](https://www.ensembl.org/Salmo_trutta/Gene/Summary?db=core;g=ENSSTUG00000011802;tl=RpFid1fPWgCVa7tG-11457638) |
| [27](https://www.ensembl.org/Salmo_trutta/Location/View?contigviewbottom=variation_feature_variation%3Dnormal;db=core;r=27:4138672-4138772;tl=RpFid1fPWgCVa7tG-11457638) | 4138722 | A | downstream_gene_variant | zgc:101858 | [ENSSTUG00000016335](https://www.ensembl.org/Salmo_trutta/Gene/Summary?db=core;g=ENSSTUG00000016335;tl=RpFid1fPWgCVa7tG-11457638) |
| [27](https://www.ensembl.org/Salmo_trutta/Location/View?contigviewbottom=variation_feature_variation%3Dnormal;db=core;r=27:4138672-4138772;tl=RpFid1fPWgCVa7tG-11457638) | 4138722 | A | 3_prime_UTR_variant | - | [ENSSTUG00000016357](https://www.ensembl.org/Salmo_trutta/Gene/Summary?db=core;g=ENSSTUG00000016357;tl=RpFid1fPWgCVa7tG-11457638) |
| [27](https://www.ensembl.org/Salmo_trutta/Location/View?contigviewbottom=variation_feature_variation%3Dnormal;db=core;r=27:30706301-30706401;tl=RpFid1fPWgCVa7tG-11457638) | 30706351 | T | intron_variant | fibcd1a | [ENSSTUG00000035619](https://www.ensembl.org/Salmo_trutta/Gene/Summary?db=core;g=ENSSTUG00000035619;tl=RpFid1fPWgCVa7tG-11457638) |
| [27](https://www.ensembl.org/Salmo_trutta/Location/View?contigviewbottom=variation_feature_variation%3Dnormal;db=core;r=27:30781548-30781648;tl=RpFid1fPWgCVa7tG-11457638) | 30781598 | G | intron_variant | fibcd1a | [ENSSTUG00000035619](https://www.ensembl.org/Salmo_trutta/Gene/Summary?db=core;g=ENSSTUG00000035619;tl=RpFid1fPWgCVa7tG-11457638) |
| [27](https://www.ensembl.org/Salmo_trutta/Location/View?contigviewbottom=variation_feature_variation%3Dnormal;db=core;r=27:30781550-30781650;tl=RpFid1fPWgCVa7tG-11457638) | 30781600 | T | intron_variant | fibcd1a | [ENSSTUG00000035619](https://www.ensembl.org/Salmo_trutta/Gene/Summary?db=core;g=ENSSTUG00000035619;tl=RpFid1fPWgCVa7tG-11457638) |
| [27](https://www.ensembl.org/Salmo_trutta/Location/View?contigviewbottom=variation_feature_variation%3Dnormal;db=core;r=27:37237575-37237675;tl=RpFid1fPWgCVa7tG-11457638) | 37237625 | G | downstream_gene_variant | aptx | [ENSSTUG00000033812](https://www.ensembl.org/Salmo_trutta/Gene/Summary?db=core;g=ENSSTUG00000033812;tl=RpFid1fPWgCVa7tG-11457638) |
| [27](https://www.ensembl.org/Salmo_trutta/Location/View?contigviewbottom=variation_feature_variation%3Dnormal;db=core;r=27:37237575-37237675;tl=RpFid1fPWgCVa7tG-11457638) | 37237625 | G | upstream_gene_variant | elac1 | [ENSSTUG00000033819](https://www.ensembl.org/Salmo_trutta/Gene/Summary?db=core;g=ENSSTUG00000033819;tl=RpFid1fPWgCVa7tG-11457638) |
| [27](https://www.ensembl.org/Salmo_trutta/Location/View?contigviewbottom=variation_feature_variation%3Dnormal;db=core;r=27:37237575-37237675;tl=RpFid1fPWgCVa7tG-11457638) | 37237625 | G | intron_variant | me2 | [ENSSTUG00000031924](https://www.ensembl.org/Salmo_trutta/Gene/Summary?db=core;g=ENSSTUG00000031924;tl=RpFid1fPWgCVa7tG-11457638) |
| [27](https://www.ensembl.org/Salmo_trutta/Location/View?contigviewbottom=variation_feature_variation%3Dnormal;db=core;r=27:41296735-41296835;tl=RpFid1fPWgCVa7tG-11457638) | 41296785 | A | intron_variant | fras1 | [ENSSTUG00000004652](https://www.ensembl.org/Salmo_trutta/Gene/Summary?db=core;g=ENSSTUG00000004652;tl=RpFid1fPWgCVa7tG-11457638) |
| [27](https://www.ensembl.org/Salmo_trutta/Location/View?contigviewbottom=variation_feature_variation%3Dnormal;db=core;r=27:41296768-41296868;tl=RpFid1fPWgCVa7tG-11457638) | 41296818 | T | intron_variant | fras1 | [ENSSTUG00000004652](https://www.ensembl.org/Salmo_trutta/Gene/Summary?db=core;g=ENSSTUG00000004652;tl=RpFid1fPWgCVa7tG-11457638) |
| [27](https://www.ensembl.org/Salmo_trutta/Location/View?contigviewbottom=variation_feature_variation%3Dnormal;db=core;r=27:44851897-44851997;tl=RpFid1fPWgCVa7tG-11457638) | 44851947 | G | intron_variant | cfap299 | [ENSSTUG00000009726](https://www.ensembl.org/Salmo_trutta/Gene/Summary?db=core;g=ENSSTUG00000009726;tl=RpFid1fPWgCVa7tG-11457638) |
| [27](https://www.ensembl.org/Salmo_trutta/Location/View?contigviewbottom=variation_feature_variation%3Dnormal;db=core;r=27:44851909-44852009;tl=RpFid1fPWgCVa7tG-11457638) | 44851959 | C | intron_variant | cfap299 | [ENSSTUG00000009726](https://www.ensembl.org/Salmo_trutta/Gene/Summary?db=core;g=ENSSTUG00000009726;tl=RpFid1fPWgCVa7tG-11457638) |
| [28](https://www.ensembl.org/Salmo_trutta/Location/View?contigviewbottom=variation_feature_variation%3Dnormal;db=core;r=28:28814796-28814896;tl=RpFid1fPWgCVa7tG-11457638) | 28814846 | C | downstream_gene_variant | gss | [ENSSTUG00000046984](https://www.ensembl.org/Salmo_trutta/Gene/Summary?db=core;g=ENSSTUG00000046984;tl=RpFid1fPWgCVa7tG-11457638) |
| [28](https://www.ensembl.org/Salmo_trutta/Location/View?contigviewbottom=variation_feature_variation%3Dnormal;db=core;r=28:28814796-28814896;tl=RpFid1fPWgCVa7tG-11457638) | 28814846 | C | upstream_gene_variant | - | [ENSSTUG00000046981](https://www.ensembl.org/Salmo_trutta/Gene/Summary?db=core;g=ENSSTUG00000046981;tl=RpFid1fPWgCVa7tG-11457638) |
| [28](https://www.ensembl.org/Salmo_trutta/Location/View?contigviewbottom=variation_feature_variation%3Dnormal;db=core;r=28:31570886-31570986;tl=RpFid1fPWgCVa7tG-11457638) | 31570936 | A | intron_variant | klhl17 | [ENSSTUG00000036612](https://www.ensembl.org/Salmo_trutta/Gene/Summary?db=core;g=ENSSTUG00000036612;tl=RpFid1fPWgCVa7tG-11457638) |
| [28](https://www.ensembl.org/Salmo_trutta/Location/View?contigviewbottom=variation_feature_variation%3Dnormal;db=core;r=28:40119218-40119318;tl=RpFid1fPWgCVa7tG-11457638) | 40119268 | G | intergenic_variant | - | - |
| [30](https://www.ensembl.org/Salmo_trutta/Location/View?contigviewbottom=variation_feature_variation%3Dnormal;db=core;r=30:20717936-20718036;tl=RpFid1fPWgCVa7tG-11457638) | 20717986 | T | intron_variant | plekhm2 | [ENSSTUG00000010658](https://www.ensembl.org/Salmo_trutta/Gene/Summary?db=core;g=ENSSTUG00000010658;tl=RpFid1fPWgCVa7tG-11457638) |
| [30](https://www.ensembl.org/Salmo_trutta/Location/View?contigviewbottom=variation_feature_variation%3Dnormal;db=core;r=30:20717936-20718036;tl=RpFid1fPWgCVa7tG-11457638) | 20717986 | T | downstream_gene_variant | - | [ENSSTUG00000010569](https://www.ensembl.org/Salmo_trutta/Gene/Summary?db=core;g=ENSSTUG00000010569;tl=RpFid1fPWgCVa7tG-11457638) |
| [30](https://www.ensembl.org/Salmo_trutta/Location/View?contigviewbottom=variation_feature_variation%3Dnormal;db=core;r=30:27536549-27536649;tl=RpFid1fPWgCVa7tG-11457638) | 27536599 | A | intron_variant | nup210 | [ENSSTUG00000005830](https://www.ensembl.org/Salmo_trutta/Gene/Summary?db=core;g=ENSSTUG00000005830;tl=RpFid1fPWgCVa7tG-11457638) |
| [30](https://www.ensembl.org/Salmo_trutta/Location/View?contigviewbottom=variation_feature_variation%3Dnormal;db=core;r=30:28193153-28193253;tl=RpFid1fPWgCVa7tG-11457638) | 28193203 | T | intron_variant | WNK2 | [ENSSTUG00000007335](https://www.ensembl.org/Salmo_trutta/Gene/Summary?db=core;g=ENSSTUG00000007335;tl=RpFid1fPWgCVa7tG-11457638) |
| [30](https://www.ensembl.org/Salmo_trutta/Location/View?contigviewbottom=variation_feature_variation%3Dnormal;db=core;r=30:42281239-42281339;tl=RpFid1fPWgCVa7tG-11457638) | 42281289 | T | intergenic_variant | - | - |
| [31](https://www.ensembl.org/Salmo_trutta/Location/View?contigviewbottom=variation_feature_variation%3Dnormal;db=core;r=31:4501029-4501129;tl=RpFid1fPWgCVa7tG-11457638) | 4501079 | T | missense_variant | - | [ENSSTUG00000018616](https://www.ensembl.org/Salmo_trutta/Gene/Summary?db=core;g=ENSSTUG00000018616;tl=RpFid1fPWgCVa7tG-11457638) |
| [31](https://www.ensembl.org/Salmo_trutta/Location/View?contigviewbottom=variation_feature_variation%3Dnormal;db=core;r=31:21077888-21077988;tl=RpFid1fPWgCVa7tG-11457638) | 21077938 | T | intergenic_variant | - | - |
| [31](https://www.ensembl.org/Salmo_trutta/Location/View?contigviewbottom=variation_feature_variation%3Dnormal;db=core;r=31:25387664-25387764;tl=RpFid1fPWgCVa7tG-11457638) | 25387714 | A | intergenic_variant | - | - |
| [31](https://www.ensembl.org/Salmo_trutta/Location/View?contigviewbottom=variation_feature_variation%3Dnormal;db=core;r=31:29820039-29820139;tl=RpFid1fPWgCVa7tG-11457638) | 29820089 | T | upstream_gene_variant | - | [ENSSTUG00000037686](https://www.ensembl.org/Salmo_trutta/Gene/Summary?db=core;g=ENSSTUG00000037686;tl=RpFid1fPWgCVa7tG-11457638) |
| [31](https://www.ensembl.org/Salmo_trutta/Location/View?contigviewbottom=variation_feature_variation%3Dnormal;db=core;r=31:36266302-36266402;tl=RpFid1fPWgCVa7tG-11457638) | 36266352 | C | intron_variant | nyap2b | [ENSSTUG00000006047](https://www.ensembl.org/Salmo_trutta/Gene/Summary?db=core;g=ENSSTUG00000006047;tl=RpFid1fPWgCVa7tG-11457638) |
| [32](https://www.ensembl.org/Salmo_trutta/Location/View?contigviewbottom=variation_feature_variation%3Dnormal;db=core;r=32:43610120-43610220;tl=RpFid1fPWgCVa7tG-11457638) | 43610170 | G | upstream_gene_variant | clcn7 | [ENSSTUG00000017389](https://www.ensembl.org/Salmo_trutta/Gene/Summary?db=core;g=ENSSTUG00000017389;tl=RpFid1fPWgCVa7tG-11457638) |
| [32](https://www.ensembl.org/Salmo_trutta/Location/View?contigviewbottom=variation_feature_variation%3Dnormal;db=core;r=32:43610120-43610220;tl=RpFid1fPWgCVa7tG-11457638) | 43610170 | G | intron_variant | ptx4 | [ENSSTUG00000017634](https://www.ensembl.org/Salmo_trutta/Gene/Summary?db=core;g=ENSSTUG00000017634;tl=RpFid1fPWgCVa7tG-11457638) |
| [33](https://www.ensembl.org/Salmo_trutta/Location/View?contigviewbottom=variation_feature_variation%3Dnormal;db=core;r=33:27832650-27832750;tl=RpFid1fPWgCVa7tG-11457638) | 27832700 | A | downstream_gene_variant | iars2 | [ENSSTUG00000043752](https://www.ensembl.org/Salmo_trutta/Gene/Summary?db=core;g=ENSSTUG00000043752;tl=RpFid1fPWgCVa7tG-11457638) |
| [33](https://www.ensembl.org/Salmo_trutta/Location/View?contigviewbottom=variation_feature_variation%3Dnormal;db=core;r=33:27832650-27832750;tl=RpFid1fPWgCVa7tG-11457638) | 27832700 | A | missense_variant | rab3gap2 | [ENSSTUG00000043952](https://www.ensembl.org/Salmo_trutta/Gene/Summary?db=core;g=ENSSTUG00000043952;tl=RpFid1fPWgCVa7tG-11457638) |
| [34](https://www.ensembl.org/Salmo_trutta/Location/View?contigviewbottom=variation_feature_variation%3Dnormal;db=core;r=34:211619-211719;tl=RpFid1fPWgCVa7tG-11457638) | 211669 | A | intron_variant | PIP5K1A | [ENSSTUG00000040903](https://www.ensembl.org/Salmo_trutta/Gene/Summary?db=core;g=ENSSTUG00000040903;tl=RpFid1fPWgCVa7tG-11457638) |
| [34](https://www.ensembl.org/Salmo_trutta/Location/View?contigviewbottom=variation_feature_variation%3Dnormal;db=core;r=34:10654069-10654169;tl=RpFid1fPWgCVa7tG-11457638) | 10654119 | C | downstream_gene_variant | brd2a | [ENSSTUG00000033342](https://www.ensembl.org/Salmo_trutta/Gene/Summary?db=core;g=ENSSTUG00000033342;tl=RpFid1fPWgCVa7tG-11457638) |
| [34](https://www.ensembl.org/Salmo_trutta/Location/View?contigviewbottom=variation_feature_variation%3Dnormal;db=core;r=34:10654069-10654169;tl=RpFid1fPWgCVa7tG-11457638) | 10654119 | C | downstream_gene_variant | col11a2 | [ENSSTUG00000033381](https://www.ensembl.org/Salmo_trutta/Gene/Summary?db=core;g=ENSSTUG00000033381;tl=RpFid1fPWgCVa7tG-11457638) |
| [34](https://www.ensembl.org/Salmo_trutta/Location/View?contigviewbottom=variation_feature_variation%3Dnormal;db=core;r=34:10654069-10654169;tl=RpFid1fPWgCVa7tG-11457638) | 10654119 | C | synonymous_variant | - | [ENSSTUG00000033349](https://www.ensembl.org/Salmo_trutta/Gene/Summary?db=core;g=ENSSTUG00000033349;tl=RpFid1fPWgCVa7tG-11457638) |
| [34](https://www.ensembl.org/Salmo_trutta/Location/View?contigviewbottom=variation_feature_variation%3Dnormal;db=core;r=34:10654075-10654175;tl=RpFid1fPWgCVa7tG-11457638) | 10654125 | A | downstream_gene_variant | brd2a | [ENSSTUG00000033342](https://www.ensembl.org/Salmo_trutta/Gene/Summary?db=core;g=ENSSTUG00000033342;tl=RpFid1fPWgCVa7tG-11457638) |
| [34](https://www.ensembl.org/Salmo_trutta/Location/View?contigviewbottom=variation_feature_variation%3Dnormal;db=core;r=34:10654075-10654175;tl=RpFid1fPWgCVa7tG-11457638) | 10654125 | A | downstream_gene_variant | col11a2 | [ENSSTUG00000033381](https://www.ensembl.org/Salmo_trutta/Gene/Summary?db=core;g=ENSSTUG00000033381;tl=RpFid1fPWgCVa7tG-11457638) |
| [34](https://www.ensembl.org/Salmo_trutta/Location/View?contigviewbottom=variation_feature_variation%3Dnormal;db=core;r=34:10654075-10654175;tl=RpFid1fPWgCVa7tG-11457638) | 10654125 | A | synonymous_variant | - | [ENSSTUG00000033349](https://www.ensembl.org/Salmo_trutta/Gene/Summary?db=core;g=ENSSTUG00000033349;tl=RpFid1fPWgCVa7tG-11457638) |
| [34](https://www.ensembl.org/Salmo_trutta/Location/View?contigviewbottom=variation_feature_variation%3Dnormal;db=core;r=34:10938930-10939030;tl=RpFid1fPWgCVa7tG-11457638) | 10938980 | T | upstream_gene_variant | - | [ENSSTUG00000035732](https://www.ensembl.org/Salmo_trutta/Gene/Summary?db=core;g=ENSSTUG00000035732;tl=RpFid1fPWgCVa7tG-11457638) |
| [34](https://www.ensembl.org/Salmo_trutta/Location/View?contigviewbottom=variation_feature_variation%3Dnormal;db=core;r=34:14439054-14439154;tl=RpFid1fPWgCVa7tG-11457638) | 14439104 | C | intergenic_variant | - | - |
| [34](https://www.ensembl.org/Salmo_trutta/Location/View?contigviewbottom=variation_feature_variation%3Dnormal;db=core;r=34:16962567-16962667;tl=RpFid1fPWgCVa7tG-11457638) | 16962617 | T | intron_variant | jarid2b | [ENSSTUG00000048175](https://www.ensembl.org/Salmo_trutta/Gene/Summary?db=core;g=ENSSTUG00000048175;tl=RpFid1fPWgCVa7tG-11457638) |
| [34](https://www.ensembl.org/Salmo_trutta/Location/View?contigviewbottom=variation_feature_variation%3Dnormal;db=core;r=34:35506578-35506678;tl=RpFid1fPWgCVa7tG-11457638) | 35506628 | T | intergenic_variant | - | - |
| [34](https://www.ensembl.org/Salmo_trutta/Location/View?contigviewbottom=variation_feature_variation%3Dnormal;db=core;r=34:35506579-35506679;tl=RpFid1fPWgCVa7tG-11457638) | 35506629 | G | intergenic_variant | - | - |
| [34](https://www.ensembl.org/Salmo_trutta/Location/View?contigviewbottom=variation_feature_variation%3Dnormal;db=core;r=34:35506580-35506680;tl=RpFid1fPWgCVa7tG-11457638) | 35506630 | A | intergenic_variant | - | - |
| [34](https://www.ensembl.org/Salmo_trutta/Location/View?contigviewbottom=variation_feature_variation%3Dnormal;db=core;r=34:35506581-35506681;tl=RpFid1fPWgCVa7tG-11457638) | 35506631 | C | intergenic_variant | - | - |
| [35](https://www.ensembl.org/Salmo_trutta/Location/View?contigviewbottom=variation_feature_variation%3Dnormal;db=core;r=35:5304171-5304271;tl=RpFid1fPWgCVa7tG-11457638) | 5304221 | T | intron_variant | PLCB4 | [ENSSTUG00000023418](https://www.ensembl.org/Salmo_trutta/Gene/Summary?db=core;g=ENSSTUG00000023418;tl=RpFid1fPWgCVa7tG-11457638) |
| [35](https://www.ensembl.org/Salmo_trutta/Location/View?contigviewbottom=variation_feature_variation%3Dnormal;db=core;r=35:11043718-11043818;tl=RpFid1fPWgCVa7tG-11457638) | 11043768 | T | intron_variant | gphnb | [ENSSTUG00000050478](https://www.ensembl.org/Salmo_trutta/Gene/Summary?db=core;g=ENSSTUG00000050478;tl=RpFid1fPWgCVa7tG-11457638) |
| [35](https://www.ensembl.org/Salmo_trutta/Location/View?contigviewbottom=variation_feature_variation%3Dnormal;db=core;r=35:16813517-16813617;tl=RpFid1fPWgCVa7tG-11457638) | 16813567 | C | intergenic_variant | - | - |
| [36](https://www.ensembl.org/Salmo_trutta/Location/View?contigviewbottom=variation_feature_variation%3Dnormal;db=core;r=36:32084016-32084116;tl=RpFid1fPWgCVa7tG-11457638) | 32084066 | T | synonymous_variant | ATAD2 | [ENSSTUG00000031100](https://www.ensembl.org/Salmo_trutta/Gene/Summary?db=core;g=ENSSTUG00000031100;tl=RpFid1fPWgCVa7tG-11457638) |
| [36](https://www.ensembl.org/Salmo_trutta/Location/View?contigviewbottom=variation_feature_variation%3Dnormal;db=core;r=36:32084016-32084116;tl=RpFid1fPWgCVa7tG-11457638) | 32084066 | T | upstream_gene_variant | ZHX1 | [ENSSTUG00000031095](https://www.ensembl.org/Salmo_trutta/Gene/Summary?db=core;g=ENSSTUG00000031095;tl=RpFid1fPWgCVa7tG-11457638) |
| [36](https://www.ensembl.org/Salmo_trutta/Location/View?contigviewbottom=variation_feature_variation%3Dnormal;db=core;r=36:35258900-35259000;tl=RpFid1fPWgCVa7tG-11457638) | 35258950 | G | synonymous_variant | c36h1orf216 | [ENSSTUG00000034985](https://www.ensembl.org/Salmo_trutta/Gene/Summary?db=core;g=ENSSTUG00000034985;tl=RpFid1fPWgCVa7tG-11457638) |
| [36](https://www.ensembl.org/Salmo_trutta/Location/View?contigviewbottom=variation_feature_variation%3Dnormal;db=core;r=36:35258900-35259000;tl=RpFid1fPWgCVa7tG-11457638) | 35258950 | G | upstream_gene_variant | psmb2 | [ENSSTUG00000034970](https://www.ensembl.org/Salmo_trutta/Gene/Summary?db=core;g=ENSSTUG00000034970;tl=RpFid1fPWgCVa7tG-11457638) |
| [37](https://www.ensembl.org/Salmo_trutta/Location/View?contigviewbottom=variation_feature_variation%3Dnormal;db=core;r=37:34610304-34610404;tl=RpFid1fPWgCVa7tG-11457638) | 34610354 | C | intergenic_variant | - | - |
| [38](https://www.ensembl.org/Salmo_trutta/Location/View?contigviewbottom=variation_feature_variation%3Dnormal;db=core;r=38:6437447-6437547;tl=RpFid1fPWgCVa7tG-11457638) | 6437497 | T | upstream_gene_variant | KCNJ4 | [ENSSTUG00000040061](https://www.ensembl.org/Salmo_trutta/Gene/Summary?db=core;g=ENSSTUG00000040061;tl=RpFid1fPWgCVa7tG-11457638) |
| [38](https://www.ensembl.org/Salmo_trutta/Location/View?contigviewbottom=variation_feature_variation%3Dnormal;db=core;r=38:6437447-6437547;tl=RpFid1fPWgCVa7tG-11457638) | 6437497 | T | intron_variant, non_coding_transcript_variant | - | [ENSSTUG00000040072](https://www.ensembl.org/Salmo_trutta/Gene/Summary?db=core;g=ENSSTUG00000040072;tl=RpFid1fPWgCVa7tG-11457638) |
| [38](https://www.ensembl.org/Salmo_trutta/Location/View?contigviewbottom=variation_feature_variation%3Dnormal;db=core;r=38:14810400-14810500;tl=RpFid1fPWgCVa7tG-11457638) | 14810450 | A | downstream_gene_variant | - | [ENSSTUG00000022746](https://www.ensembl.org/Salmo_trutta/Gene/Summary?db=core;g=ENSSTUG00000022746;tl=RpFid1fPWgCVa7tG-11457638) |
| [38](https://www.ensembl.org/Salmo_trutta/Location/View?contigviewbottom=variation_feature_variation%3Dnormal;db=core;r=38:19678014-19678114;tl=RpFid1fPWgCVa7tG-11457638) | 19678064 | T | intron_variant | si:ch73-173h19.3 | [ENSSTUG00000016683](https://www.ensembl.org/Salmo_trutta/Gene/Summary?db=core;g=ENSSTUG00000016683;tl=RpFid1fPWgCVa7tG-11457638) |
| [39](https://www.ensembl.org/Salmo_trutta/Location/View?contigviewbottom=variation_feature_variation%3Dnormal;db=core;r=39:17902659-17902759;tl=RpFid1fPWgCVa7tG-11457638) | 17902709 | C | upstream_gene_variant | clcn4 | [ENSSTUG00000047114](https://www.ensembl.org/Salmo_trutta/Gene/Summary?db=core;g=ENSSTUG00000047114;tl=RpFid1fPWgCVa7tG-11457638) |
| [39](https://www.ensembl.org/Salmo_trutta/Location/View?contigviewbottom=variation_feature_variation%3Dnormal;db=core;r=39:17902659-17902759;tl=RpFid1fPWgCVa7tG-11457638) | 17902709 | C | downstream_gene_variant | SHROOM2 | [ENSSTUG00000047015](https://www.ensembl.org/Salmo_trutta/Gene/Summary?db=core;g=ENSSTUG00000047015;tl=RpFid1fPWgCVa7tG-11457638) |
| [39](https://www.ensembl.org/Salmo_trutta/Location/View?contigviewbottom=variation_feature_variation%3Dnormal;db=core;r=39:17902659-17902759;tl=RpFid1fPWgCVa7tG-11457638) | 17902709 | C | downstream_gene_variant | - | [ENSSTUG00000047027](https://www.ensembl.org/Salmo_trutta/Gene/Summary?db=core;g=ENSSTUG00000047027;tl=RpFid1fPWgCVa7tG-11457638) |
| [39](https://www.ensembl.org/Salmo_trutta/Location/View?contigviewbottom=variation_feature_variation%3Dnormal;db=core;r=39:17902659-17902759;tl=RpFid1fPWgCVa7tG-11457638) | 17902709 | C | intron_variant | - | [ENSSTUG00000047030](https://www.ensembl.org/Salmo_trutta/Gene/Summary?db=core;g=ENSSTUG00000047030;tl=RpFid1fPWgCVa7tG-11457638) |
| [39](https://www.ensembl.org/Salmo_trutta/Location/View?contigviewbottom=variation_feature_variation%3Dnormal;db=core;r=39:21292162-21292262;tl=RpFid1fPWgCVa7tG-11457638) | 21292212 | G | intron_variant | pknox1.2 | [ENSSTUG00000040250](https://www.ensembl.org/Salmo_trutta/Gene/Summary?db=core;g=ENSSTUG00000040250;tl=RpFid1fPWgCVa7tG-11457638) |
| [39](https://www.ensembl.org/Salmo_trutta/Location/View?contigviewbottom=variation_feature_variation%3Dnormal;db=core;r=39:21292162-21292262;tl=RpFid1fPWgCVa7tG-11457638) | 21292212 | G | downstream_gene_variant | tmprss3b | [ENSSTUG00000040308](https://www.ensembl.org/Salmo_trutta/Gene/Summary?db=core;g=ENSSTUG00000040308;tl=RpFid1fPWgCVa7tG-11457638) |
| [39](https://www.ensembl.org/Salmo_trutta/Location/View?contigviewbottom=variation_feature_variation%3Dnormal;db=core;r=39:21292162-21292262;tl=RpFid1fPWgCVa7tG-11457638) | 21292212 | G | downstream_gene_variant | - | [ENSSTUG00000040236](https://www.ensembl.org/Salmo_trutta/Gene/Summary?db=core;g=ENSSTUG00000040236;tl=RpFid1fPWgCVa7tG-11457638) |
| [39](https://www.ensembl.org/Salmo_trutta/Location/View?contigviewbottom=variation_feature_variation%3Dnormal;db=core;r=39:25277595-25277695;tl=RpFid1fPWgCVa7tG-11457638) | 25277645 | T | downstream_gene_variant | - | [ENSSTUG00000041279](https://www.ensembl.org/Salmo_trutta/Gene/Summary?db=core;g=ENSSTUG00000041279;tl=RpFid1fPWgCVa7tG-11457638) |
| [40](https://www.ensembl.org/Salmo_trutta/Location/View?contigviewbottom=variation_feature_variation%3Dnormal;db=core;r=40:17101981-17102081;tl=RpFid1fPWgCVa7tG-11457638) | 17102031 | C | downstream_gene_variant | lyrm5a | [ENSSTUG00000048658](https://www.ensembl.org/Salmo_trutta/Gene/Summary?db=core;g=ENSSTUG00000048658;tl=RpFid1fPWgCVa7tG-11457638) |
| [40](https://www.ensembl.org/Salmo_trutta/Location/View?contigviewbottom=variation_feature_variation%3Dnormal;db=core;r=40:17102024-17102124;tl=RpFid1fPWgCVa7tG-11457638) | 17102074 | G | downstream_gene_variant | lyrm5a | [ENSSTUG00000048658](https://www.ensembl.org/Salmo_trutta/Gene/Summary?db=core;g=ENSSTUG00000048658;tl=RpFid1fPWgCVa7tG-11457638) |
| [40](https://www.ensembl.org/Salmo_trutta/Location/View?contigviewbottom=variation_feature_variation%3Dnormal;db=core;r=40:22174270-22174370;tl=RpFid1fPWgCVa7tG-11457638) | 22174320 | T | intergenic_variant | - | - |
